# Preclinical studies and transcriptome analysis in a model of Parkinson’s disease with dopaminergic ZNF746 expression

**DOI:** 10.1186/s13024-025-00814-3

**Published:** 2025-02-28

**Authors:** Ji Hun Kim, Sumin Yang, Hyojung Kim, Dang-Khoa Vo, Han-Joo Maeng, Areum Jo, Joo-Heon Shin, Joo-Ho Shin, Hyeon-Man Baek, Gum Hwa Lee, Sung-Hyun Kim, Key-Hwan Lim, Valina L. Dawson, Ted M. Dawson, Jae-Yeol Joo, Yunjong Lee

**Affiliations:** 1 Department of Pharmacology, Sungkyunkwan University School of Medicine, Samsung Biomedical Research Institute (SBRI), Suwon, 16419 Republic of Korea; 2 Department of Pharmacy, College of Pharmacy, Hanyang University, Ansan, 15588 Republic of Korea; 3 College of Pharmacy, Gachon University, Incheon, 21936 Republic of Korea; 4 Lieber Institute for Brain Development, Johns Hopkins Medical Campus, Baltimore, MD 21205 USA; 5 Department of Health Sciences & Technology, Gachon Advanced Institute for Health Sciences and Technology (GAIHST), Gachon University, Incheon, 21999 Republic of Korea; 6 College of Pharmacy, Chosun University, Gwangju, 61452 Republic of Korea; 7 Department of Pharmacy, College of Pharmacy, Chungbuk National University, Cheongju-Si, 28160 Republic of Korea; 8 Neuroregeneration and Stem Cell Programs, Institute for Cell Engineering, Johns Hopkins University School of Medicine, Baltimore, MD 21205 USA; 9 Department of Physiology, Johns Hopkins University School of Medicine, Baltimore, MD 21205 USA; 10 Department of Neurology, Johns Hopkins University School of Medicine, Baltimore, MD 21205 USA; 11 Solomon H. Snyder Department of Neuroscience, Johns Hopkins University School of Medicine, Baltimore, MD 21205 USA; 12 Department of Pharmacology and Molecular Sciences, Johns Hopkins University School of Medicine, Baltimore, MD 21205 USA

**Keywords:** Parkinson’s disease, PARIS, Conditional transgenic model, C-Abl, Ventral midbrain transcriptome

## Abstract

**Background:**

The parkin-interacting substrate (PARIS, also known as ZNF746) is a transcriptional repressor, whose accumulation and phosphorylation play central pathological roles in Parkinson’s disease (PD). PARIS-induced transcriptional repression of PGC-1α or MDM4 contributes to mitochondrial dysfunction and p53-dependent neuron loss in PD. Despite the important role of PARIS in PD pathogenesis, unbiased transcriptomic profiles influenced by PARIS accumulation in dopaminergic neurons remain unexplored.

**Methods:**

We engineered Tet-Off conditional transgenic mice expressing PARIS in dopaminergic neurons, driven by DAT-PF-tTA driver mice. The conditional PARIS transgenic mice were characterized by PD-associated pathologies, including progressive dopamine cell loss, neuroinflammation, PGC-1α repression, and mitochondrial proteome alteration. Motor impairment was assessed using pole and rotarod tests. L-DOPA and c-Abl inhibitors were administered to PARIS transgenic mice to evaluate their therapeutic efficacy. The transcriptomic profiles and gene ontology clusters were analyzed by bulk and single-nucleus RNA-seq for the ventral midbrains from PARIS transgenic and age-matched controls.

**Results:**

Conditional dopaminergic PARIS expression in mice led to the robust and selective dopaminergic neuron degeneration, neuroinflammation, and striatal dopamine deficits, resulting in L-DOPA-responsive motor impairments. Consistent with the results of previous reports, PARIS suppressed dopaminergic PGC-1α expression, disturbed mitochondrial marker protein expression, and reduced COXIV-labeled mitochondria in dopamine neurons. Pharmacological inhibition of c-Abl activity in PARIS transgenic mice largely prevents PD-associated pathological features. Unbiased transcriptomic analysis revealed PARIS-regulated differentially expressed genes (DEGs), both collectively and in a cell-type-specific manner, along with enriched biological pathways linked to PD pathogenesis. Single-cell resolution transcriptomic analysis confirmed repression of PGC-1α and several mitochondria-related target genes in dopaminergic cells. Additionally, we identified distinct glial cell subpopulations and DEGs associated with PD pathogenesis.

**Conclusions:**

Conditional PARIS transgenic mice recapitulate robust and dopaminergic neuron-selective pathological features of PD, allowing the preclinical evaluation of antisymptomatic and disease-modifying therapeutic strategies within a couple of months. Based on this new PD mouse model, we provide unbiased bulk and single-nucleus transcriptomic profiles that are regulated by PARIS and potentially contribute to PD pathogenesis. A PD mouse model with flexible pathology induction capacity and a whole transcriptome could serve as a useful resource for translational PD research.

**Supplementary Information:**

The online version contains supplementary material available at 10.1186/s13024-025-00814-3.

## Introduction

Parkinson’s disease (PD) is the most common neurodegenerative disorder worldwide. Pathologically, PD is characterized by substantial and selective loss of up to 70% dopaminergic neurons in the substantia nigra, presence of Lewy bodies, inflammation, and mitochondrial dysfunction [1, 2]. Currently, no effective therapies can halt or reverse the progressive development of the key pathological features of PD. PD-linked genetic mutations have been used to generate genetic disease mouse models critically important for identifying disease-associated pathways in vivo [3, 4]. Based on this pathologic signature, preclinical evaluation of certain therapeutic strategies can be determined. Optimal PD mouse models should recapitulate the key pathological hallmarks of PD with robust motor dysfunction [3]. The most common recessive PD gene, parkin [5], has been knocked out in mice to model autosomal recessive juvenile PD. However, parkin-knockout mice failed to produce any meaningful PD-related pathological features, such as dopamine neuron loss and Lewy body formation in the ventral midbrains (VM) [6]. Since inactivation of parkin’s E3 ligase activity results in the accumulation of downstream parkin substrates (aminoacyl tRNA synthetase interacting multifunctional protein 2 [AIMP2], parkin-interacting substrate [PARIS], and Parkin-associated endothelin receptor-like receptor), transgenic mice expressing these pathological parkin substrates have been developed to model PD with parkin inactivation [7–9]. However, these models express these pathological substrates in whole brain regions, and the extent of dopamine neuron loss is not in the range found in clinical PD. Therefore, a conditional genetic mouse model with a confined pathology of dopaminergic neurons and robust pathologies comparable to those of clinical PD should be developed.

PARIS (also known as ZNF746) is a parkin substrate that accumulates in the postmortem brains of patients with sporadic and familial PD with parkin inactivation [10]. PARIS phosphorylation by *PINK1*, another PD gene, facilitates its polyubiquitination by parkin and subsequent proteasomal degradation [11]. Thus, PARIS accumulation simulates the PD environment of parkin or PINK1 dysfunction. PARIS is a transcriptional repressor with a KRAB domain at the N-terminus, and its accumulation represses diverse genes important for regulating mitochondrial biogenesis, antioxidant defense, and apoptosis [10, 12], which are associated with PD pathologies. PARIS-mediated epigenetic repression of diverse target genes is regulated by the c-Abl phosphorylation of PARIS at Y137 [12]. Aberrant c-Abl activity has been implicated in PD pathogenesis [13–18], and pharmacological inhibition of c-Abl has been shown to be neuroprotective in diverse PD animal models, including that of virally induced PARIS brain expression [12, 19, 20]. Moreover, α-synuclein aggregation and dopaminergic neurotoxicity requires PARIS accumulation because α-synuclein preformed fibril inoculation into brain failed to produce α-synucleinopathy in PARIS-knockout background [21]. Owing to the pathological relevance and importance of PARIS, transcriptomic profiles affected by PARIS accumulation would be informative for understanding unbiased target biological pathways. Although PARIS targets have been studied at the transcriptomic level in SH-SY5Y cells [12], PD relevant transcriptomic signature of dopaminergic neurons have not been determined in vivo owing to the lack of available mouse models.

In this study, we engineered tetracycline-regulatable conditional transgenic mice that selectively express PARIS in dopaminergic neurons. This novel *PARIS* transgenic mice successfully recapitulate key PD pathologies including selective, robust, and progressive dopaminergic neuron loss, dopamine depletion in the striatum, mitochondrial dysfunction, α-synuclein aggregation, and neuroinflammation. The validity of *PARIS* transgenic mice as a practical preclinical PD mouse model was demonstrated using L-DOPA and nilotinib administration as a representative symptomatic and disease-modifying therapy. Finally, the ventral midbrain transcriptomic profiles of *PARIS* transgenic mice were analyzed using RNA-seq and single-nucleus RNA-seq, which revealed alterations in diverse biological pathways that might provide insights into PD pathogenesis and potentially serve as therapeutic targets.

## Results

### Generation of Tet-off conditional transgenic (Tg) mice expressing PARIS in dopaminergic neurons

**Fig. 1:**
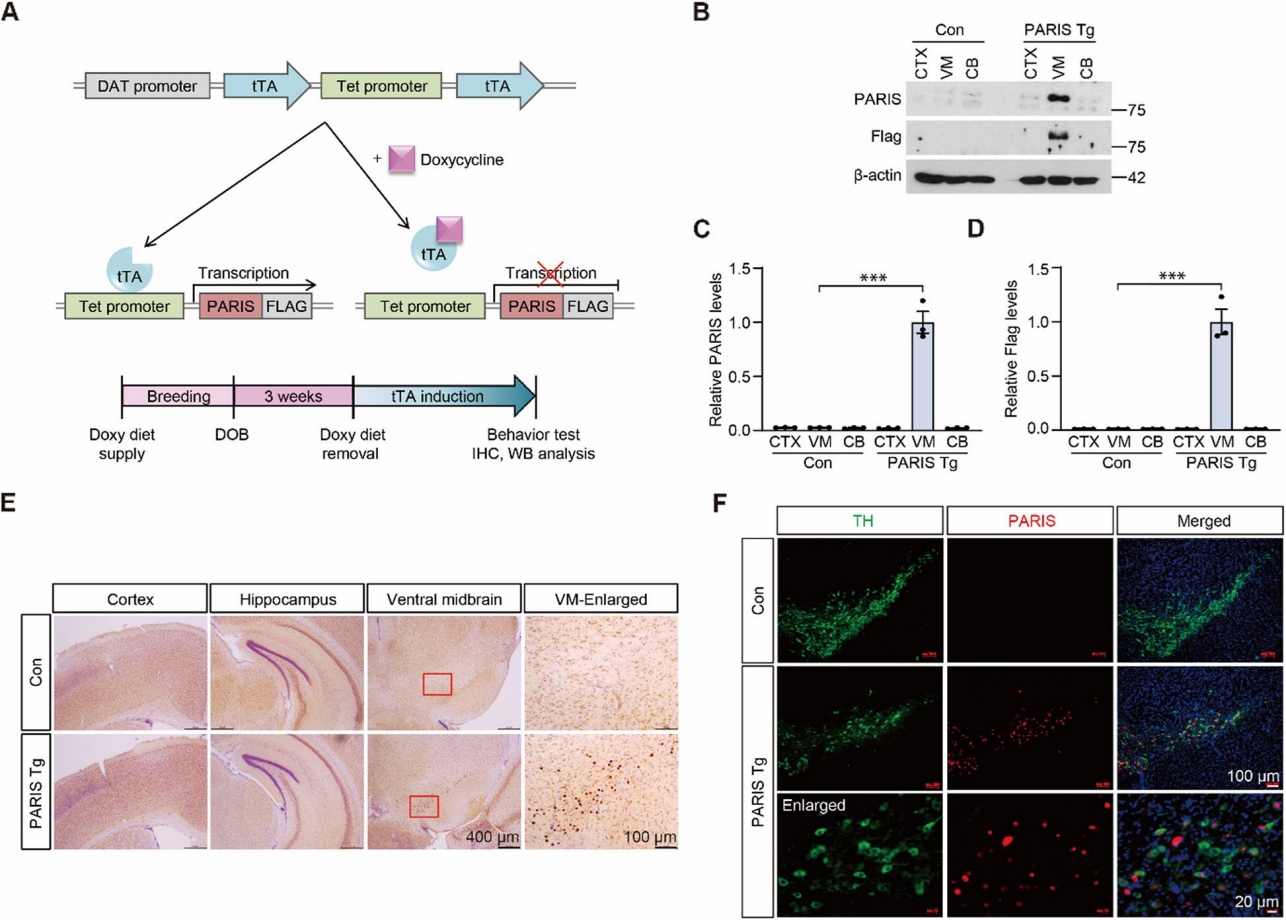
Generation and characterization of conditional transgenic mice expressing parkin-interacting substrate (PARIS) in dopaminergic neurons. **A** Schematic of tetracycline-regulatable expression of C-terminal FLAG-tagged PARIS (PARIS–FLAG). PARIS–FLAG expression from human *PARIS* transgene under the control of the Tet promoter (TetP-PARIS) construct is driven by dopaminergic neuron-specific tetracycline-regulatable transcription activator (tTA) expression from dopamine transporter-positive feedforward (DAT-PF)-tTA construct (upper panel). Experimental schedule of PARIS induction in transgenic mice with DAT-PF-tTA and TetP-PARIS (PARIS Tg mice) (bottom panel). **B** Representative western blot of PARIS, and FLAG expression in the cortex (CTX), ventral midbrain (VM), and cerebellum (CB) of PARIS transgenic mice (Tg) and age-matched littermate controls (control). **C**, **D** Quantification of PARIS, and FLAG protein expression in the indicated subregions of PARIS Tg mice and littermate controls normalized to β-actin (*n* = 3 mice per group). **E** PARIS distribution in brain sections from PARIS Tg mice and littermate controls monitored by immunohistochemistry using PARIS antibody. Magnified images of boxed regions from the ventral midbrain are shown in the right column. Scale bar: 400 μm, and 100 μm (enlarged images), respectively. **F** Immunofluorescent images of PARIS (red) and tyrosine hydroxylase (TH, green) from ventral midbrain sections of PARIS Tg and control mice. Bottom row, magnified images. Scale bar: 100 μm, and 20 μm (enlarged images), respectively. Data in all panels are mean ± standard error of the mean. ***P < 0.001, one-way analysis of variance (ANOVA) test followed by Tukey’s post hoc analysis

To explore the pathological roles of PARIS, the parkin substrate, in dopaminergic neurons in vivo, we employed a conditional tetracycline-regulatable transgenic approach that allowed transgene expression with spatial and temporal resolution. Responder mice were engineered to express the C-terminal FLAG-tagged human *PARIS* transgene under the control of the Tet promoter (TetP), which was activated by tetracycline-regulatable transcription activator (tTA) (Fig. 1A). To induce TetP activation only in dopaminergic neurons, the responder mice were crossed with the driver mice expressing tTA under the control of the dopamine transporter (DAT) promoter (Fig. 1A and Supplementary Fig. 1A). This driver knock-in mice that contain the tTA expression cassette downstream of the endogenous DAT promoter, and tTA expression is further augmented by the downstream TetP-tTA element (DAT-positive feedforward (PF)-tTA, heterozygous knock-in) ([22], Fig. 1A). The transgenic mice with DAT-PF-tTA and TetP-PARIS (PARIS Tg) express PARIS in dopaminergic neurons. DAT-PF-tTA mice were subsequently used as littermate controls (Supplementary Fig. 1A). Initially, heterozygous DAT-PF-tTA and TetP*-PARIS* mice were crossed and PARIS Tg and littermate control mice were obtained using tail genomic DNA genotyping PCR (Supplementary Fig. 1B). When the breeding was done under regular diet, the ratio of PARIS Tg was lower than expected (~ 10% from breeding of heterozygous DAT-PF-tTA and heterozygous TetP-PARIS). Almost no dopaminergic neurons survived in the ventral midbrain of 1-month-old PARIS Tg mice (Supplementary Fig. 1C, D). Consistent with this result, dopaminergic axon terminals were absent in the striata of PARIS Tg mice, whereas DAT-PF-tTA control mice had intact dopaminergic axon terminals (Supplementary Fig. 1E, F). The open-field behavior test showed reduced total exploration distance and increased anxiety in PARIS Tg mice compared to that in littermate controls (Supplementary Fig. 1G–I). PARIS Tg mice also exhibited defects in motor coordination, with no overt bradykinesia in the pole test (Supplementary Fig. 1 J, K). Together, these results indicate that PARIS expression in dopaminergic neurons during development is neurotoxic and impairs dopaminergic neuron maturation.

To avoid the unwanted toxic effects of PARIS during brain development, PARIS expression was turned off during development and until 3 weeks of age by providing the mating pair and their pups with a doxycycline-supplemented diet that inactivates tTA (Fig. 1A). Repression of PARIS expression was confirmed by immunofluorescent images of ventral midbrain sections from 3-week-old PARIS Tg mice and littermate controls. No PARIS immunofluorescence signal was detected in tyrosine hydroxylase (*TH*)-positive dopamine neurons in the VM of both mouse groups (Supplementary Fig. 2A).

PARIS expression was examined by western blot analysis of the cortex, VM, and cerebellum (CB) from 2-month-old PARIS Tg mice maintained on a regular diet from 3 weeks of age, and littermate controls were maintained under the same conditions. PARIS was selectively and robustly expressed only in the ventral midbrain of PARIS Tg mice when monitored by anti-PARIS and anti-FLAG antibodies (Fig. 1B, C, D). We also observed cells with higher PARIS expression only in the ventral midbrain of PARIS Tg mice compared to that in those from littermate controls, as determined by immunohistochemistry using a PARIS-specific antibody (Fig. 1E). The cortex and hippocampus displayed comparable PARIS signals with no overall differences (Fig. 1E). Co-immunofluorescence labeling of the dopamine neuron marker TH and PARIS or FLAG revealed selective PARIS accumulation in the dopamine neurons of the ventral midbrain of PARIS Tg mice (Fig. 1F, and Supplementary Fig. 2B). There was no demonstrable exogenous transgene PARIS expression in GAD67-positive gamma-aminobutyric acid (GABA)ergic neurons and GFAP-positive astrocytes in the ventral midbrains of PARIS Tg (Supplementary Fig. 2C, D). Overall, using the conditional transgenic approach, we achieved successful dopaminergic expression of the PD disease protein PARIS, beginning at 3 weeks of age, to investigate its pathological role in mature dopaminergic neurons.

### Conditional PARIS Tg mice develop motor impairments with progressive and robust dopamine cell loss

PARIS is toxic to dopaminergic neurons when adenoassociated virus (AAV)-PARIS is delivered to the ventral midbrain or PARIS is expressed by the pan-neuronal promoter CamKIIα [8, 10]. Here, we explored the dopaminergic toxicity with conditional PARIS expression in midbrain dopaminergic neurons using a DAT-tTA driver. At the basal condition of 3 weeks of age with no PARIS induction, the PARIS Tg mice and littermate controls maintained on the doxycycline diet showed no neurodegeneration, as determined by the stereological counting of TH-positive dopamine neurons in the substantia nigra pars compacta (Supplementary Fig. 2E, F). TH-positive axon fiber densities in the striatum were also comparable, with no signs of degeneration in control or PARIS Tg mice, of which PARIS expression in dopaminergic neurons was suppressed by the doxycycline diet (Supplementary Fig. 2G, H).

**Fig. 2:**
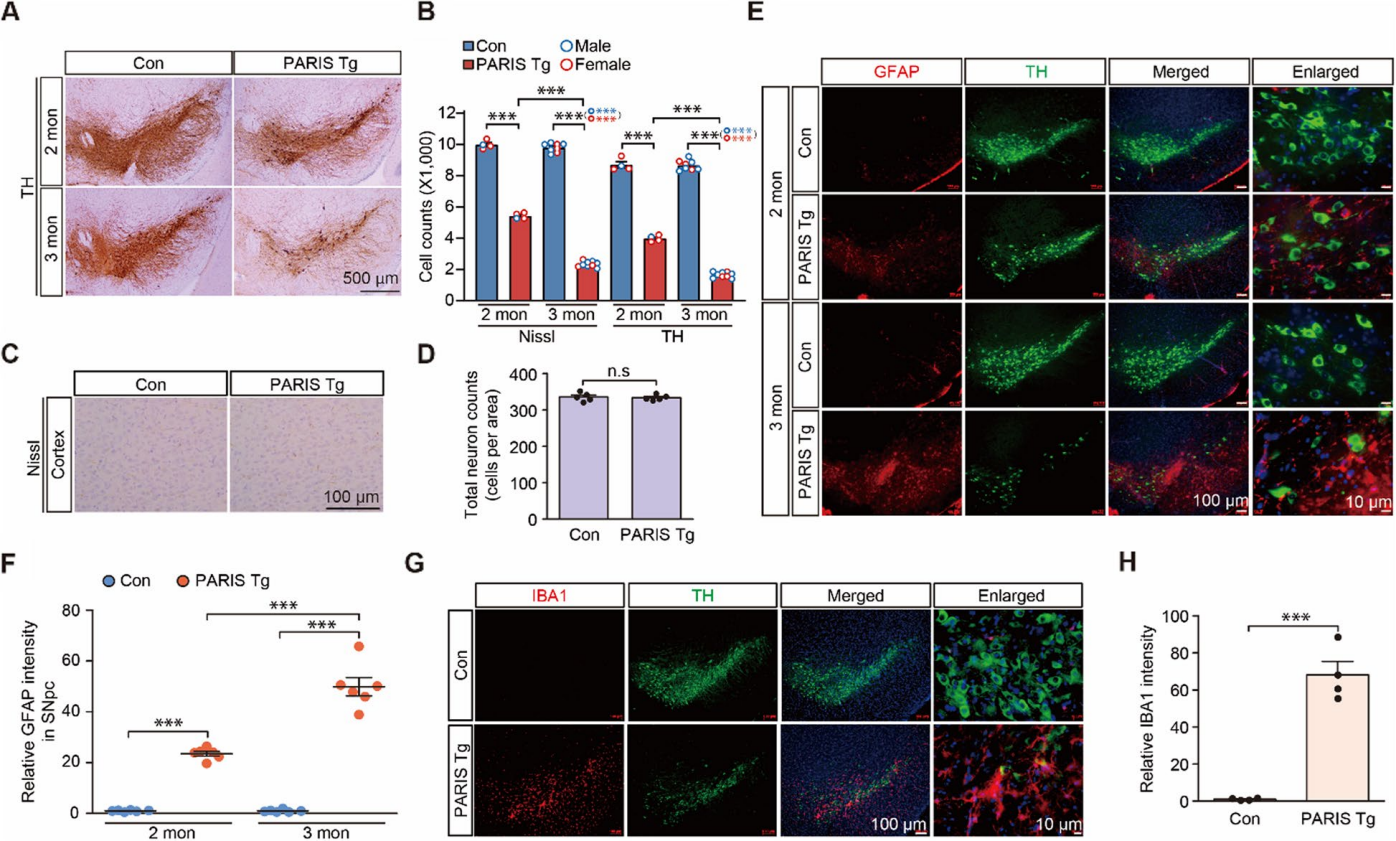
Dopaminergic PARIS expression leads to a progressive dopaminergic neuron loss and neuroinflammation. **A** Representative tyrosine hydroxylase (TH) immunohistochemical staining for ventral midbrains of 2–3-month-old control or PARIS Tg mice. Scale bar: 500 μm. **B** Stereological assessment of TH-positive and Nissl-stained dopaminergic neurons in SNpc of 2–3-month-old control or PARIS Tg mice (*n* = 4 per 2-months-old control and PARIS Tg mice, 8 per 3-month-old control, 9 per 3-month-old PARIS Tg mice). Male and female mice are indicated by blue and red empty circles, respectively. **C** Representative Nissl staining for the cortex of 3-month-old control or PARIS Tg mice. Scale bar: 100 μm. **D** Stereological assessment of total neurons in the cortex of 3-month-old control or PARIS Tg mice (*n* = 5 mice per group). **E** Representative immunofluorescence images obtained using anti-GFAP and -TH antibodies shows that GFAP-positive astrocytes increased in the SN of 2–3-month-old PARIS Tg mice compared to those in each control. Scale bar: 100 μm in all images and 10 μm in enlarged images. **F** Quantification of relative GFAP signal intensities in the SN from the indicated experimental groups in panel E (*n* = 6 mice per group). **G** Representative immunofluorescence images obtained using anti-IBA1 and -TH antibodies shows that IBA1-positive microglia increased in the SN of 3-month-old PARIS Tg mice compared to that in each control. Scale bar: 100 μm in all images and 10 μm in enlarged images. **H** Quantification of relative IBA1 signal intensities in the SN from the indicated experimental groups in panel G (*n* = 4 mice per group). Data in all panels are mean ± standard error of the mean. ***P < 0.001, unpaired two-tailed student’s *t*-test or two-way analysis of variance (ANOVA) test followed by Tukey’s post hoc analysis. n.s, nonsignificant

**Fig. 3:**
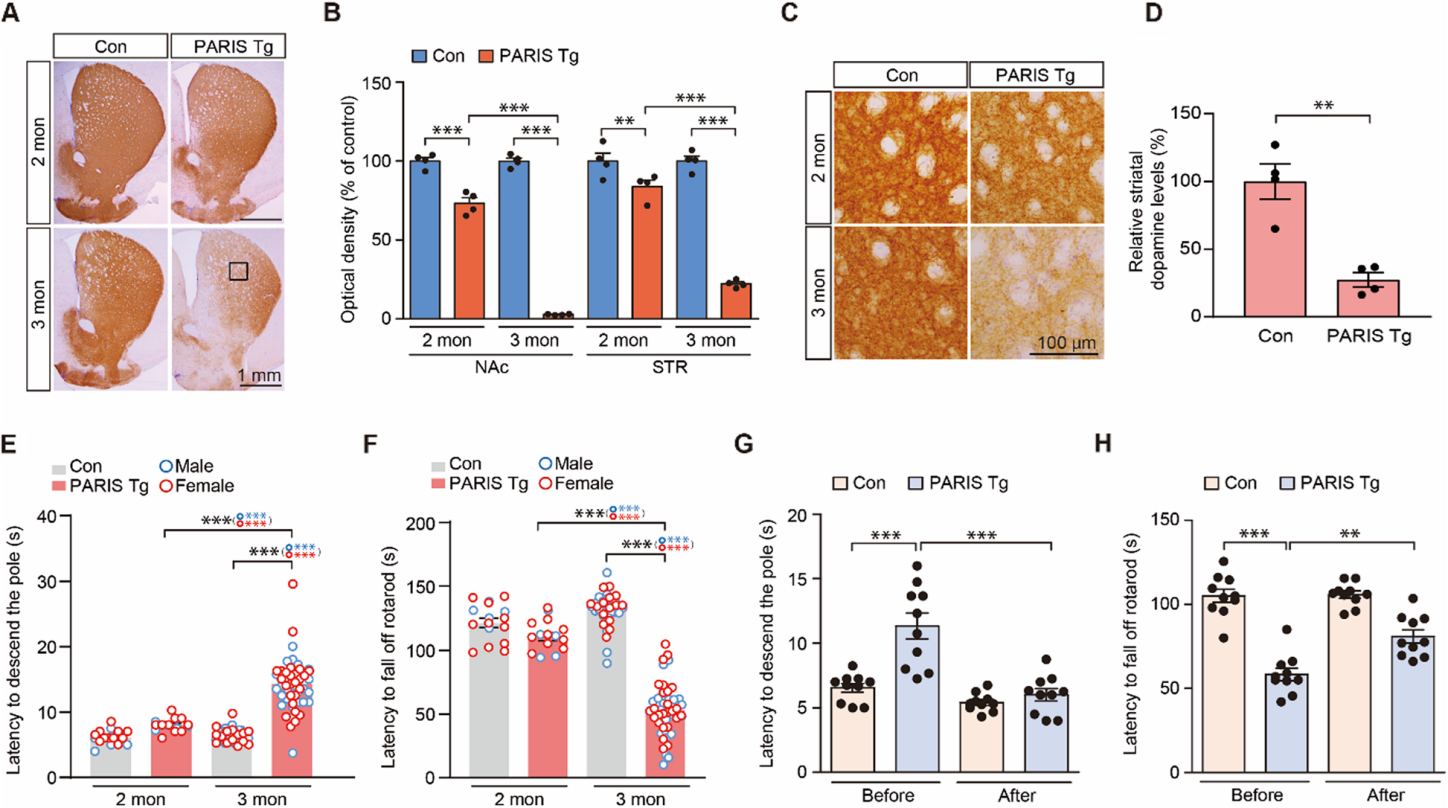
Dopaminergic PARIS expression drives progressive and nigrostriatal-specific motor impairments. **A** Representative tyrosine hydroxylase (TH) immunohistochemical staining for striatum (STR) and nucleus accumbens (NAc) of 2–3-month-old control or transgenic mice with dopamine transporter-positive feedforward tetracycline-regulatable transcription activator and human *PARIS* transgene under the control of the Tet promoter (PARIS Tg mice). Scale bar: 1 mm. **B** The graph showing the optical density of TH-positive fibers in the NAc and STR from the indicated experimental groups in panel A (*n* = 4 mice per group). **C** Magnified images of boxed regions from indicated experimental groups in panel A. Scale bar: 100 μm. **D** Striatal measurements of dopamine levels, as performed by high performance liquid chromatography, from 3-month-old control or PARIS Tg mice (*n* = 4 mice per group). **E** The pole test. The graph shows the differences of latency to descend the pole of 2–3-month-old control or PARIS Tg mice (*n* = 16 mice for 2-month-old control or PARIS Tg group, 44 mice for 3-month-old control group, 57 mice for 3-month-old PARIS Tg group). Related movies are in the Supplementary movie 1. Male and female mice are indicated by blue and red empty circles, respectively. **F** The rotarod test. The graph shows the differences of latency to fall off the rotating-rod of 2–3-month-old control or PARIS Tg mice (*n* = 16 mice for 2-month-old control or PARIS Tg group, 44 mice for 3-month-old control group, 57 mice for 3-month-old PARIS Tg group). Male and female mice are indicated by blue and red empty circles, respectively. **G**, **H** The behavior experiments performed 30 min after intraperitoneal injection of 5 mg/kg carbidopa and 20 mg/kg levodopa to 2.5-month-old control or PARIS Tg mice. The graph shows the differences of latency to descend the pole (**G**) and fall off the rotating-rod (**H**) of 2.5-month-old control or PARIS Tg mice (*n* = 10 mice per group). Related movies for figure panel G are in the Supplementary movie 3. Data in all panels are mean ± standard error of the mean. *P < 0.05, **P < 0.01, and ***P < 0.001, unpaired two-tailed student’s *t*-test or two-way analysis of variance (ANOVA) test followed by Tukey’s post hoc analysis

With the induction of dopaminergic PARIS expression by switching to a regular diet, we observed a progressive loss of SNpc dopaminergic neurons as determined by TH and Nissl stereology. The dopaminergic neurons in the PARIS Tg mice reduced by approximately 54.28 and 81.24% (assessed by TH counting) and by 45.66 and 76.33% (assessed by Nissl counting) at 2 and 3 months, respectively, compared to that in the littermate controls (Fig. 2A, B). Dopaminergic neuron loss was further corroborated by a marked reduction in anti-DAT (dopamine transporter, a marker for dopaminergic neurons) immunohistochemistry signals in the ventral midbrain sections of 3-month-old PARIS Tg mice compared to littermate controls (Supplementary Fig. 2I, J). Both male and female mice developed similar extent of dopaminergic neuronal loss in the SNpc at 3 months of age (Fig. 2B). Importantly, the dopaminergic neurons residing within the ventral tegmental area (VTA) also showed progressive 46.14 and 71.12% degeneration in PARIS Tg mice at 2 and 3 months, respectively (Supplementary Fig. 2 K). In contrast to this progressive dopaminergic neuron loss in the SNpc and VTA of PARIS Tg mice, the number of cortical Nissl-stained neurons in the cortices of control and PARIS Tg mice were similar (Fig. 2C, D). Consistent with progressive and robust neurodegeneration, anti-GFAP immunofluorescence revealed a gradual increase in astrogliosis in the SNpc, where dopaminergic neurons are highly populated, demonstrating ongoing neuroinflammation (Fig. 2E, F). Neuroinflammation was further evaluated using the microglial marker IBA1, which showed enhanced IBA1 immunofluorescence signals in the SNpc area of 3-month-old PARIS Tg mice (Fig. 2G, H). Along with dopamine cell loss and neuroinflammation in the SNpc, TH-positive axon fiber densities also progressively declined in 2- and 3-month-old PARIS Tg mice (Fig. 3A, B). TH optical density measurements were quite robust, with approximately 97.16 and 77.76% reductions in TH-positive fiber densities in the nucleus accumbens (NAc) and dorsal striatum (STR) of 3-month-old PARIS Tg mice, respectively (Fig. 3B). Actual loss of TH-positive axon terminal was further validated by high magnification imaging (Fig. 3C), and HPLC measurement demonstrated 72.66% depletion of neurotransmitter dopamine in the striatal tissue of 3-month-old PARIS Tg mice compared to that in the littermate controls (Fig. 3D).

Then, we sought to determine the potential behavioral abnormalities of the newly developed conditional PARIS Tg mice by testing various behavioral paradigms. Under basal conditions of no PARIS induction up to 3 weeks of age, normal open-field exploration was observed in both genotypes (Supplementary Fig. 3A–E). However, when PARIS was expressed in midbrain dopaminergic neurons, the 3-month-old PARIS Tg mice developed a slightly increased total distance and 2- and 3-month-old PARIS Tg mice showed anxiety, as shown by the increased percentage of time spent in the border zone relative to that spent at the central areas of the open field arena (Supplementary Fig. 3F–H). Both male and female PARIS Tg mice showed similar extent of anxiety phenotype in the open field test (Supplementary Fig. 3G). Consistent with the loss of the neurotransmitter dopamine and robust loss of TH-positive axon terminals at 3 months of age, PARIS Tg mice displayed a marked increase in descending time in the pole test, indicating bradykinesia and motor coordination loss, as determined by the accelerating rotarod assay (Fig. 3E, F, and Supplementary movie 1). Both male and female PARIS Tg mice exhibited similar extent of motor impairment at 3 months of age (Fig. 3E, F). Mild trend toward bradykinesia was observed in 2-month-old PARIS Tg mice, and no deficits were observed in the rotarod test (Fig. 3E, F). Nevertheless, the beam walking test identified a significant prolongation of crossing time on the narrow beam and a concomitant increase in the frequency of foot slips in 2-month-old PARIS Tg mice (Supplementary Fig. 3I, J, and Supplementary movie 2). Next, we examined whether this motor impairment was dependent on dopamine depletion in the nigrostriatal system. Therefore, we administered the dopamine precursor L-DOPA to PARIS Tg mice and littermate controls since L-DOPA treatment is widely used as an antisymptomatic therapy for patients with PD to supplement reduced dopamine transmission [23]. L-DOPA administration restored the motor function of PARIS Tg mice in the pole test by 84.8% and recovered motor coordination by 47.1% (Fig. 3G, H, and Supplementary movie 3). However, it did not rescue anxiety phenotypes in PARIS Tg mice (Supplementary Fig. 3 K, L). Collectively, PARIS Tg mice recapitulate the robust dopaminergic neurons loss that are comparable to those in patients with PD, cardinal motor symptoms, and accompanying neuroinflammation. Motor deficits in PARIS Tg mice were specific to dopamine depletion, and PARIS Tg mice did not develop any short-term memory impairments, as assessed using the Y-maze (Supplementary Fig. 3 M, N).

To investigate whether PARIS induction in adulthood could recapitulate key PD pathologies, transgene expression was initiated in dopaminergic neurons at 2.5 months of age and maintained for 2 months, until 4.5 months of age. Similar to the findings observed with PARIS induction at 3 weeks of age, adult PARIS Tg mice exhibited significant dopaminergic neuron loss in both the SNpc and VTA of the midbrain (Supplementary Fig. 4A, B). This neurodegeneration was accompanied by pronounced motor impairments (Supplementary Fig. 4C, D). Furthermore, adult PARIS Tg mice displayed a trend towards increased immobility time in the tail suspension test (Supplementary Fig. 4E). These findings demonstrate that PARIS-mediated dopaminergic neurotoxicity can occur across different ages, including adolescence and young adulthood, recapitulating key features of Parkinson's Disease pathology.

### Dopaminergic PARIS expression represses PGC-1α

**Fig. 4:**
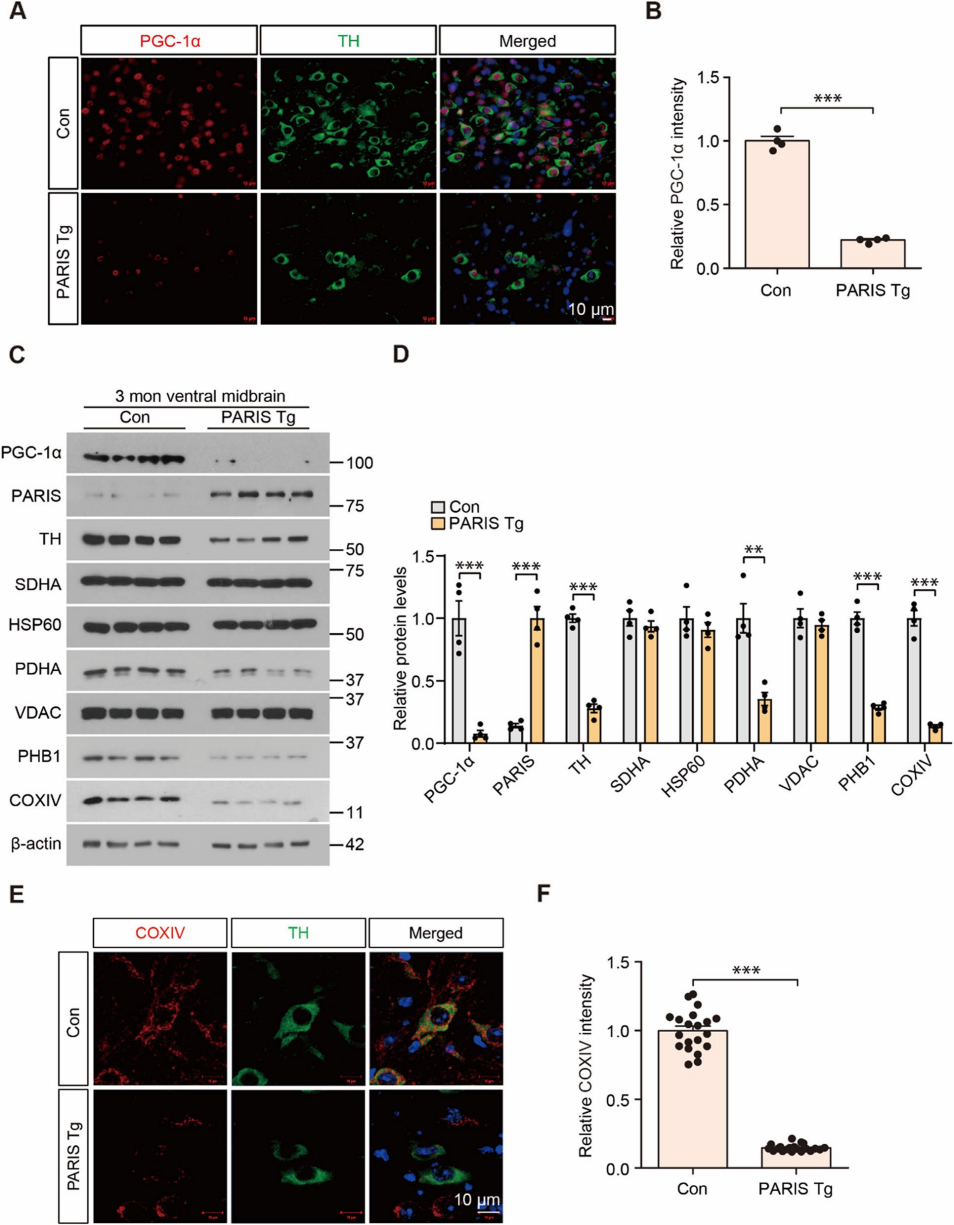
Dopaminergic PARIS expression leads to PGC-1α repression, and abnormal mitochondrial protein expression. **A** Representative immunofluorescence images obtained using anti-PGC-1α and tyrosine hydroxylase (TH) antibodies in the SN of 3-month-old PARIS Tg mice compared to that in each control. Scale bar: 10 μm. **B** Quantification of relative PGC-1α signal intensities in the SN from the indicated experimental groups in panel A (*n* = 4 mice per group). **C** Representative western blots showing PGC-1α, PARIS, TH, and several mitochondrial protein (succinate dehydrogenase complex flavoprotein subunit A [SDHA], heat shock protein 60 [HSP60], pyruvate dehydrogenase [PDHA], voltage-dependent anion channels [VDAC], prohibitin 1 [PHB1], and cytochrome c oxidase subunit IV [COXIV]) levels in the ventral midbrain of 3-month-old control or PARIS Tg mice. β-actin was used as an internal loading control. **D** Quantification analysis of relative PGC-1α, PARIS, TH, SDHA, HSP60, PDHA, VDAC, PHB1, and COXIV expression levels (normalized to those β-actin) in the experimental groups in panel C (*n* = 4 mice per group). **E** Representative immunofluorescence images obtained using anti-COXIV and -TH antibodies in 3-month-old SN of PARIS Tg mice compared to that in each control. Scale bar: 10 μm. **F** Quantification of relative COXIV signal intensities in the SN from the indicated experimental groups in panel E (*n* = 20 cells per group). Data in all panels are mean ± standard error of the mean. *P < 0.05, **P < 0.01, and ***P < 0.001, unpaired two-tailed student’s *t*-test

PARIS represses *PGC-1a*, which is a master regulator of mitochondrial biogenesis and critical for PARIS-induced dopaminergic neurodegeneration and mitochondrial deficits [10]. Therefore, we sought to monitor whether *PGC-1a* repression and mitochondrial impairment are associated with dopaminergic toxicity and neuroinflammation in PARIS Tg mice. Immunofluorescence imaging clearly showed that PGC-1α expression in the survived dopaminergic neurons in the ventral midbrain of 3-month-old PARIS Tg mice is markedly reduced compared to that in age-matched control mice (Fig. 4A, B). Robust repression of PGC-1α expression in the ventral midbrains of 3-month-old PARIS Tg was further confirmed by Western blot (Fig. 4C, D). We monitored several key mitochondrial marker proteins (succinate dehydrogenase complex flavoprotein subunit A [SDHA], heat shock protein 60 [HSP60], pyruvate dehydrogenase E1 subunit alpha 1 [PDHA], voltage-dependent anion channel 1 [VDAC], prohibitin 1 [PHB1], and cytochrome c oxidase subunit IV [COXIV]) in ventral midbrain tissues from 3-month-old PARIS Tg mice and age-matched controls using western blotting. Among others, PDHA, PHB1, and COXIV levels in the PARIS Tg mouse VM were reduced by > 50% compared to those in the littermate controls (Fig. 4C, D). TH expression was also markedly reduced, indicating ongoing dopaminergic neuron degeneration with PARIS accumulation (Fig. 4C, D). SDHA, HSP60, and VDAC expression did not change in the VM of PARIS Tg mice (Fig. 4C, D). Consistent with the fact that PGC-1α is a master regulator of mitochondrial biogenesis, the signal intensities of COXIV, which labels the mitochondria, in TH-stained dopaminergic neurons in the ventral midbrain of PARIS Tg mice robustly decreased compared to those in age-matched controls (Fig. 4E, F).

To examine the expression of PGC-1α and selected mitochondrial marker proteins in the early stages of neurodegeneration, Western blot analysis was performed on ventral midbrain tissues from 2-month-old PARIS Tg and littermate control mice. At this early stage with about 50% dopaminergic neurodegeneration, a marked reduction in PGC-1α protein levels was observed in PARIS Tg mice, concurrent with robust PARIS accumulation (Supplementary Fig. 4F, G). Interestingly, while PDHA and PHB1 protein levels remained unaltered, COXIV expression was significantly decreased in 2-month-old PARIS Tg mice (Supplementary Fig. 4F, G). These findings suggest that PGC-1α downregulation and mitochondrial dysfunction, as evidenced by reduced COXIV expression, occur relatively early in the pathogenesis of PARIS-mediated neurodegeneration.

### Pharmacological c-Abl inhibition prevents PD-related pathologic feature development in PARIS Tg mice

**Fig. 5:**
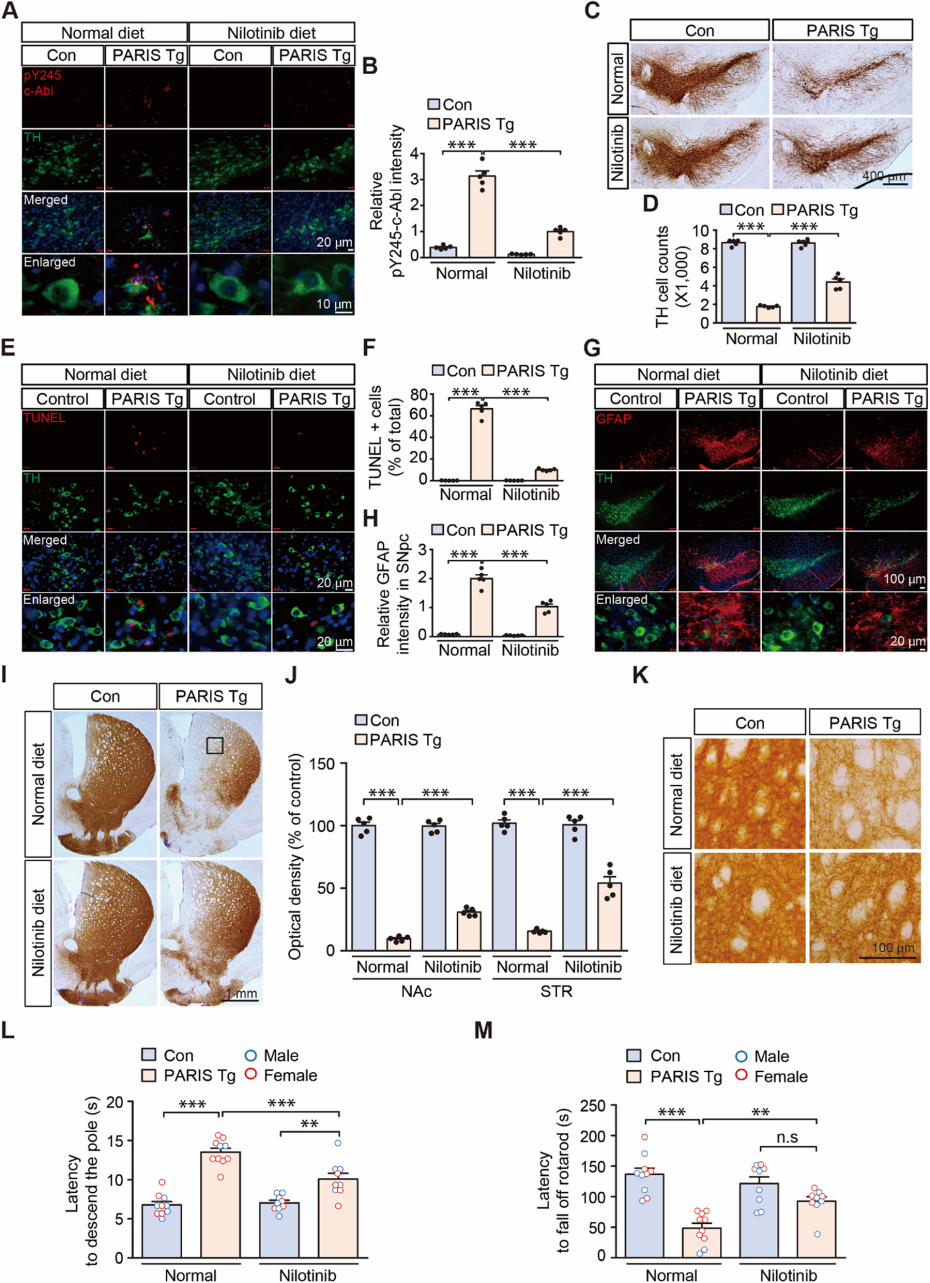
Pharmacological c-Abl inhibition mitigates nigral pathologies and motor impairments in PARIS Tg mice. **A** Representative pY245-c-Abl and tyrosine hydroxylase (TH) immunofluorescence of the substantia nigra of control or PARIS Tg mice brain with or without nilotinib. Scale bar: 20 μm in all images and 10 μm in enlarged images. **B** Quantification of relative pY245-c-Abl signal intensities in the SN from the indicated experimental groups in panel A (*n* = 5 mice per group). **C** Representative TH immunohistochemical staining of the ventral midbrains of 3-month-old control or PARIS Tg mice with or without nilotinib. Scale bar: 400 μm. **D** Stereological assessment of TH-positive dopaminergic neurons in the SNpc of control or PARIS Tg mice with or without nilotinib (*n* = 5 mice per group). **E** Representative images of TUNEL assay for substantia nigra of control or PARIS Tg mice with or without nilotinib treatment. Scale bar: 20 μm. **F** Quantification of the percentage of TUNEL-positive cells in TH-positive neurons from the indicated experimental groups in panel E (*n* = 5 mice per group). **G** Representative immunofluorescence images obtained using anti-GFAP and -TH antibodies in the SN of 3-month-old control or PARIS Tg mice with or without nilotinib. Scale bar: 100 μm in all images and 20 μm in enlarged images. **H** Quantification of relative GFAP signal intensities in the SN from the indicated experimental groups in panel G (*n* = 5 mice per group). **I** Representative TH immunohistochemical staining of nucleus accumbens (NAc) and striatum (STR) of 3-month-old control or PARIS Tg mice with or without nilotinib. Scale bar: 1 mm. **J** The graph showing the optical density of TH-positive fibers in the NAc and STR of each group of panel I (*n* = 5 mice per group). **K** Magnified images of boxed regions from indicated experimental groups in panel I. **L** The pole test of control or PARIS Tg mice with or without nilotinib treatment (*n* = 10 mice per normal diet groups and *n* = 9 mice per nilotinib diet groups). Related movies are in the Supplementary movie 4. Male and female mice are indicated by blue and red empty circles, respectively. **M** The rotarod test of control or PARIS Tg mice with or without nilotinib treatment (*n* = 10 mice per normal diet groups and *n* = 9 mice per nilotinib diet groups). Male and female mice are indicated by blue and red empty circles, respectively. Data in all panels are mean ± standard error of the mean. **P < 0.01 and ***P < 0.001, two-way analysis of variance (ANOVA) test followed by Tukey’s post hoc analysis. n.s, nonsignificant

We examined c-Abl activation, which has been reported to be a pathological molecular hallmark of PD and could potentially be induced by PARIS expression and mitochondrial impairment [12, 16, 17]. pY245-c-Abl expression increased in the TH-positive dopaminergic neurons of the VM of 3-month-old PARIS Tg mice, indicating c-Abl overactivation (Fig. 5A, B). Western blot analysis of ventral midbrain tissue from 2- and 3-month-old PARIS Tg mice further confirmed the presence of c-Abl activation and c-Abl-dependent phosphorylation of PARIS at tyrosine 137 (pY137-PARIS) (Supplementary Fig. 4F, G, and Supplementary Fig. 5A, B). To determine whether c-Abl activation is responsible for PARIS-induced PD relevant phenotypes, PARIS Tg mice were fed a diet containing the c-Abl inhibitor nilotinib starting at 3 weeks of age when PARIS induction was initiated (Supplementary Fig. 5C). For PARIS Tg mice, dopaminergic PARIS induction was continued until 3 months of age with or without a nilotinib diet for subsequent behavioral testing and pathological analysis (Supplementary Fig. 5C).

Nilotinib administration through the diet of PARIS Tg mice, without affecting PARIS transgene expression (Supplementary Fig. 5D), effectively suppressed c-Abl activity, as evidenced by a marked reduction in pY245-c-Abl expression in TH-positive dopaminergic neurons (Fig. 5A, B). Nilotinib treatment successfully inhibited PARIS-induced c-Abl activation and subsequent phosphorylation of PARIS at tyrosine 137 (pY137-PARIS) (Supplementary Fig. 5A, B). Importantly, nilotinib treatment partially rescued the robust reduction in TH expression observed in the ventral midbrains of 3-month-old PARIS Tg mice (Supplementary Fig. 5A, B). This partial rescue likely correlates with a slightly higher level of PARIS protein accumulation (Supplementary Fig. 5A, B), potentially due to the increased survival of PARIS-expressing dopaminergic neurons in nilotinib-treated mice. Furthermore, nilotinib treatment effectively modulated PARIS-mediated downstream signaling pathways [8, 10–12, 24]. Western blot analysis revealed that nilotinib restored MDM4 expression, and inhibited p53 phosphorylation, which were dysregulated in PARIS Tg mice (Supplementary Fig. 5A, B). Similarly, nilotinib treatment prevented the PARIS-induced downregulation of PGC-1α and mitochondrial proteins, including PDHA, PHB1, and COXIV (Supplementary Fig. 5A, B). These findings demonstrate that c-Abl inhibition by nilotinib effectively attenuates PARIS-mediated neurotoxicity potentially by suppressing key downstream signaling pathways involved in dopamine neuron death and mitochondrial dysfunction.

The inhibition of c-Abl activity in dopaminergic neurons prevented dopaminergic neuron loss by 39% in the substantia nigra pars compacta of PARIS Tg mice (Fig. 5C, D). Consistent with the prevention of dopaminergic cell loss, ongoing TH neurodegeneration in PARIS Tg mouse VM was markedly reduced by nilotinib treatment, as monitored by TUNEL analysis (Fig. 5E, F). Neuroinflammation was also examined by GFAP immunofluorescence, which showed an approximate 48% reduction in GFAP infiltration into substantia nigra pars compacta in the PARIS Tg mice with nilotinib treatment (Fig. 5G, H). Along with the neuroprotection and neuroinflammation suppression in PARIS Tg mice by nilotinib mediated c-Abl inhibition, nilotinib administration prevented TH axon terminal loss in PARIS Tg mice by 24% and 46% in the NAc and dorsal STR, respectively (Fig. 5I–K).

In the open-field test, PARIS Tg mice displayed trends of reduced total exploratory distance, which was not affected by nilotinib (Supplementary Fig. 5E, F). While PARIS Tg mice developed anxiety phenotypes with 25% increased time spent in the border area, nilotinib administration alone in control mice induced a trend of increased anxiety (Supplementary Fig. 5E, G). Consistent with the preservation of the nigrostriatal dopaminergic circuit by nilotinib treatment in PARIS Tg mice, bradykinesia phenotypes in PARIS Tg mice were substantially reversed by 54% after nilotinib treatment (Fig. 5L, and Supplementary movie 4). Motor coordination was also normalized to an extent comparable to that in normal control mice after treating the PARIS Tg mice with nilotinib (Fig. 5M). Overall, our preclinical study on PARIS Tg mice demonstrated that c-Abl activation largely mediates dopaminergic neurodegeneration and neuroinflammation, contributing to motor impairment in PARIS Tg mice.

### Transcriptomic analysis of differentially expressed genes (DEGs) in PARIS Tg mouse midbrain reveals potential functional pathways in PD pathogenesis

**Fig. 6:**
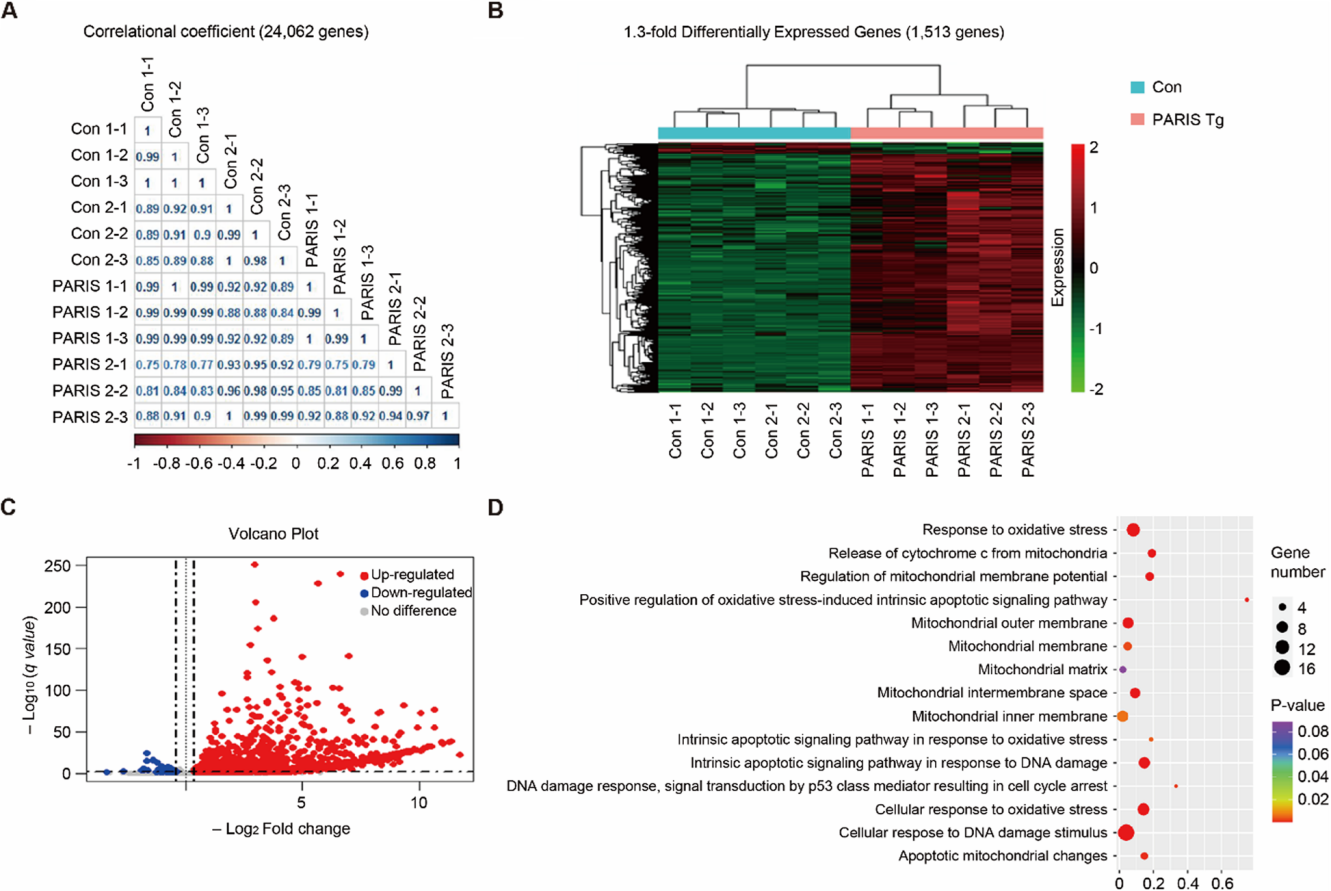
PARIS-driven transcriptomic alterations in the midbrains of PARIS Tg mice. **A** Correlational coefficient gene expression between control and PARIS Tg mouse midbrains. **B** Heat map of 1.3-fold differentially expressed genes (DEGs). A total of 1,513 DEGs between control and PARIS Tg mouse. **C** Volcano plot of 1.3-fold upregulated or downregulated DEGs in PARIS Tg mouse midbrains (*q* value < 0.05). **D** Gene ontology (GO) analysis of DEGs for mitochondrial dysfunction-associated genes

To analyze the potent transcriptomic regulation of PD pathogenesis, we performed RNA-seq to facilitate unbiased transcriptomic profiling of gene expression differences in the midbrains of PARIS Tg mice. The comprehensive relationship between each feature of the control and PARIS Tg mouse models was computed using the Pearson correlation coefficient for the dataset of 24,062 genes (Fig. 6A). The overall number of DEGs that showed a 1.3-fold increase was 1,513 genes (Fig. 6B) and 1,448 genes were upregulated, while 65 genes were downregulated in PARIS Tg mouse midbrain (Fig. 6C). TH was significantly downregulated (log_2_FC = − 1.388). Functional enrichment was determined using gene ontology (GO) categorization analysis of gene sets annotated to canonical pathways. Each bubble plot reflects the biological processes and cellular components with the number of associated DEGs corresponding to bubble size (Fig. 6D). The ranked GO terms were related to oxidative stress and mitochondria, and details are presented in Supplementary Table 1. Furthermore, we also filtered and analyzed the DEGs with more than twofold alterations in PARIS Tg mice (Supplementary Fig. 6, and Supplementary Table 2). These DEGs with great fold changes (more than 4 or twofold alteration) are enriched in several GO pathways that are implicated in cholesterol dehydrogenase activity, bile acid and bile salt transport, abiotic stimulus detection, and meiosis (Supplementary Fig. 7). To investigate the functional consequences of altered GO pathways in PARIS Tg brains, we analyzed lipid metabolites in striatal tissues using liquid chromatography-mass spectrometry (LC–MS). Consistent with the observed reduction in bile acid transporters (*Slc10a7, Slc10a3*, and *Slc10a4*), we found significantly decreased levels of ursodeoxycholic acid (UDCA) in the brains of PARIS Tg mice compared to controls (Supplementary Fig. 7D). Furthermore, potentially associated with altered cholesterol dehydrogenase activity, we observed a reduction in pregnenolone levels in the striatum of PARIS Tg mice, while cholesterol and progesterone levels remained unchanged (Supplementary Fig. 7E, F, G). This agnostic analysis, along with the functional validation of select GO pathways, provides transcriptome-wide support for the potential functional associations of the PARIS Tg mouse model with PD pathologies.

### Single nucleus RNA sequencing (snRNA-seq) analysis identified cell types and transcriptional heterogeneity in PARIS Tg mouse midbrain

**Fig. 7:**
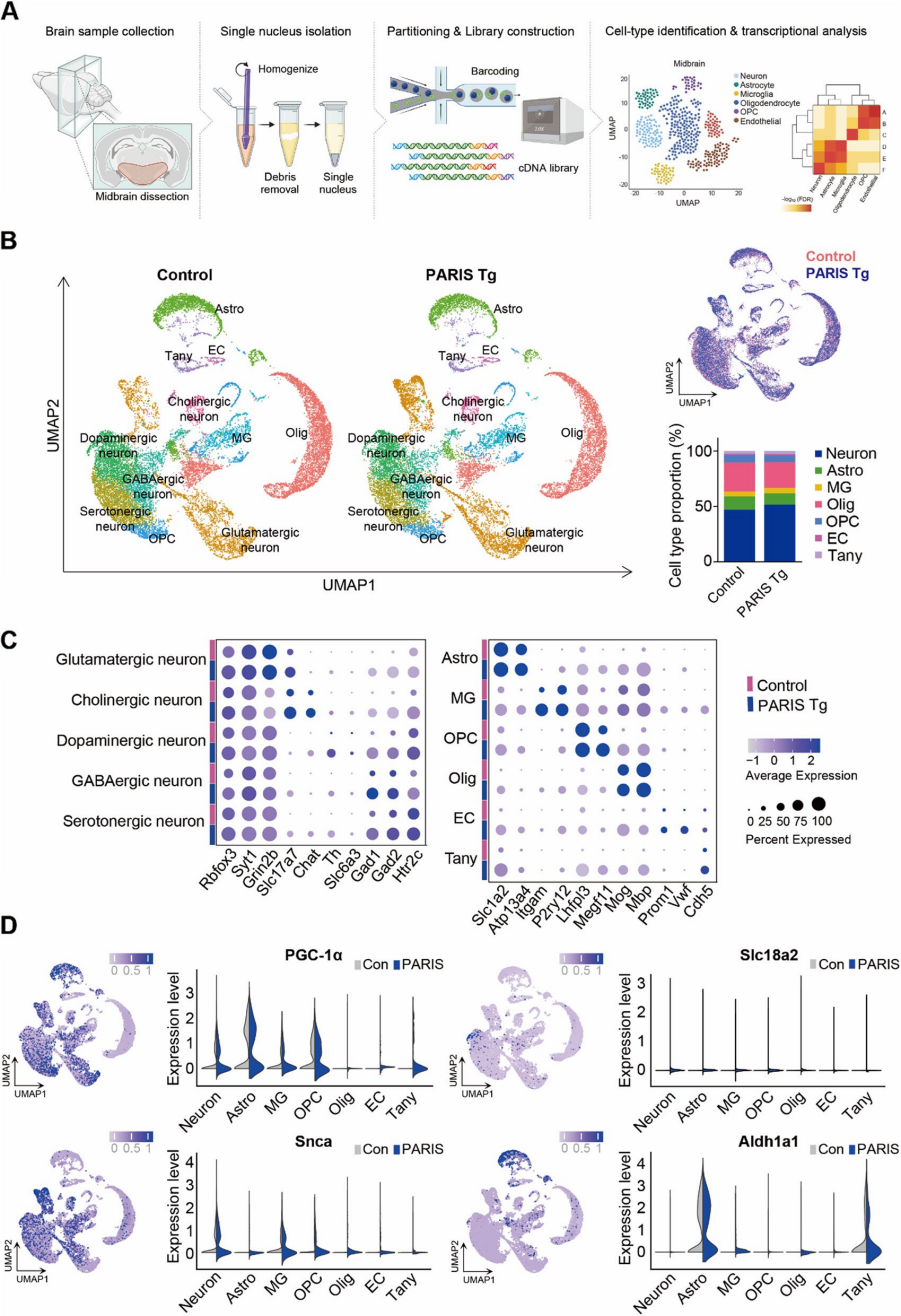
snRNA-seq distinguishes cell clusters and profiles transcriptional regulation in PARIS Tg mouse model. **A** Schematic overview of snRNA-seq design from midbrain in PARIS Tg. Illustration was created in BioRender. Kim, S. (2025) https://BioRender.com/r40y086. **B** UMAP plot clustering 11 distinguished clusters and showing major 11 cell-types; neuron, astrocyte (Astro), microglia (MG), oligodendrocyte precursor cell (OPC), oligodendrocyte (Olig), endothelial cell (EC), tanycyte (Tany). Overlapped whole cell distribution of control and PARIS Tg, and the percentage of distinct major cell types reflect the cell proportion in control and PARIS Tg. **C** Dot plot showing the selected marker expressions to identify cell types across both control and PARIS Tg. **D** PD-associated gene expression patterns in each cell cluster on UMAP projections of PARIS Tg. Violin plots showing gene expression levels in each cell type

To investigate the transcriptomic diversities of different cell types in PARIS Tg, we generated single nucleus transcriptomes of midbrain from control and PARIS Tg by nucleus isolation using 10X Genomics (Fig. 7A). Total 21,780 and 19,688 nuclei of control and PARIS Tg were used for analysis; we retained gene expression profiles in median 2,571 and 3,057 genes per nucleus each. We adopted Uniform Manifold Approximation and Projection (UMAP) to visualize the distinguished cell types in clusters, by which 11 distinct cell types were projected. Overlapping whole cell distribution was depicted across all clusters (Fig. 7B, and Supplementary Fig. 8A). The proportion of each cell type was calculated for both control and PARIS Tg midbrain sample. Neurons constituted the largest cell population, representing 47.0% and 51.7% in control and PARIS Tg, respectively. This was followed by oligodendrocytes (Olig) at 26.3% and 23.4%, astrocytes (Astro) at 12.1% and 10.1%, oligodendrocyte precursor cells (OPCs) at 6.8% and 6.1%, microglia (MG) at 4.4% and 4.9%, tanycytes (Tany) at 2.5% and 2.8%, and endothelial cells (EC) at 1.0% and 1.1%, respectively (Fig. 7B, Table S3). Differential gene expression analysis identified distinct clusters of cells, highlighting neuronal and non-neuronal cell types. Neurons expressing *Rbfox3* and *Syt1* includes glutamatergic (*Grin2b*, *Slc17a7*), cholinergic (*Slc17a7*, *Chat*), dopaminergic (*Th*, *Slc6a3*), GABAergic (*Gad1*, *Gad2*), and serotonergic (*Htr2c*). Non-neuron clusters include astrocytes (*Slc1a2*, *Atp13a4*), microglia (*Itgam*, *P2ry12*), OPC (*Lhfpl3*, *Megf11*), oligodendrocytes (*Mog*, *Mbp*), endothelial cells (*Prom1*, *Vwf*), and tanycyte (*Cdh5*) (Fig. 7C). As expected, the expression level of *PGC-1α* in the neuron cluster was downregulated (average log2FC = −0.4765) due to the PARIS-induced *PGC-1α* suppressive mechanism. For other genes that are deemed as causative factors in PD, their overall expression levels in neuron were upregulated for *Snca* (average log2FC = 0.5373), and downregulated for *Slc18a2* (average log2FC = −0.2616) and *Aldh1a1* (average log2FC = −0.2164) (Fig. 7D, Supplementary Fig. 8B, C, Table S4).

**Fig. 8:**
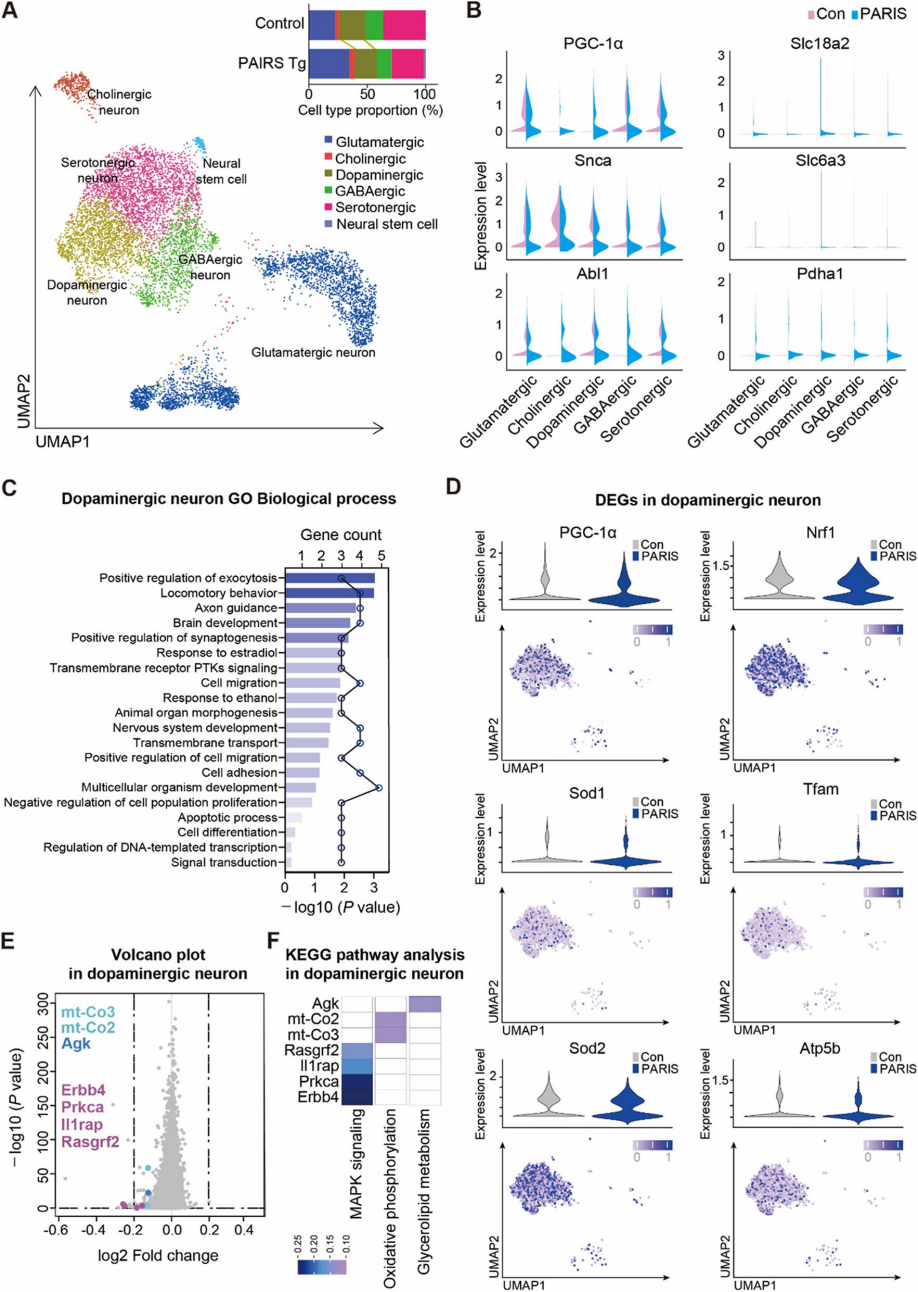
Subpopulations of neurons in PARIS Tg midbrains and transcriptional regulation in dopaminergic neurons. **A** UMAP visualization of re-clustered neuron subpopulations identified in Fig. 7B, and the proportion of neuron subpopulations in the ventral midbrains that are clustered into 6 distinguished types. **B** Selected PD-associated gene expressions in each neuron type of control and PARIS Tg indicated by violin plots. **C** Biological process in GO terms enriched with DEGs (filtration using log2FC < −0.15, and -log10 (P value) > 0.2) in dopaminergic neurons. Bar graph indicates GO terms, and dot graph indicates included gene counts. **D** Violin plots depicting the PGC-1α and its target gene expression levels (y axis) in dopaminergic neurons. UMAP showing the corresponding gene expression pattern in dopamine cell cluster of PARIS Tg. **E** A volcano plot depicting the genes downregulated in dopaminergic neurons of PARIS Tg as compared to control. **F** KEGG pathway analysis of selected DEGs in dopaminergic neurons of PARIS Tg

To investigate the neuronal subpopulation that is influenced in PARIS Tg mice, we re-clustered neuron population into 6 distinct subtypes then identified as glutamatergic, cholinergic, dopaminergic, GABAergic, serotonergic neurons and neural stem cell. The proportion of neuronal cells showed that dopaminergic neurons were slightly depleted in PARIS Tg (19.36%) compared to control (23.06%) (Fig. 8A, Supplementary Fig. 9A, B). The PD-related genes including *PGC-1α*, *Slc18a2*, *Snca*, and *Slc6a3* were observed for their expression level in each neuron subpopulation. We also observed PD pathogenesis-associated genes, such as *Abl1* and *Pdha1* (Fig. 8B, Table S5). Previous studies have shown that the neurodegenerative progression in PD is defined by dopaminergic neuronal loss [25]. Therefore, we analyzed the biological processes through GO terms, particularly in dopaminergic neurons, which showed DEGs enrichment in several biological processes including positive regulation of exocytosis, and synaptogenesis, axon guidance, apoptotic process, and regulation of DNA-template transcription (Fig. 8C, Tables S6 and S7). PARIS conferred downregulation of *PGC-1α*; consequently, PGC-1α downstream genes, including *Nrf1, Sod1, Sod2, Tfam*, and *Atp5b*, were also affected in PARIS Tg as compared to control (Fig. 8D, Supplementary Fig. 10A). Additionally, DEGs in dopaminergic neurons, which are potentially involved in mitochondria-related multiple cascades (PGC-1α regulated pathways, mTOR pathways, p53-dependent cell death), include *Calb1, Sox6, Ucp3, Ucp2, Rptor, Yy1*, and *Mdm4* (Supplementary Fig. 10B, C). Pathway analysis of dopaminergic neuron DEGs between control and PARIS Tg groups identified key processes implicated in PD pathology. Downregulated genes such as *Rasgrf2, Il1rap, Prkca,* and *Erbb4* were linked to the MAPK signaling pathway, which plays a dual role in cellular processes like survival, apoptosis, and oxidative stress [26–28]. Additionally, downregulated *mt-Co2* and *mt-Co3* were implicated in oxidative phosphorylation, while *Agk* (acylglycerol kinase), involved in glycerolipid metabolism, was also downregulated (Fig. 8E, F, Table S8). These findings suggest that PARIS-mediated dysregulation of gene expression may exacerbate pathological vulnerabilities in midbrain dopaminergic neurons in PD.

**Fig. 9:**
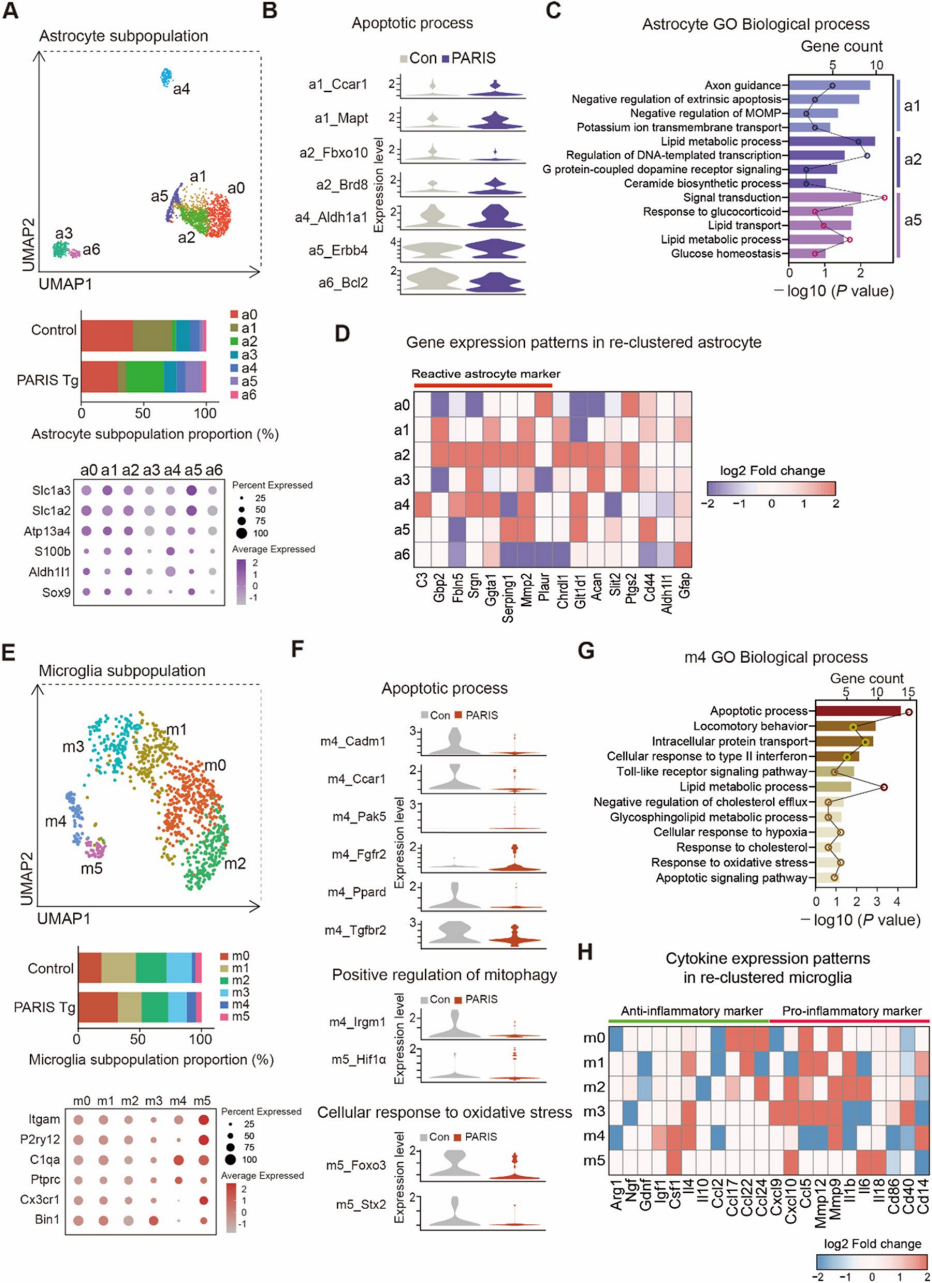
Subpopulations and transcriptional regulation of astrocyte and microglia in PARIS Tg midbrains. **A** UMAP visualization showing re-clustered astrocyte subpopulations in the ventral midbrains identified in Fig. 7B, and the proportion of astrocyte subpopulations that clustered into 7 distinguished types. Dot plot shows the expressions of selected astrocyte markers in PARIS Tg. **B** Violin plots showing scaled expression levels of the genes that are related to apoptotic process from distinct astrocyte subclusters. **C** Biological process in GO terms enriched with DEGs (a1, a2 log2FC >|0.4|, a5 log2FC >|0.55|) in re-clustered astrocyte. Bar graph indicates GO terms, and dot graph indicates included gene counts. The full list of DEG enriched GO biological processes in astrocytes is reported in Supplementary Table 10. **D** Gene expression patterns of known astrocyte activation markers in re-clustered astrocyte subpopulations that depict the genes up- or downregulated in PARIS Tg compared to control. **E** UMAP visualization showing re-clustered microglia subpopulations in the ventral midbrains identified in Fig. 7B, and the proportion of microglia subpopulations that clustered into 6 distinguished types. Dot plot shows the expressions of selected microglia markers in PARIS Tg. **F** Violin plots showing scaled expression levels of genes that are related to apoptotic process, positive regulation of mitophagy, and cellular response to oxidative stress from distinct microglia subclusters. **G** Biological process in GO terms enriched with DEGs (m4 log2FC >|0.95|) in re-clustered microglia. Bar graph indicates GO terms, and dot graph indicates included gene counts. The full list of DEG enriched GO biological processes in microglia is reported in Supplementary Table 12. **H** Expression patterns of cytokines in re-clustered microglia subpopulations that depict the genes up- or downregulated in PARIS Tg compared to control

To investigate the potential non-cell-autonomous mechanisms underlying dopaminergic neuronal phenotypes and additional PD-linked pathologies by dopaminergic PARIS expression in our PD model mouse, we analyzed astrocytes and microglia. Re-clustering of astrocytes identified seven subpopulations (a0–a6) defined by distinct marker expression profiles, with each subpopulation showing unique proportions (Fig. 9A, Supplementary Fig. 11A). Differential gene expression analysis revealed that genes involved in apoptotic processes were dysregulated across astrocyte subpopulations. Notably, *Ccar1, Mapt, Brd8, Aldh1a1*, and *Erbb4* were upregulated, while Fbxo10 and Bcl2 were downregulated in specific subpopulations (Fig. 9B, Table S9). Additional transcriptional alterations induced by dopaminergic PARIS expression were identified across astrocyte subpopulations (Supplementary Fig. 11B). GO analysis highlighted key biological processes, including negative regulation of extrinsic apoptosis (*Acvr1, Nrp1, Scg2*), negative regulation of mitochondrial outer membrane permeabilization (MOMP) in apoptotic signaling (*Mpv17l, Slc25a4*), and potassium ion transmembrane transport (*Dpp6, Kcnip3, Slc1a3*) in subpopulation a1. Subpopulation a2 showed enrichment in the ceramide biosynthetic process (*Samd8, Agk*), while a5 was associated with response to glucocorticoids (*Acsbg1, A2m, Ptgds*), lipid transport (*Vmp1, Osbpl6, Pitpnc1, Atg2b*), lipid metabolic processes (*Plce1, Acsbg1, Chpt1, Ptgds, Gpld1, Mgll, St6galnac5*), and glucose homeostasis (*Ptch1, Adcy8, Csmd1*) (Fig. 9C, Table S10). Astrocyte reactivity is a known pathological feature of PD linked to neuroinflammation [29, 30]. Notably, subpopulation a2, characterized by neurotoxic astrocytic signatures, was markedly increased in PARIS Tg (29.7%) compared to controls (3.04%) (Fig. 9A, D, Supplementary Fig. 11C, Table S3).

Re-clustering of microglia revealed six subpopulations (m0–m5) with distinct marker expression profiles and varying proportions (Fig. 9E, Supplementary Fig. 12A). Differential gene expression analysis showed dysregulation of genes involved in apoptotic processes (*Cadm1, Ccar1, Pak5, Fgfr2, Ppard, Tgfbr2*), positive regulation of mitophagy (*Irgm1, Hif1a*), and cellular response to oxidative stress (*Foxo3, Stx2*) (Fig. 9F, Table S11). Additional transcriptional changes induced by dopamine neuron-expressed PARIS were observed across microglia subpopulations (Supplementary Fig. 12B). GO analysis further identified associations with lipid metabolic processes, cholesterol efflux, glycosphingolipid metabolism, and response to cholesterol (Fig. 9G, Table S12). Microglia are known to contribute to PD susceptibility through innate immune system functions [31]. Here, cytokine-related gene expression patterns were distinct across microglia subpopulations. Interestingly, the proportion of the anti-inflammatory marker-expressing subpopulation m4 was increased, while pro-inflammatory marker-expressing subpopulations were reduced (Fig. 9H, Supplementary Fig. 12C).

## Discussion

### Generation of PD mouse model with robust clinical-level pathologies

In this study, we engineered a novel PD mouse model that recapitulates the key pathological features of clinical PD. Progressive and robust dopaminergic neurodegeneration defines cardinal motor deficits in clinical PD and a useful PD mouse model should recapitulate selective and > 70% loss of dopaminergic neurons in a manageable time window for preclinical evaluation [3, 4]. Traditional toxin-based PD mouse models (such as MPTP intoxication and 6-OHDA stereotaxic injection models) faithfully produce near complete loss of nigral dopaminergic neurons, however acute dopaminergic cell death occurs within several days [4, 32]; thus, these toxin-based PD models are not appropriate for therapeutic intervention during pathology progression. Most genetic animal models, such as α-synuclein transgenic mice, lack the selectivity for nigrostriatal pathway and fail to produce dopaminergic neuron degeneration comparable to that in clinical PD [3, 18, 21]. Our Tet-Off conditional transgenic mice enabled the dopaminergic expression of the PD-associated disease protein PARIS with temporal resolution. PARIS is a key pathological nodal point mediating dopaminergic cell death in PD [10]. It accumulates downstream of the E3 ligase parkin or mitochondrial PINK1 dysfunction [11]. PARIS mediates dopaminergic cell death in sporadic PD in response to PFF-induced oxidative stress and c-Abl activation [12, 21]. Owing to the robustness and toxicity of PARIS accumulation, tetracycline-regulated PARIS expression in dopaminergic neurons is sufficient to induce progressive dopaminergic neurodegeneration, resulting in L-DOPA-responsive bradykinesia and motor coordination impairment.

The pan-neuronal CamKIIα-tTA driver was crossed with the TetP-PARIS responder mice to generate PARIS transgenic mice with approximately 30% dopaminergic neuron loss and resulted in premature death due to gastrointestinal dysfunction [8], preventing further characterization of in vivo PARIS pathology. Using the DAT-PF-tTA driver further augmented PARIS expression from the tetracycline-regulatable promoter and prevented unwanted adverse effects of PARIS expression in diverse tissues. We also identified the toxicity of PARIS during dopaminergic neurodevelopment, as PARIS induction during the embryonic stages resulted in a complete lack of midbrain dopaminergic neurons from birth. Temporal regulation of transgene expression in our Tet-Off mouse model allowed us to investigate the pathological functions of PARIS in adult mouse brains. Although we examined PARIS dopaminergic toxicity at 1–4.5 months of age, PARIS pathologies can be studied in aged mouse brains by manipulating the PARIS induction age with simple switching from doxycycline to a regular diet. Thus, our conditional PARIS Tg mice provided an unprecedented PD mouse model with temporally and spatially controlled expression of PD relevant disease proteins. However, the current PARIS Tg model with the adolescent or young adult induction protocol may not be ideal for investigating sporadic PD. The rapid loss of dopaminergic neurons and the relative absence of Lewy pathology suggest that this model might instead be more suited for studying autosomal recessive juvenile PD associated with parkin deletion and substrate PARIS accumulation.

Our PARIS Tg mouse model has some limitations as a PD mouse model. First, the DAT-PF-tTA driver mice have a targeted knock-in of the PF-tTA cassette downstream of the endogenous DAT promoter [22], resulting in the deletion of one copy of the endogenous DAT gene. Although we used DAT-PF-tTA drivers as littermate controls for PARIS Tg mice, and these control mice showed no differences in TH counts or motor performance compared to wild-type mice, as reported in previous studies [22], it would be ideal to use lines with both copies of the DAT gene intact. Additionally, it has been reported that DAT gene expression is also present in astrocytes and is robustly enhanced in the 6-OHDA-lesioned rat striatum, particularly with prolonged L-DOPA treatment lasting seven days [33]. In our analysis, we achieved dopaminergic neuron-selective exogenous PARIS-FLAG expression, without any detectable FLAG signals in GABAergic neurons or astrocytes in the ventral midbrain (Supplementary Fig. 2B, C, D). Furthermore, our snRNA-seq analysis showed no elevation of endogenous DAT expression in astrocytes of PARIS Tg mice compared to littermate controls. It is possible that the DAT-tTA expression in astrocytes is too weak to induce exogenous PARIS transgene expression, especially when compared to the strong PARIS expression driven by DAT-tTA in dopaminergic neurons. This is consistent with UMAP projections showing high levels of DAT expression in dopaminergic neuron clusters without any detectable expression in astrocyte cluster (Supplementary Fig. 8C). However, caution should be taken in the future application of the PARIS Tg mouse model for PD research, especially under prolonged L-DOPA treatment, as the previous 6-OHDA model indicated astrocytic DAT induction by L-DOPA [33].

### Practical application for preclinical evaluation of representative drugs

c-Abl activation has been implicated in PD pathogenesis including Lewy body formation, clearance, and dopaminergic neurodegeneration [17, 18, 20]. Pharmacological c-Abl kinase activity inhibition prevents dopaminergic neurodegeneration in MPTP intoxication, genetic, virally induced PARIS transgenic mice, and PFF injection PD mouse models [12, 20]. We have previously reported that PARIS phosphorylation by c-Abl regulates PARIS transcriptional repressive activity and PARIS-induced PD pathologies in vivo [12]. Consistent with this report, selective dopaminergic PARIS expression also activated c-Abl, and the pharmacological c-Abl inhibition largely prevented PARIS-induced dopaminergic neurotoxicity. The partial rescue of dopaminergic neuronal viability and motor impairment by nilotinib in PARIS Tg mice suggests that signaling pathways other than c-Abl are involved in PARIS-induced PD pathogenesis in vivo. It is also possible that nilotinib may not be optimal for blocking the aberrant c-Abl activity in vivo. c-Abl has been identified as a therapeutic target for blocking α-synuclein aggregation pathology [15–20, 34]. While PARIS-induced c-Abl activation is evident in the ventral midbrain of PARIS Tg mice, we did not observe detectable pS129-α-synuclein-positive Lewy-like inclusions. This absence could be attributed to the rapid progression of dopamine neuron death triggered by PARIS, a substrate of parkin [10]. This finding aligns with the lack of Lewy bodies observed in juvenile Parkinson’s disease cases with parkin deletion [5, 35]. However, Lewy bodies are a pathological hallmark in sporadic Parkinson’s disease (PD) cases, and parkin phosphorylation, along with the accumulation of its substrates (e.g., PARIS, AIMP2), has been reported in postmortem brains from sporadic PD patients [10, 13, 14]. Notably, AIMP2, another parkin substrate, has been shown to induce α-synuclein aggregation in an age-dependent manner in AIMP2 Tg mice [36]. Furthermore, coexpression of AIMP2 and α-synuclein has been reported to exacerbate α-synuclein aggregation pathology [36]. In light of these observations, the development of α-synuclein pathology in PARIS Tg mice may require aging and extended incubation periods. Future studies could explore disease induction protocols that initiate PARIS overexpression at older ages or involve coexpression with α-synuclein to better model α-synuclein aggregation pathology. These modifications may provide further insights into the interplay between PARIS, α-synuclein, and c-Abl activation in the pathogenesis of PD.

Preclinical studies using our mouse model have confirmed the effectiveness of the c-Abl inhibitor, nilotinib, and the antisymptomatic drug, L-DOPA. Although disease-modifying strategies to prevent ongoing degenerative processes are of utmost interest for new drug development, recent studies also focus on improving the efficacy and safety of antisymptomatic dopaminergic drugs. In this regard, PARIS Tg mice with progressive dopaminergic cell death within 2–3 months can replace toxin-induced PD mouse models. Disease progression over several months also provides opportunities for the post-administration of disease-modifying therapies to intervene with the target pathological signaling pathways. Moreover, the near-complete and selective elimination of DA neurons without affecting other brain circuits allows for the potential application of stem cell-based therapies and deep brain stimulation. Compared with the surgical models of 6-OHDA brain stereotaxic injection, our conditional transgenic PD mice can be used as a consistent platform to evaluate diverse PD therapeutic approaches.

The preclinical application of the PARIS Tg model extends beyond testing drug candidates for motor improvement. PARIS Tg mice exhibit significant degeneration of VTA dopaminergic neurons that project to the ventral midbrain, which regulates reward and motivation circuits [37]. This pathology is associated with a mild trend toward depression in PARIS Tg mice, as indicated by the tail suspension immobility assay (Supplementary Fig. 4E). This finding aligns with previous reports that 6-OHDA PD models, which also involve VTA dopaminergic neuron loss, exhibit a depressive phenotype [38, 39]. While further validation with larger sample sizes and both sexes is needed, the PARIS Tg model may be valuable for developing treatments for PD-related depression [40]. Another notable non-motor symptom in PARIS Tg mice is anxiety. Interestingly, this anxiety does not appear to be driven by dopamine transmission, as L-DOPA treatment does not alleviate the symptom (Supplementary Fig. 3L). This suggests that the anxiety phenotype could be secondary to chronic motor dysfunction, possibly involving various neural circuits related to memory, emotion, and stress response [41]. Notably, nilotinib failed to prevent anxiety in PARIS Tg mice, despite its effectiveness in inhibiting dopaminergic neurodegeneration. This may be due to known side effects of nilotinib, which have been previously linked to anxiety [42, 43].

For precision and gender specific application of preclinical evaluation, our PARIS Tg mice hold some limitations. Epidemiological meta-analysis indicates more prevalence of PD in men than women [44]. However, PARIS Tg mice exhibited similar extent of PD features development including TH neuron loss and motor impairments in both genders (Figs. 2B, and 3E, F). This discrepancy could be due to too fast progression of dopamine neurodegeneration induced by PARIS expression in this transgenic PD model as compared with slow and chronic progression of clinical PD. Moreover, induction of PARIS at adolescent or young adult stage might not be sufficient to achieve potential interaction between genders with aging process because male-to-female PD ratio increases with aging in human [45]. Future investigation with PARIS Tg model with transgene induction at old age for each gender would be necessary to potentially evaluate gender and aging effect in PARIS driven PD pathogenesis.

### Key molecular pathogenic pathways induced by dopaminergic PARIS expression in vivo

PD mouse models that can reproduce clinical features of PD are important for dissecting key pathological molecular pathways eventually set in motion in PD pathogenesis. The relevance of PARIS in PD has been shown in several studies [10, 12]. PARIS is a common substrate of the recessive PD genes, PINK1 and parkin. PINK1 phosphorylation of PARIS primes PARIS for parkin-mediated polyubiquitination and proteasomal degradation. Thus, PARIS accumulation simulates recessive PD conditions. Moreover, PARIS accumulation and concomitant c-Abl activation have recently been observed in PFF-induced sporadic PD mouse models [21]. PARIS knockout can abolish PFF-induced PD pathogenesis in vivo [21], indicating an important pathological role of PARIS in sporadic PD. Therefore, the PARIS transgenic model can serve as an in vivo platform for investigating molecular targets associated with diverse PD conditions. Since we evaluated the c-Abl inhibitor, other associated molecular targets could be modulated pharmacologically or genetically to confirm its effectiveness in treating PD.

In this study, we identified that PGC-1α repression by PARIS is dependent on c-Abl-mediated phosphorylation of PARIS at Y137. The initial accumulation of PARIS in dopaminergic neurons likely contributed to PGC-1α repression, resulting in mitochondrial dysfunction and increased oxidative stress. Transcriptomic signatures indicative of mitochondrial deficits and oxidative stress were observed in ventral midbrain dopaminergic neurons through snRNA-seq analysis (Fig. 8D, and Supplementary Fig. 10). c-Abl activation was detected as early as 2 months of age, suggesting that c-Abl-mediated PARIS phosphorylation may amplify PGC-1α repression and exacerbate dopaminergic neurotoxicity. Based on these findings, we propose a pathological feed-forward signaling mechanism involving c-Abl, PARIS, and PGC-1α, which underlies the molecular pathology observed in our PARIS Tg studies (Supplementary Fig. 13). Since c-Abl is known to be activated under PD pathological conditions, the c-Abl-PARIS pathway may contribute to the heightened vulnerability of dopaminergic neurons to PARIS-induced toxicity compared to cortical neurons in CamKIIα-tTA;TetP-PARIS mice [8]. However, this c-Abl-PARIS pathway in relation to neuron-type selective PARIS toxicity needs to be further validated with extended PARIS expression in whole brains of mice.

### Validation of mitochondrial deficits from transcriptomic analysis in PARIS Tg mouse midbrains and further insights from unbiased functional ontology analysis

PARIS was initially identified as a transcriptional repressor of PGC-1α and NRF1 [10]. PGC-1α is a master regulator of mitochondrial biogenesis [46] and regulates antioxidant defense by PGC-1α–NRF1 axis [47]. Consistent with this notion, dopaminergic PARIS accumulation in vivo led to PGC-1α downregulation, which correlated with reduced mitochondrial mass and dysregulation of mitochondrial marker protein expression. It remains unclear why only specific mitochondrial markers were reduced under PARIS-mediated repression of PGC-1α. PGC-1α is a transcriptional coactivator that regulates mitochondrial biogenesis through interactions with key transcription factors such as NRF1, peroxisome proliferator-activated receptor, and estrogen receptor-related α [46]. One possibility is the existence of compensatory pathways that help maintain the expression of certain mitochondrial proteins despite PGC-1α repression. Post-transcriptional regulation might also contribute to differential expression levels of mitochondrial proteins. Nevertheless, consistent with a previous report [24], PARIS-induced PGC-1α repression was associated with a sustained downregulation of COXIV, observed at both 2 and 3 months of age in PARIS Tg mice. Activation of c-Abl, which is responsive to increased oxidative stress, was also observed. Unbiased transcriptome analysis of PARIS Tg mouse midbrains also confirmed that the DEGs were enriched in several functional pathways associated with oxidative stress, mitochondrial dysfunction, and DNA damage (Fig. 6D).

In this study, we identified a large set of potentially PARIS-regulated genes in the ventral midbrain of a PD mouse model. Among the 1.3-fold DEGs, we further analyzed the 1,259 DEGs with twofold differences (Supplementary Fig. 6A). A total of 1,235 and 24 genes were significantly 2 × fold upregulated and downregulated, respectively, in the PARIS Tg mouse midbrain (Supplementary Fig. 6B). These DEGs with significant fold-changes in PARIS Tg mice might have contributed to PD pathogenesis. Further validation of high-priority candidate genes associated with human PD may provide potential biomarkers and therapeutic molecular targets for PD.

We performed a crude comparison of the DEG profiles of PARIS Tg mice from our study with the DEG profiles of postmortem patient brains with PD from previous studies [48]. Although the brain regions used for RNA-seq analysis (ventral midbrain vs. Brodmann Area 9, respectively) were different, 1,440 DEGs (~ 23%) out of 6,182 protein-coding DEGs with significant alterations in PARIS Tg VM were commonly identified in DEG profiles with significant alterations in postmortem patient brains with PD. This comparison suggests a potential role for PARIS in regulating pathological transcriptional alterations in human PD pathogenesis. However, there are only a limited number of patients with PD in bulk RNA-seq analysis for a thorough meta-analysis. Moreover, considering diverse populations, including age, sex, and race, should improve our understanding of this new PD mouse model for a reliable interpretation of DEGs with clinical relevance.

Considering that PARIS is a transcriptional repressor, it is striking that a large portion of DEGs were indeed upregulated in PARIS Tg mouse VM compared to that in age-matched controls. However, this analysis was performed only for DEGs with > 1.3- or 2.0-fold expression change. In fact, when all DEGs with significant alteration (total 8,548 genes) were analyzed, more than 75% of DEGs were downregulated in PARIS Tg mouse VM. This suggests that PARIS accumulation in dopaminergic neurons of the VM may repress a large portion of the overall gene expression, however the DEGs with higher expression changes due to PARIS are those that are upregulated. Many transcription factors (such as *Twist1*, *Sp7*, *Klf5*, *Dmrta1*, *Nupr1*, and *Atf1*) and histone modifiers (such as *Supt6*, *Rbbp5*, *Dpy30*, *Hat1*, *Ash2l*, and *Hdac1*) expression levels were significantly altered in PARIS Tg mouse VM. DEGs may have been regulated by other transcription factors and histone modifications induced by PARIS accumulation in PARIS Tg mice. Whether the DEGs are directly affected by PARIS binding in dopaminergic neurons is unclear because the data was generated from bulk RNA-seq, which measures the average gene expression in ventral midbrain tissues Thus, the DEGs with large fold-changes may originate from neighboring glial cells that transform into inflammatory reactive states.

This study's single-cell resolution transcriptomic analysis provides a valuable resource for utilizing the PARIS Tg model in PD research. While dopaminergic neuron loss is a hallmark of PD, the extensive depletion (5–70%) in PARIS Tg mice limits the direct assessment of PARIS-driven DEGs in these neurons. To address this, snRNA-seq was performed at an earlier stage, where dopaminergic neuron populations were comparable between control and PARIS Tg mice (Fig. 8A). Enriched biological processes in dopaminergic neurons, including cell migration, apoptosis, transmembrane transport, and regulation of cell proliferation, highlight underlying mechanisms of PD. Pathway analysis also implicated MAPK signaling and oxidative phosphorylation (Fig. 8C, F). Notably, the overlap between bulk RNA-seq and snRNA-seq results, including mitochondrial dysfunction and apoptotic mechanisms, underscores the interrelation of findings. Consistent with PGC-1α's role in mitochondrial biogenesis [10, 46], reduced expression of *Nrf1, Sod1, Tfam, Sod2*, and *Atp5b* was observed in dopaminergic neurons with diminished PGC-1α expression in PARIS Tg mice (Fig. 8D, Table S8).

Non-cell-autonomous effects were evident, with a significant increase in reactive astrocytes in PARIS Tg mice, potentially exacerbating dopaminergic neurodegeneration, consistent with previous findings [49]. GO analysis of glial DEGs further implicated apoptotic processes, mitochondrial dysfunction, and lipid metabolism, aligning with bulk RNA-seq and known PD pathologies. Beyond dopamine, changes in the expression of PD-associated genes and alterations in the proportions of glutamatergic, cholinergic, GABAergic, and serotonergic neuronal subtypes were observed in PARIS Tg mice compared to controls, suggesting a broader disruption of neurotransmission that may contribute to PD-associated phenotypic disturbances [50]. Collectively, this study’s bulk and single-nucleus transcriptional profiles provide an unbiased framework for understanding PARIS-driven gene regulation across diverse cell types, advancing insights into PD pathophysiology.

## Materials and methods

### Chemicals and antibodies

Doxycycline (Sigma-Aldrich; catalog no. D9891), nilotinib (Cayman Chemical; catalog no. 641571–10-0), carbidopa (Sigma-Aldrich; catalog no. C1335), and levodopa (Sigma; catalog no. D9628,) were used as received. The primary antibodies utilized were as follows: a rabbit antibody targeting PARIS (Proteintech; catalog no. 24543–1-AP, 1:5000), phosphorylated PARIS [12], phosphorylated c-Abl (Cell Signaling Technology; catalog no. 2868, 1:5000), tyrosine hydroxylase (Novus Biologicals; catalog no. NB300-109, 1:1000), SDHA (Cell Signaling Technology; catalog no. 11998, 1:5000), HSP60 (Cell Signaling Technology; catalog no. 12165, 1:5000), pyruvate dehydrogenase (PDHA, Cell Signaling Technology; catalog no. 3205, 1:5000), VDAC (Cell Signaling Technology; catalog no. 4661, 1:5000), PHB1 (Cell Signaling Technology; catalog no. 2426, 1:5000), GFAP (Abcam; catalog no. ab7260, 1:1000), IBA1 (Wako; catalog no. 019–19741, 1:1000), along with a rabbit antibody to COXIV (Cell Signaling Technology; catalog no. 4850, 1:1000), FLAG (Sigma-Aldrich; catalog no. F7425, 1:1000), MDM4 (Proteintech; catalog no. 17914–1-AP, 1:1000), phosphorylated p53 (Cell Signaling Technology; catalog no. 9284, 1:1000), a mouse antibody specific to c-Abl (Sigma-Aldrich; catalog no. A5844, 1:5000), tyrosine hydroxylase (ImmunoStar; catalog no. 22941, 1:1000), FLAG (Thermo Fisher Scientific; catalog no. F1804, 1:1000), GAD67 (Sigma-Aldrich; catalog no. MAB5406, 1:1000), DAT (Thermo Fisher Scientific; catalog no. MA5-24,796, 1:1000), GFAP (Cell Signaling Technology; catalog no. 3670 1:1000), and a mouse antibody to PGC-1α (Merck Millipore; catalog no. KP9803, 1:1000). The following secondary antibodies utilized were as follows: goat anti mouse-IgG antibody-conjugated Horseradish peroxidase (HRP) (Genetex; catalog no. GTX-213111–01, 1:5000), goat anti-rabbit IgG antibody-conjugated HRP (Genetex; catalog GTX-213110–01, 1:5000), goat anti-rabbit IgG antibody -conjugated biotin (Vector L aboratories; catalog no. BA-1000, 1:1000), mouse anti-β-actin antibody -conjugated HRP (Sigma-Aldrich; catalog no. A3854, 1:10,000), donkey anti-rabbit IgG antibody-conjugated Alexa Fluor 568 (Invitrogen; catalog no. A10042), goat anti-rabbit IgG antibody-conjugated Alexa Fluor 488 (Invitrogen; catalog no. A11008), donkey anti-mouse IgG antibody-conjugated Alexa Fluor 568 (Invitrogen; catalog no. A10037), and donkey anti-mouse IgG antibody-conjugated Alexa Fluor 488 (Invitrogen; catalog no. A21202).

### Animal experiments

#### Tet-Off conditional PARIS transgenic mouse generation

All animals were raised in facilities where they experienced alternating 12-h cycles of light and darkness, with controlled temperature conditions, and had free access to food and water. These animals were managed according to international guidelines and were approved by the Ethics Committee of Sungkyunkwan University (approval number: SKKUIACUC2021-05–19-1), ensuring adherence to ethical standards for experimentation. Every endeavor was undertaken to mitigate animal distress and minimize the count of animals utilized, in accordance with scholarly standards. Tet-off-condition PARIS transgenic mice were obtained by breeding TetP-PARIS responder transgenic mice [8] and DAT-PF-tTA driver knock-in mice [22]. Both genders were included in the subsequent phenotype characterization and preclinical evaluation. Gender information available for some experiments were provided in the Supplementary Table 13. Transgenic mouse genotypes (Wild type, DAT-PF-tTA, and TetP-PARIS) were evaluated based upon PCR amplification using primer sets specifically targeting transgenic or knocked-in constructs (TetP-PARIS: forward primer = GCGCTGCCCTTATTTTCATGTT, reverse primer = CCCGGTTCGACCTTCTAGGG; DAT-PF-tTA: forward primer = AGAAGAAGGAAACAGACTTCCTC, reverse primer = GCTTGTTCTTCACGTGCCAGT; GAPDH: forward primer = TGGGTGGAGTGTCCTTTATCC, reverse primer = TATGCCCGAGGACAATAAGG). Genomic DNA from the tail was obtained using the DirectPCR Lysis Reagent (Viagen Biotech; catalog no. 102 T). For motor rescue by dopamine supplementation, the mice were intraperitoneally injected with a mixture of 5 mg/kg carbidopa and 20 mg/kg levodopa. The nilotinib diet contained 200 mg nilotinib/kg regular diet (Doo Yeol Biotech) and was provided to mice to inhibit brain c-Abl, as described previously [12]. Each littermate mouse was assigned a number code through toe clipping prior to weaning and subsequently subjected to analysis. During the assessment and recording of TH stereology and behavioral tests, experimenters were blinded to the age, genotype, and treatments of the mice.

### Behavioral tests

#### Pole test

Prior to the pole test, mice were trained two times. The 9-mm diameter pole was a 23-inch metal rod wrapped with a bandage gauze. Briefly, the mice were placed on top of the pole with their heads oriented upwards. The duration required to descend to the bottom of the pole was manually recorded with stopwatch and documented. Experimental findings are presented as the average latency period across three trials.

#### Rotarod test

Before the rotarod test, mice underwent a 2-day training period. On the third day, the mice were positioned on a rotating rotarod cylinder where the speed increased, and the time until they fell off, unable to maintain the speed, was measured automatically by the instrument (Rota Rod with Touchscreen, 5 mice, cat no. 76–0770, Panlab). The rotarod speed was slowly increased from 4–40 rpm over 5 min. Test data are presented as the mean latency time from three trials.

#### Open-field test

The open-field test comprised a rectangular wooden box (32 × 32 × 32 cm^3^) divided into 64 (8 × 8) identical regions (4 × 4 cm^2^). The open field was divided into three sectors: border, peripheral, and central. The central sector comprised four (2 × 2) central squares, while the peripheral sector consisted of 12 squares surrounding the central sector. The remaining squares represent border sectors. Each mouse was positioned centrally within the field and given a 15-min period for exploration under low light conditions. The apparatus was cleaned with diluted 70% ethanol after each trial. Locomotor activity was measured by recording the distance traveled using a video tracking system (Smart v3.0 software). Additionally, Indicators of anxiety, including the duration spent in the center and the latency to the initial entry into the center, were assessed.

#### Beam walking test

The beam walking test was performed to assess motor coordination in mice [51]. The endpoint of the beam was designed as a black square box, providing a secure and comfortable space for the mice. On the first day, the mice were trained on the beam walking test using a cylindrical beam with a diameter of 2 cm and a length of 90 cm. Following the training session, the test was conducted on a narrower cylindrical beam with a diameter of 1 cm and a length of 90 cm to evaluate motor coordination. Performance was assessed by measuring the time taken for each mouse to traverse the beam and enter the secure square box. Additionally, the number of slips that occurred while the mice were on the beam was recorded and analyzed.

#### Tail suspension immobility test

The tail suspension test was conducted to evaluate depressive-like behaviors in mice [52]. A 7 cm strip of adhesive tape was attached to the distal end of each mouse's tail. To prevent the mice from climbing up the tape, a 3 cm long tube was placed around the tail before suspension. The mice were suspended approximately 60 cm above the ground, ensuring no physical contact with any surface. Behavioral testing was performed for a total duration of 5 min, during which the mice were recorded using a video camera. The recorded footage was subsequently analyzed to determine the duration of immobility, a key indicator of depressive-like behavior.

### Immunoblotting for mouse brain tissues

Mouse brain subregions, such as the cortex, VM, and CB were identified following previously described procedures [9]. The dissected mouse brain tissues were processed in lysis buffer [0.5% sodium deoxycholic acid and 1% Nonidet P40 (NP40) in phosphate-buffered saline (PBS), pH 7.4 with phosphatase/protease inhibitors] using a Diax 900 homogenizer. The homogenized brain samples were incubated on ice for 30 min to ensure complete lysis, with vortexing every 5 min. Subsequently, they were centrifuged at 14,000 × g for 30 min to isolate the protein. The supernatants were gathered, and the protein levels were assessed employing the BCA Protein Assay Kit (Thermo Fisher Scientific; catalog no. 23225), with bovine serum albumin standards used for quantification. The proteins were subsequently analyzed via immunoblotting with the specified antibodies.

### Western blot analysis

After quantification, the protein samples obtained were brought to a boil with 2 × Laemmli sample buffer (Bio-Rad; catalog no. 161–0737) supplemented with 2-mercaptoethanol (Bio-Rad; catalog no. 1610710) for 5 min. The proteins were processed through SDS-PAGE for separation and then transferred onto nitrocellulose membranes (0.45 μm, Bio-Rad; catalog no. 162–0115) for subsequent immunoblotting with the specified antibodies. The bands were visualized using chemiluminescence (Thermo Fisher Scientific; catalog no.34580). For quantification of band intensities, Analysis was executed utilizing ImageJ software (NIH, http://rsb.info.nih.gov/ij/).

### TH immunohistochemistry and stereological counting

For the analysis of TH- or PARIS-positive neurons, the sections were processed as follows: They were treated with polyclonal rabbit anti-TH (Novus Biologicals; catalog no. NB300-109, 1:1000) or rabbit anti-PARIS (Proteintech; catalog no. 24543–1-AP, 1:1000) antibodies. Subsequently, sequential incubations with goat anti-rabbit IgG antibody-conjugated biotin and streptavidin-conjugated HRP were executed using a Vectastain elite ABC HRP kit (Vector Laboratories; catalog no. PK-6101) according to the manufacturer’s protocol. Afterwards, The TH- or PARIS-positive cells were identified via 3,3-diaminobenzidine (DAB, Sigma-Aldrich catalog no. D4293) as the HRP substrate. The brain sections, which were subjected to immunostaining, underwent counterstaining with Nissl stain. Following visualization of dopamine cells via DAB staining, quantification of dopamine cell count was conducted in the substantia nigra pars compacta and ventral tegmental area utilizing the Optical Fractionator probe of the Stereo Investigator software (MicroBrightfield, Williston, VT). Stereological counting was carried out with the person performing the counting blinded to the treatment of each mouse.

### Dopamine determination in brain by liquid chromatography–tandem mass spectrometry (LC–MS/MS)

Dopamine levels in the brain were determined using a LC–MS/MS system, with slight modifications from previous LC–MS/MS methods [53, 54]. Briefly, the LC–MS/MS system comprised an Agilent 6490 QQQ system equipped with an ESI + Agilent Jet Stream ion source and a 1290 Infinity HPLC system (Agilent Technologies, Santa Clara, CA, USA). For the chromatographic separation, a Synergi polar-RP column (150 × 2.0 mm^2^, 4 μm; Phenomenex, Torrance, CA, USA) was used. The mobile phase was composed of water (pH 3.0) containing a mixture of 10 mM ammonium formate for phase A and acetonitrile for phase B, respectively. The gradient method with 0.2 mL/min was applied as follows: 0–4 min, 95–25% A; 4–5 min, 25% A; 5.0–5.1 min, 25–95% A; and 5.1–13 min, 95% A. The column was set at 30 °C and the injection volume was 2µL. Multiple reaction monitoring mode of dopamine and internal standard (3,4-dihydroxybenzylamine [DHBA]) were as follows: m/z 154.2 → 90.8 for dopamine and m/z 140.1 → 122.9 for IS. the brain samples were homogenized in sevenfold PBS. Subsequently, a 50µL homogenate was deproteinized by adding 100µL methanol containing 10 µg/mL DHBA, and then centrifuged at 14,000 rpm for 15 min at 4 °C. The supernatant was then collected for dopamine LC–MS/MS analysis. The linear range for dopamine was 1–100 ng/mL. The amount of dopamine in the brain was calculated considering the dilution factor (eightfold dilution), and the percentage change compared to the dopamine control level was expressed.

### Immunofluorescence

The animals were intracardially perfused and fixed with ice-cold PBS followed by 4% paraformaldehyde (PFA). The mouse brain was isolated, fixed with 4% PFA for 1 day at 4 °C, and then submerged in 30% sucrose for 2 days at 4 °C for cryoprotection. The cryoprotected brains were sectioned using a sliding microtome (Thermo Fisher Scientific; catalog no. HM430) and assessed immunohistochemically. Coronal sections, each 35 µm thick, were sliced through the brain, encompassing the substantia nigra. The fixed mouse brains were subjected to a blocking reaction at room temperature for 1 h by incubating them in a solution diluted to a final concentration of 5% normal goat serum (Invitrogen; catalog no. 16201) in 0.1% Triton X-100 (Sigma-Aldrich; catalog no. X100) to prevent nonspecific binding of the primary antibody to targets. Following this, the sections were incubated with various combinations of primary antibodies, tailored to each specific experiment, at 4 °C overnight. After incubating with primary antibodies, the brain was washed with PBS, followed by incubation in a PBS solution containing secondary antibodies conjugated with selected fluorescent dyes at room temperature for 1 h. Subsequently, the samples were briefly immersed in a PBS solution containing DAPI for nuclear staining for approximately 1 min. Then, the brain sections mounted on a slide using an immunomounting solution (Thermo Fisher Scientific; catalog no. 9990402). To obtain fluorescent images of the stained brain, we utilized a fluorescence microscope (Zeiss, Oberkochen, Germany; Axio Imager M2). To assess apoptotic neuronal death, the TUNEL assay (Abcam; catalog no. ab661110,) was used to label fragmented DNA according to the manufacturer’s guidelines.

### Mouse midbrain RNA-seq data acquisition

Twelve mouse total RNA samples were prepared from the midbrain following a manual TRIzol-based RNA isolation protocol. Residual genomic DNA was eliminated using RNase-free DNaseI for 30 min at 37 ℃. Library preparation of mRNA-seq was performed with the Illumina TruSeq RNA Sample Preparation Kit v2 (Illumina Inc., San Diego, CA, USA). Briefly, 150 ng RNA was used to isolated poly-A positive mRNA using streptavidin magnetic beads, followed by 200–500 bp fragmented shorter mRNA serves as a template for cDNA synthesis along the random primers. This cDNA then transformed into double-stranded DNA, which end-repaired that allows to ligate specific index adaptors. Purification and amplification for 15 cycles, then the final amplified libraries qualify through the DNA high sensitivity chip on an Agilent 2100 Bioanalyzer (Agilent Technologies, Palo Alto, CA, USA).

Generated library was loaded on a flow cell for the fragment capture through coated complementary oligonucleotides against sequencing adaptors. Then the clusters undergo bridge amplification with hybridization to generate distinct clonal clusters. These clustered libraries were sequenced using Illumina HiSeqTM 2500 according to the manufacturer’s instructions, by which paired-end sequenced for 100 cycles using the TruSeq Rapid SBS kit or Truseq SBS kit v4. The sequencing protocol followed the HiSeq 2500 System User Guide Part # 15,011,190 Rev. V HCS 2.2.58.

### Data filtering

To generate raw sequencing data, HiSeq Control Software (HCS v2.2) was employed for system control and RTA (v1.18) software was used for base calling. Data were generated by the HiSeqTM2500 were processed base calling to convert into nucleotide sequences and saved in the FASTQ-formatted file using the Illumina package bcl2fastq (v1.8.4).

The reads that contain the adaptor sequence, low-quality reads, and high contents of unknown bases were filtered and removed to reduce noise of data before downstream analysis. The filtering was conducted following FastQC v0.11.5 software to assess the quality of RNA-seq library. Then, the reads went through standard quality control according to the referred parameters: (1) primers and/or adaptors aligned reads, (2) reads with low-quality bases (quality value ≤ 5) over 50% per one read, and (3) reads with unknown bases (N bases) over 10%. After filtration process, the remained reads considered “clean reads” and saved as FASTQ-formatted file.

### Bioinformatic data analysis

Clean reads were mapped to the mouse reference genome (UCSC mm10) and the gene sequences were mapped using HISAT2 (Kim et al., 2015) to obtain the BAM file. The BAM files were sorted and indexed with samtools sort, and the number of reads matching each gene in the mouse was counted using HTSeq-count. Using read-count data, DEGs and statistical analyses were performed using DESeq2 (v1.22.2) and R packages (v3.6.1). Briefly, read-count data were filtered (rowMax < 1) and normalized to the DESeq2 protocol. The genes with Lfc2 (log2 ratio of fold-change) between the average of the control and PARIS Tg group was considered as differentially expressed, and the Wald test was performed for statistical analysis (Wald t-test P < 0.05 or Benjamini and Hochberg adjusted P < 0.05). The PCA and heat maps were plotted using the DESeq2 and ggplot2 packages.

For functional grouping analysis, DAVID (https://david.ncifcrf.gov/) was used. Functional enrichment analysis of the gene sets was performed using the GO Functional Classification System (www.geneontology.org). Pathway analysis was performed using the KEGG software (http://kegg.jp).

### LC–MS/MS measurement of bile acids and steroids

The analysis was conducted on an AB Sciex 5500 QTRAP mass spectrometer with an electrospray ionization (ESI) source operating in negative and positive ionization modes. Standard chemicals were purchased from Sigma-Aldrich (cholic acid, catalog no. C1129; pregnenolone, catalog no. 700142P; progesterone, catalog no. P8783; cholesterol, catalog no. C8667; testosterone-d3, catalog no. T5536), and Medchemexpress (cholic acid-d4, cat no. HY-N0324S; cholesterol-d4, cat no. HY-N0322S6). Chromatographic separation was performed on a Synergi™ Polar-RP 80A column (150 × 2 mm, 4 μm, Phenomenex, Torrance, CA, USA) equipped with a SecurityGuard™ guard column (4.0 × 3.0 mm). An isocratic condition for the mobile phase was determined using a mixture of 0.1% formic acid in water (A) and acetonitrile (B) at the flow rate of 0.2 mL/min. The mobile phase composition was optimized for all analytes at a 30:70 ratio of A/B. The total run time for UDCA, CDCA, and cholic acid was fixed to 5 min. On the other hand, progesterone, pregnenolone, and cholesterol require longer run times (20 min). The injection volume was 2.5 µL, and the column and autosampler were kept at 25 °C and 4 °C, respectively. The mass spectrometer was operating in selected source parameters with an ion spray voltage of 5500 V and ion source temperature of 500 °C. UDCA, CDCA, and cholic acid were quantified in the negative ionization mode using cholic acid-D4 as an internal standard (IS). Progesterone, pregnenolone, and cholesterol were quantified in the positive ionization mode with testosterone-D3 as the IS. Data were acquired using multiple reaction monitoring (MRM) with optimized transitions for each analyte. The MRM transitions, collision energy (CE), declustering potential (DP), and Collision Cell Exit Potential (CXP) for each compound are detailed in Table 1. The analyte MRM transitions were similar to those of previous reports [55–57].

**Table 1:** MRM transitions and optimized mass spectrometry parameters for the quantification of bile acids and steroids

Analyte	Ionization Mode	Precursor Ion (m/z)	Product Ion (m/z)	Collision Energy (CE, V)	Declustering Potential (DP, V)	Collision Cell Exit Potential (CXP, V)
Ursodeoxycholic Acid (UDCA)	Negative	437.211	391.3	-26	-40	-15
Chenodeoxycholic Acid (CDCA)	Negative	437.236	391.3	-20	-60	-15
Cholic Acid (CA)	Negative	453.196	407.3	-28	-55	-15
Cholic Acid-D4 (CA-D4)^a^	Negative	457.22	411.3	-22	-45	-15
Progesterone	Positive	315.149	97	27	146	10
Pregnenolone	Positive	299.4	281.4	22	81	5
Cholesterol	Positive	369.211	147.1	33	61	28
Testosterone-D3^b^	Positive	292.117	97.1	29	111	8

^a, b^Internal standard for negative and positive mode, respectively

For determining of analytes in mouse brain samples (striatum from 3 month-old PARIS Tg and littermate control), stock solutions of the analytes were prepared at a concentration of 1 mg/mL in pure ethanol. Further, serial dilution of each stock was made with methanol (MeOH) to obtain the series of standard working stock solutions (1–10,000 ng/mL). An aliquot of the IS stock solution was diluted with MeOH to get the working IS solution with a concentration of 200 ng/mL of each cholic acid-D4 and testosterone-D3. The standards for biological samples were prepared by adding 20 μL of working stock solutions to 20 μL of a 5% BSA solution in PBS as previously reported [58, 59]. Brain samples were homogenized in a fourfold volume of PBS. Brain samples were prepared by adding 20 μL of methanol to 20 μL of brain sample homogenate. 100 μL of MeOH solution containing IS was added to each sample, mixed on vortex for 30 s, and centrifuged at 4 °C at 14,000 rpm for 15 min. An 80 µL volume of the supernatant was collected for the LC–MS/MS analysis. The standard linearity of all analytes was good (correlation coefficient r^2^ > 0.9900) with a concentration range of 1 to 10,000 ng/mL. Brain CDCA, and cholic acid levels were below detection limits.

### Nuclei isolation

Ventral midbrains were dissected from 2-month-old control and PARIS Tg mice. Tissue samples from four control mice and five PARIS Tg mice were pooled for analysis in each group, followed by homogenization using a pestle in ice-cold Nuclei EZ Lysis Buffer (Sigma, #NUC101) containing 0.2U/µl RNase inhibitor for nuclei isolation. The nuclei suspensions were passed through 30 μm strainer to separate nuclei from debris and undigested tissue pieces. Then the samples were washed twice using 1% BSA/PBS without Ca^2+^ and Mg^2+^, containing 0.2U/µl RNase inhibitor at 500 g centrifugation for 5 min at 4 °C. Subsequently, remaining debris were eliminated from the samples using Percoll (Sigma, #P4937) gradient. Gently resuspended samples in cold 1% BSA/PBS containing 0.2U/µl RNase inhibitor without Ca^2+^ and Mg^2+^, then passed through 20 µm EASYstrainer™ SMALL Cell Strainer (GREINER BIO-ONE, #542,120). Isolated cells were counted using LUNA-FX7™ Automated Fluorescence Cell Counter (Logos Biosystems).

### Single nucleus RNA sequencing library construction

Single nucleus RNA-seq libraries were generated according to the instruction of 10 × Chromium Single Cell 3’ v4 protocol (10 × Genomics, No. CG000731), using 10 × Chromium iX and GEM-X Single cell 3’ Reagent v4 kits (10 × genomics, PN-1000692). Briefly, the nuclei suspension (target recovery 10,000 nuclei) was reversely transcribed with master mix and loaded with Single Cell 3′ Gel Beads and Partitioning Oil into a Single Cell GEM-X 3’ Chip (10 × genomics, PN-1000690) to generate single-nucleus Gel Bead-in-emulsion (GEM). Poly(A)-tailed mRNAs from single nucleus were attached with unique barcode and then undergone reverse transcription within GEM. These processed full-length cDNAs from mRNAs through GEM-RT incubation were amplified with PCR. For the construction of the 3’ Gene Expression Library, the enriched cDNA was followed by putting through the enzymatic fragmentation, end repair, A-tailing, adaptor ligation and sample index PCR.

### snRNA-seq analysis

Raw sequencing data (FASTQ) was converted to the data compatible with Seurat software using Cell Ranger software with standard analysis workflow, recommended by manufacturer (10 × Genomics). Data analysis was mainly performed in R 4.4.0 using the Seurat software (v.4.3.0). In detail, firstly, the data matrix of 2 samples created from Cell Ranger software were loaded separately, and then created into Seurat object. Each of 2 Seurat objects were combined, and normalized by “NormalizeData” function. To extract the variable features using “FindVariableFeatures” function, selection method was set as “vst”, and number of features was set as 40,000. Using integration anchors, which was found by “FindIntegrationAnchors” function, 2 combined Seurat objects were integrated. Integrated data was filtered for quality, to select the cells with number of expressed genes (nFeature) are under 15,000, and with total number of RNA reads are under 40,000. The cut-off number was chosen by naked eye after plotting violin plot using “VlnPlot” function for each of Seurat object data. Filtered data was then batch-corrected, scaled, PC (Principal Component) analyzed, clustered with the resolution of 0.5. Finally, the data was performed dimensional reduction using UMAP with dims = 1: 10, and visualized with “DimPlot”.

For cell-type identification of clustered data, “HGNChelper” software was used [60]. To perform identification, database of tissue-specific biomarker genelists recommended from the same software was used with slight modification by user. To analyze and extract differentially expressed genes (DEGs) between clusters, samples, and cell types, “FindMarkers” function was used, with several options of min.pct (minimal percentage of cells which gene expressed), pseudocont.use (pseudo-value applied during calculating fold-change when expression value is 0), and logfc.threshold (fold-change difference between 2 subjects). “FindAllMarkers” function was also used to get the cluster-specific expressed genes compared to the other clusters. After performing FindMarkers, the genes, which are average log2FC > 0.5 and non-parametric Wilcoxon rank sum test p-value < 0.05, were considered as DEGs. To extract cells identified as specific cell type, “subset” function was used. Each subset was clustered again, dimension-reduced and visualized by UMAP again. Plots including UMAP, stacked violin plot, split violin plot, dot plot, volcano plot and correlational coefficient was made using R 4.4.0 and ggplot2 software (v 3.5.1). Other plots, such as stacked barplots and histogram were plotted using 3rd-party software. For the functional grouping analysis, DAVID (https://david.ncifcrf.gov/) website was used.

### Statistical analyses

Quantitative data are presented as mean ± standard error of the mean. Statistical significance was assessed either by an unpaired two-tailed Student’s *t*-test (two-group comparisons) or one-way analysis of variance with Tukey’s HSD post hoc analysis (comparisons of more than three groups with one independent variable) or two-way analysis of variance with Tukey’s HSD post hoc analysis (comparisons of more than three groups with two independent variables). Differences with *P* < 0.05 were considered statistically significant. GraphPad Prism software (ver.8.0) was used to prepare all plots and statistical analyses.

## Supplementary Information

Additional file 1. Supplementary movie 1. Motor deficit assessment of 3 month old PARIS Tg and control mouse using pole test. Supplementary movie 2. Motor deficit assessment of 2 month old PARIS Tg and control mouse using beam walking test. Supplementary movie 3. L-DOPA rescues motor impairments in PARIS Tg mice. Supplementary movie 4. Nilotinib rescues motor impairments in PARIS Tg mice.

Additional file 2. Supplementary table 1. Differentially expressed genes (DEGs) associated with functional annotation and mitochondrial dysfunction. List of mitochondrial dysfunction-associated genes for bubble plot DAVID functional annotation in PARIS Tg, related to Fig. 6D. Supplementary table 2. Differentially expressed genes (DEGs; more than 2× fold alteration) in the midbrains of PARIS Tg mice. List of 2× fold DEGs PARIS-driven transcriptomic alterations in the midbrains of Tg mice, related to Supplementary fig 5B. Supplementary table 3. Cell type proportion. Proportions of each cell type cluster, including major cell types and their subpopulation of neurons, astrocytes, and microglia. Supplementary table 4. Neuron DEG log2FC. List of DEGs in neurons. (Filtration using log2FC >|0.2|) Supplementary table 5. Neuron subpopulation gene expression level. Supplementary table 6. Neuron subpopulation DEG log2FC. List of DEGs in subpopulation of neurons. (Filtration using log2FC; glutamatergic >|0.3|, cholinergic >|0.2|, dopaminergic >|0.12|, GABAergic >|0.2|, and serotonergic >|0.11|). Supplementary table 7. Neuron subpopulation GO ontology. Supplementary table 8. Dopaminergic neuron KEGG analysis. Supplementary table 9. Astrocyte subpopulation DEG log2FC. List of DEGs in subpopulation of astrocytes. (Filtration using log2FC; a0>|0.25|, a1>|0.4|, a2>|0.4|, a3>|0.25|, a4>|0.35|, a5>|0.55|, and a6>|0.4|). Supplementary table 10. Astrocyte subpopulation GO ontology. Supplementary table 11. Microglia subpopulation DEG log2FC. List of DEGs in subpopulation of microglia. (Filtration using log2FC; m0>|0.25|, m1>|0.3|, m2>|0.25|, m3>|0.25|, m4>|0.45|, and m5>|0.85|). Supplementary table 12. Microglia subpopulation GO ontology. Supplementary table 13. Information on number of male and female mice used in each experimental group.

Additional file 3.

## Data Availability

All associated figures, tables, and movies are included in the manuscript. The original raw datasets used and/or analysed during the current study are available from the corresponding author on reasonable request.

## Abbreviations

PARIS: Parkin-interacting substrate
PD: Parkinson’s disease
DEGs: Differentially expressed genes
VM: Ventral midbrains
AIMP2: Aminoacyl tRNA synthetase interacting multifunctional protein 2
tTA: Tetracycline regulatable transcription activator
Tg: Transgenic
DAT: Dopamine transporter
TH: Tyrosine hydroxylase
CB: Cerebellum
GABA: Gamma-aminobutyric acid
VTA: Ventral tegmental area
STR: Striatum
NAc: Nucleus accumbens
SDHA: Succinate dehydrogenase complex flavoprotein subunit A
HSP60: Heat shock protein 60
PDHA: Pyruvate dehydrogenase E1 subunit alpha 1
VDAC: Voltage-dependent anion channel 1
PHB1: Prohibitin 1
COXIV: Cytochrome c oxidase subunit IV
GO: Gene ontology
LC-MS: Liquid chromatography-mass spectrometry
UDCA: Ursodeoxycholic acid
MOMP: Mitochondrial outer membrane permeabilization
snRNA-seq: Single nucleus RNA sequencing
UMAP: Uniform Manifold Approximation and Projection
