# Supplementary material for: Preclinical studies and transcriptome analysis in a model of Parkinson’s disease with dopaminergic ZNF746 expression
Source: Mol Neurodegener. 2025 Feb 28;20:24. doi: 10.1186/s13024-025-00814-3 (PMC11871723; doi:10.1186/s13024-025-00814-3)
Supplement: Supplementary file 2 — Additional file 2. Supplementary table 1. Differentially expressed genes (DEGs) associated with functional annotation and mitochondrial dysfunction. List of mitochondrial dysfunction-associated genes for bubble plot DAVID functional annotation in PARIS Tg, related to Fig. 6D. Supplementary table 2. Differentially expressed genes (DEGs; more than 2× fold alteration) in the midbrains of PARIS Tg mice. List of 2× fold DEGs PARIS-driven transcriptomic alterations in the midbrains of Tg mice, related to Supplementary fig 5B. Supplementary table 3. Cell type proportion. Proportions of each cell type cluster, including major cell types and their subpopulation of neurons, astrocytes, and microglia. Supplementary table 4. Neuron DEG log2FC. List of DEGs in neurons. (Filtration using log2FC >|0.2|) Supplementary table 5. Neuron subpopulation gene expression level. Supplementary table 6. Neuron subpopulation DEG log2FC. List of DEGs in subpopulation of neurons. (Filtration using log2FC; glutamatergic >|0.3|, cholinergic >|0.2|, dopaminergic >|0.12|, GABAergic >|0.2|, and serotonergic >|0.11|). Supplementary table 7. Neuron subpopulation GO ontology. Supplementary table 8. Dopaminergic neuron KEGG analysis. Supplementary table 9. Astrocyte subpopulation DEG log2FC. List of DEGs in subpopulation of astrocytes. (Filtration using log2FC; a0>|0.25|, a1>|0.4|, a2>|0.4|, a3>|0.25|, a4>|0.35|, a5>|0.55|, and a6>|0.4|). Supplementary table 10. Astrocyte subpopulation GO ontology. Supplementary table 11. Microglia subpopulation DEG log2FC. List of DEGs in subpopulation of microglia. (Filtration using log2FC; m0>|0.25|, m1>|0.3|, m2>|0.25|, m3>|0.25|, m4>|0.45|, and m5>|0.85|). Supplementary table 12. Microglia subpopulation GO ontology. Supplementary table 13. Information on number of male and female mice used in each experimental group. [file 13024_2025_814_MOESM2_ESM.zip › Supplemental table 1.docx]

**Table 1. Mitochondrial dysfunction associated genes list for bubble plot DAVID functional annotation in PARIS Tg**

| **Category** | **Term** | **Count** | **%** | **P-value** | **Genes** | **List Total** | **Pop Hits** | **Pop Total** | **Fold Enrichment** | **Bonferrori** | **Benjamini** | **FDR** |
| --- | --- | --- | --- | --- | --- | --- | --- | --- | --- | --- | --- | --- |
| GOTERM_MF_DIRECT | GO:0016491~oxidoreductase activity | 32 | 28.57 | 1.52E-20 | CTBP2, CTBP1, ALKBH7, ALKBH6, ALKBH8, ALKBH5, ALKBH4, NNT, FADS6, PNPO, AOX1, QSOX1, QSOX2, XDH, SNCA, AOC3, AOC1, AOC2, SMOX, SOD2, SUOX, SOD1, TH, ACOX1, CAT, PCYOX1L, GAPDHS, NOX4, ACOX3, NOX1, PCYOX1, PAOX | 108 | 604 | 17446 | 8.558253618 | 5.31E-18 | 5.31E-18 | 4.74E-18 |
| GOTERM_BP_DIRECT | GO:0055114~oxidation-reduction process | 32 | 28.57 | 4.69E-19 | CTBP2, CTBP1, ALKBH7, ALKBH6, ALKBH8, ALKBH5, ALKBH4, NNT, FADS6, PNPO, AOX1, QSOX1, QSOX2, XDH, SNCA, AOC3, AOC1, AOC2, SMOX, SOD2, SUOX, SOD1, TH, ACOX1, CAT, PCYOX1L, GAPDHS, NOX4, ACOX3, NOX1, PCYOX1, PAOX | 112 | 676 | 18082 | 7.642434489 | 6.70E-16 | 6.70E-16 | 6.28E-16 |
| GOTERM_BP_DIRECT | GO:0043066~negative regulation of apoptotic process | 25 | 22.32 | 5.14E-14 | PRKAA1, PTEN, XIAP, PARK7, PHB2, HIF1A, AQP1, PIDD1, AKT2, CASP3, AKT1, CASP2, BCL2A1B, SNCA, MGMT, TRAF2, SOD2, NFKB1, SOD1, DDB1, PINK1, CAT, BCL2, MDM2, BIRC5 | 112 | 566 | 18082 | 7.131025997 | 7.34E-11 | 2.45E-11 | 2.29E-11 |
| GOTERM_CC_DIRECT | GO:0005829~cytosol | 39 | 34.82 | 1.26E-13 | LRRK2, HTRA2, XIAP, FOXO4, PARK7, HIF1A, ALKBH8, SOX2, CASP9, CASP7, CASP8, SUMO1, CASP6, AKT2, CASP3, MLST8, AKT1, AOX1, BID, XDH, SNCA, RIPK3, RIPK2, SIAH1A, TRAF2, SUOX, MTOR, NFKB1, SOD1, NFKB2, PINK1, TH, CAT, AKT1S1, BCL2, MDM2, BIRC5, GAPDH, NFE2L2 | 111 | 1784 | 19662 | 3.872348806 | 2.71E-11 | 2.71E-11 | 2.46E-11 |
| GOTERM_CC_DIRECT | GO:0005737~cytoplasm | 74 | 66.07 | 2.54E-12 | LRRK2, PTEN, BRCA1, PARK7, AQP2, PHB2, AQP3, ALKBH6, AQP1, ALKBH8, SOX2, CASP9, PIDD1, CASP7, CASP8, ALKBH4, SUMO1, CASP6, AKT2, CASP3, CASP4, AKT3, MLST8, AKT1, CASP2, AOX1, PARP1, RIPK3, RIPK2, RIPK4, SIAH1A, TRAF2, TRAF1, CDC25C, CDC25A, DDB1, ACOX1, AKT1S1, GAPDHS, BIRC5, GAPDH, PAOX, PRKAA1, DIABLO, CTBP1, XIAP, FOXO6, FOXO4, HIF1A, PNPO, RIPK1, BID, XDH, SNCA, AOC3, CASP8AP2, NFE2, SMOX, SOD2, MTOR, NFKB1, SOD1, NFKB2, PINK1, TH, TDP2, BCL2, MDM2, NFE2L3, ATM, NFKBIE, NFE2L1, NOX1, NFE2L2 | 111 | 6631 | 19662 | 1.97677575 | 5.45E-10 | 2.73E-10 | 2.47E-10 |
| GOTERM_BP_DIRECT | GO:0007568~aging | 15 | 13.39 | 2.98E-12 | PTEN, HTRA2, FOXO4, SOD2, SOD1, NFKB2, CASP9, CASP7, UCP3, CAT, AKT1, CASP2, NOX4, NFE2L2, SNCA | 112 | 173 | 18082 | 13.99824525 | 4.25E-09 | 1.06E-09 | 9.97E-10 |
| GOTERM_MF_DIRECT | GO:0042802~identical protein binding | 24 | 21.43 | 3.18E-12 | CASP8AP2, RIPK3, PARP1, LRRK2, PTEN, SIAH1A, XIAP, TRAF2, PARK7, FOXO4, TRAF1, SOD2, NFKB1, SOD1, RAD52, CASP8, CASP6, BCL2, MDM2, AKT1, BIRC5, RIPK1, GAPDH, SNCA | 108 | 625 | 17446 | 6.203022222 | 1.11E-09 | 5.55E-10 | 4.95E-10 |
| GOTERM_CC_DIRECT | GO:0005634~nucleus | 69 | 61.61 | 1.28E-11 | PTEN, BRCA1, PARK7, PHB2, AQP3, ALKBH6, AQP1, ALKBH8, SOX2, CASP9, PIDD1, SOX3, ALKBH5, CASP7, SOX1, CASP8, ALKBH4, SUMO1, CASP6, AKT2, CASP3, SUMO2, AKT3, AKT1, CASP2, PARP1, SIAH1A, CDC25C, CDC25A, DDB2, DDB1, ACOX1, AKT1S1, GAPDHS, BIRC5, VDAC1, GAPDH, PRKAA1, CTBP2, CTBP1, XIAP, HTRA2, FOXO6, FOXO4, HIF1A, SNCA, CASP8AP2, NFE2, PHC1, SMOX, MGMT, MTOR, NFKB1, SOD1, NFKB2, RAD52, RAD50, PINK1, TH, TDP2, BCL2, MDM2, NFE2L3, NOX4, ATM, NFKBID, NFE2L1, ATR, NFE2L2 | 111 | 6019 | 19662 | 2.030623746 | 2.75E-09 | 9.15E-10 | 8.30E-10 |
| GOTERM_BP_DIRECT | GO:0043065~positive regulation of apoptotic process | 18 | 16.07 | 2.24E-11 | DIABLO, RIPK2, PTEN, SIAH1A, HTRA2, HIF1A, CASP9, CASP8, CASP6, CASP3, AKT1, CASP2, NOX4, ATM, RIPK1, BCL2A1D, BID, BCL2A1B | 112 | 335 | 18082 | 8.674733475 | 3.20E-08 | 6.41E-09 | 6.01E-09 |
| GOTERM_CC_DIRECT | GO:0005739~mitochondrion | 35 | 31.25 | 3.05E-11 | DIABLO, LRRK2, PTEN, UCP1, HTRA2, PARK7, ALKBH7, PHB2, CASP9, CASP8, NNT, UCP3, CASP4, AKT1, CASP2, RIPK1, BID, SNCA, CASP8AP2, RIPK3, PARP1, SOD2, SUOX, MTOR, NFKB1, SOD1, PINK1, TH, ACOX1, CAT, BCL2, NOX4, VDAC1, ACOX3, GAPDH | 111 | 1721 | 19662 | 3.602399611 | 6.56E-09 | 1.64E-09 | 1.49E-09 |
| GOTERM_BP_DIRECT | GO:0034599~cellular response to oxidative stress | 9 | 8.04 | 4.57E-09 | RAD52, PINK1, PARP1, MGMT, LRRK2, PARK7, NFE2L1, NFE2L2, SNCA | 112 | 63 | 18082 | 23.06377551 | 6.53E-06 | 8.16E-07 | 7.66E-07 |
| GOTERM_BP_DIRECT | GO:0006974~cellular response to DNA damage stimulus | 17 | 15.18 | 5.87E-09 | PARP1, MGMT, BRCA1, ALKBH7, ALKBH8, DDB2, RAD52, DDB1, CASP9, PIDD1, RAD50, TDP2, CASP3, BCL2, AKT1, ATM, ATR | 112 | 420 | 18082 | 6.534736395 | 8.38E-06 | 9.31E-07 | 8.73E-07 |
| GOTERM_BP_DIRECT | GO:0006979~response to oxidative stress | 11 | 9.82 | 9.05E-09 | DUOX1, PINK1, LRRK2, CAT, BCL2, AKT1, PARK7, SOD2, NFKB1, DUOX2, SOD1 | 112 | 133 | 18082 | 13.35271214 | 1.29E-05 | 1.29E-06 | 1.21E-06 |
| GOTERM_BP_DIRECT | GO:0051402~neuron apoptotic process | 8 | 7.14 | 1.02E-08 | CASP7, DIABLO, CASP3, SIAH1A, BCL2, XIAP, ATM, GAPDH | 112 | 45 | 18082 | 28.7015873 | 1.45E-05 | 1.32E-06 | 1.24E-06 |
| GOTERM_CC_DIRECT | GO:0043234~protein complex | 19 | 16.96 | 1.21E-08 | PRKAA1, PARP1, RIPK2, XIAP, TRAF2, BRCA1, AQP2, PHB2, NFKB1, SOD1, DDB2, RAD52, CASP8, CASP4, AKT1S1, MDM2, AKT1, RIPK1, VDAC1 | 111 | 628 | 19662 | 5.359184025 | 2.61E-06 | 5.21E-07 | 4.73E-07 |
| GOTERM_MF_DIRECT | GO:0019899~enzyme binding | 16 | 14.29 | 1.26E-08 | PARP1, PTEN, TRAF2, PARK7, FOXO4, BRCA1, SOD2, HIF1A, SOD1, TH, CAT, MDM2, AKT1, BIRC5, GAPDH, SNCA | 108 | 384 | 17446 | 6.730709877 | 4.39E-06 | 7.31E-07 | 6.52E-07 |
| GOTERM_BP_DIRECT | GO:0071456~cellular response to hypoxia | 10 | 8.93 | 1.38E-08 | PRKAA1, PINK1, PTEN, BCL2, MDM2, AKT1, HIF1A, MTOR, AQP1, NFE2L2 | 112 | 103 | 18082 | 15.67441054 | 1.97E-05 | 1.65E-06 | 1.54E-06 |
| GOTERM_BP_DIRECT | GO:0042542~response to hydrogen peroxide | 8 | 7.14 | 6.30E-08 | PRKAA1, CASP6, CASP3, CAT, BCL2, PARK7, SOD2, SOD1 | 112 | 58 | 18082 | 22.26847291 | 9.00E-05 | 6.43E-06 | 6.03E-06 |
| GOTERM_CC_DIRECT | GO:0016605~PML body | 9 | 8.04 | 6.54E-08 | CASP8AP2, NFE2, SUMO1, TDP2, SUMO2, PTEN, PARK7, MTOR, ATR | 111 | 96 | 19662 | 16.60641892 | 1.41E-05 | 2.34E-06 | 2.13E-06 |
| GOTERM_BP_DIRECT | GO:0043154~negative regulation of cysteine-type endopeptidase activity involved in apoptotic process | 8 | 7.14 | 1.74E-07 | AKT2, MDM2, AKT1, XIAP, BIRC5, BCL2A1D, AQP1, SNCA | 112 | 67 | 18082 | 19.2771855 | 2.49E-04 | 1.56E-05 | 1.46E-05 |
| GOTERM_BP_DIRECT | GO:0006919~activation of cysteine-type endopeptidase activity involved in apoptotic process | 8 | 7.14 | 1.74E-07 | CASP9, CASP8AP2, PIDD1, CASP8, DIABLO, BID, XDH, SNCA | 112 | 67 | 18082 | 19.2771855 | 2.49E-04 | 1.56E-05 | 1.46E-05 |
| GOTERM_MF_DIRECT | GO:0051287~NAD binding | 7 | 6.25 | 1.05E-06 | PARP1, CTBP2, NNT, CTBP1, GAPDHS, AOX1, GAPDH | 108 | 55 | 17446 | 20.55925926 | 3.66E-04 | 5.23E-05 | 4.66E-05 |
| GOTERM_MF_DIRECT | GO:0031625~ubiquitin protein ligase binding | 12 | 10.71 | 1.36E-06 | PINK1, CASP8, SUMO1, SUMO2, BCL2, MDM2, RIPK1, TRAF2, TRAF1, BRCA1, BID, HIF1A | 108 | 284 | 17446 | 6.825508607 | 4.75E-04 | 5.94E-05 | 5.29E-05 |
| GOTERM_BP_DIRECT | GO:0072593~reactive oxygen species metabolic process | 6 | 5.36 | 3.40E-06 | DUOX1, NNT, LRRK2, BCL2, NOX4, SOD1 | 112 | 38 | 18082 | 25.49154135 | 0.004844 | 2.70E-04 | 2.53E-04 |
| GOTERM_CC_DIRECT | GO:0005758~mitochondrial intermembrane space | 7 | 6.25 | 3.93E-06 | PINK1, DIABLO, CAT, HTRA2, PARK7, SUOX, SOD1 | 111 | 75 | 19662 | 16.53261261 | 8.45E-04 | 1.06E-04 | 9.59E-05 |
| GOTERM_BP_DIRECT | GO:0006631~fatty acid metabolic process | 9 | 8.04 | 5.34E-06 | PRKAA1, TH, FADS6, ACOX1, UCP3, BRCA1, ACOX3, ALKBH7, SNCA | 112 | 156 | 18082 | 9.314217033 | 0.007594 | 3.81E-04 | 3.58E-04 |
| GOTERM_MF_DIRECT | GO:0005515~protein binding | 47 | 41.96 | 5.97E-06 | PRKAA1, CTBP2, CTBP1, LRRK2, PTEN, UCP1, XIAP, PARK7, AQP5, PHB2, HIF1A, SOX2, CASP9, DUOX1, PIDD1, CASP8, SUMO1, CASP6, AKT2, AKT3, AKT1, CASP2, RIPK1, BID, XDH, CASP8AP2, NFE2, PHC1, RIPK3, PARP1, SIAH1A, TRAF2, TRAF1, CDC25C, MTOR, NFKB1, SOD1, BCL2, MDM2, BIRC5, ULK2, ATM, VDAC1, NFKBID, NFE2L1, ATR, NFE2L2 | 108 | 4092 | 17446 | 1.8553863 | 0.002082 | 1.89E-04 | 1.69E-04 |
| GOTERM_BP_DIRECT | GO:0033138~positive regulation of peptidyl-serine phosphorylation | 7 | 6.25 | 6.16E-06 | PINK1, RIPK2, AKT2, BCL2, AKT1, PARK7, SNCA | 112 | 74 | 18082 | 15.27195946 | 0.008762 | 4.18E-04 | 3.92E-04 |
| GOTERM_BP_DIRECT | GO:0043524~negative regulation of neuron apoptotic process | 9 | 8.04 | 6.44E-06 | PINK1, BCL2, XIAP, BIRC5, PARK7, SOD2, HIF1A, SNCA, SOD1 | 112 | 160 | 18082 | 9.081361607 | 0.009148 | 4.18E-04 | 3.92E-04 |
| GOTERM_BP_DIRECT | GO:0071280~cellular response to copper ion | 4 | 3.57 | 7.74E-06 | AOC1, AQP2, AQP1, SNCA | 112 | 7 | 18082 | 92.25510204 | 0.010992 | 4.81E-04 | 4.51E-04 |
| GOTERM_BP_DIRECT | GO:0007005~mitochondrion organization | 7 | 6.25 | 1.04E-05 | PINK1, PARP1, LRRK2, HTRA2, PARK7, SOD2, PHB2 | 112 | 81 | 18082 | 13.95216049 | 0.01478 | 5.96E-04 | 5.59E-04 |
| GOTERM_MF_DIRECT | GO:0016706~oxidoreductase activity, acting on paired donors, with incorporation or reduction of molecular oxygen, 2-oxoglutarate as one donor, and incorporation of one atom each of oxygen into both donors | 5 | 4.46 | 1.53E-05 | ALKBH5, ALKBH4, ALKBH7, ALKBH6, ALKBH8 | 108 | 25 | 17446 | 32.30740741 | 0.005332 | 4.46E-04 | 3.97E-04 |
| GOTERM_BP_DIRECT | GO:0050665~hydrogen peroxide biosynthetic process | 4 | 3.57 | 1.84E-05 | DUOX1, SOD2, DUOX2, SOD1 | 112 | 9 | 18082 | 71.75396825 | 0.025948 | 9.39E-04 | 8.81E-04 |
| GOTERM_BP_DIRECT | GO:0051881~regulation of mitochondrial membrane potential | 5 | 4.46 | 2.46E-05 | PINK1, BCL2, PARK7, SOD2, SOD1 | 112 | 28 | 18082 | 28.82971939 | 0.03449 | 0.00121 | 0.0011357 |
| GOTERM_BP_DIRECT | GO:0006281~DNA repair | 11 | 9.82 | 2.64E-05 | RAD52, DDB1, RAD50, PARP1, MGMT, TDP2, ATM, BRCA1, MTOR, DDB2, ATR | 112 | 318 | 18082 | 5.584624888 | 0.036964 | 0.001255 | 0.0011781 |
| GOTERM_BP_DIRECT | GO:2000378~negative regulation of reactive oxygen species metabolic process | 5 | 4.46 | 2.84E-05 | PINK1, BCL2, VDAC1, BRCA1, HIF1A | 112 | 29 | 18082 | 27.83559113 | 0.039713 | 0.001307 | 0.0012266 |
| GOTERM_BP_DIRECT | GO:0016242~negative regulation of macroautophagy | 4 | 3.57 | 3.58E-05 | PINK1, LRRK2, QSOX1, MTOR | 112 | 11 | 18082 | 58.70779221 | 0.049893 | 0.001519 | 0.0014258 |
| GOTERM_BP_DIRECT | GO:2000379~positive regulation of reactive oxygen species metabolic process | 5 | 4.46 | 3.72E-05 | RIPK3, NOX4, XDH, NOX1, NFE2L2 | 112 | 31 | 18082 | 26.03974654 | 0.05179 | 0.001519 | 0.0014258 |
| GOTERM_BP_DIRECT | GO:0046677~response to antibiotic | 5 | 4.46 | 3.72E-05 | CASP9, AOC3, AOC1, CASP8, SOD1 | 112 | 31 | 18082 | 26.03974654 | 0.05179 | 0.001519 | 0.0014258 |
| GOTERM_BP_DIRECT | GO:0042493~response to drug | 11 | 9.82 | 4.53E-05 | AOC1, MGMT, CAT, PTEN, BCL2, MDM2, SOD2, AQP1, SNCA, ATR, SOD1 | 112 | 339 | 18082 | 5.238674673 | 0.062675 | 0.00171 | 0.0016044 |
| GOTERM_BP_DIRECT | GO:0071363~cellular response to growth factor stimulus | 6 | 5.36 | 4.55E-05 | TH, CAT, MDM2, HTRA2, AKT1, RIPK1 | 112 | 64 | 18082 | 15.13560268 | 0.062909 | 0.00171 | 0.0016044 |
| GOTERM_CC_DIRECT | GO:0005654~nucleoplasm | 26 | 23.21 | 5.30E-05 | CTBP1, PTEN, FOXO4, BRCA1, ALKBH8, SOX2, CASP8, SUMO1, CASP6, AKT3, PNPO, AKT1, NFE2, PHC1, PARP1, MGMT, NFKB1, DDB2, SOD1, NFKB2, DDB1, RAD50, ACOX1, MDM2, ATM, ATR | 111 | 1935 | 19662 | 2.380110343 | 0.011321 | 0.001139 | 0.0010326 |
| GOTERM_BP_DIRECT | GO:0051092~positive regulation of NF-kappaB transcription factor activity | 7 | 6.25 | 5.66E-05 | RIPK3, RIPK2, RIPK4, CAT, RIPK1, TRAF2, TRAF1 | 112 | 109 | 18082 | 10.36811927 | 0.077674 | 0.002021 | 0.0018968 |
| GOTERM_BP_DIRECT | GO:0045471~response to ethanol | 7 | 6.25 | 8.40E-05 | CASP8, TH, MGMT, CAT, PTEN, BCL2, SOD1 | 112 | 117 | 18082 | 9.659188034 | 0.113103 | 0.002791 | 0.0026192 |
| GOTERM_CC_DIRECT | GO:0009898~cytoplasmic side of plasma membrane | 5 | 4.46 | 1.37E-04 | TH, DIABLO, PTEN, HTRA2, TRAF2 | 111 | 47 | 19662 | 18.84416331 | 0.029116 | 0.002686 | 0.0024362 |
| GOTERM_BP_DIRECT | GO:0001933~negative regulation of protein phosphorylation | 6 | 5.36 | 1.67E-04 | LRRK2, PTEN, PARK7, XDH, MTOR, SNCA | 112 | 84 | 18082 | 11.53188776 | 0.212354 | 0.004973 | 0.0046662 |
| GOTERM_BP_DIRECT | GO:0001666~response to hypoxia | 8 | 7.14 | 1.83E-04 | ALKBH5, TH, UCP3, CAT, NOX4, ATM, SOD2, HIF1A | 112 | 192 | 18082 | 6.726934524 | 0.229642 | 0.005324 | 0.0049959 |
| GOTERM_BP_DIRECT | GO:1902177~positive regulation of oxidative stress-induced intrinsic apoptotic signaling pathway | 3 | 2.68 | 2.22E-04 | PARK7, NOX1, SOD1 | 112 | 4 | 18082 | 121.0848214 | 0.271998 | 0.005989 | 0.0056199 |
| GOTERM_BP_DIRECT | GO:0014823~response to activity | 5 | 4.46 | 2.30E-04 | PRKAA1, TH, UCP3, CAT, SOD2 | 112 | 49 | 18082 | 16.47412536 | 0.280329 | 0.006091 | 0.0057158 |
| GOTERM_MF_DIRECT | GO:0097110~scaffold protein binding | 5 | 4.46 | 3.11E-04 | CASP8, CASP4, MDM2, XIAP, PARK7 | 108 | 53 | 17446 | 15.23934312 | 0.10281 | 0.006026 | 0.00537 |
| GOTERM_MF_DIRECT | GO:0032403~protein complex binding | 10 | 8.93 | 3.57E-04 | AOC1, CASP8, RIPK3, LRRK2, CASP3, ATM, RIPK1, VDAC1, TRAF2, HIF1A | 108 | 358 | 17446 | 4.512207738 | 0.117302 | 0.006119 | 0.0054529 |
| GOTERM_MF_DIRECT | GO:0042803~protein homodimerization activity | 15 | 13.39 | 3.67E-04 | AOC3, AOC1, CTBP2, RIPK2, CTBP1, LRRK2, SIAH1A, PARK7, NFKB1, CAT, BCL2, BIRC5, BCL2A1D, XDH, BCL2A1B | 108 | 798 | 17446 | 3.036410471 | 0.120107 | 0.006119 | 0.0054529 |
| GOTERM_MF_DIRECT | GO:0043546~molybdopterin cofactor binding | 3 | 2.68 | 3.68E-04 | AOX1, SUOX, XDH | 108 | 5 | 17446 | 96.92222222 | 0.12061 | 0.006119 | 0.0054529 |
| GOTERM_BP_DIRECT | GO:0000303~response to superoxide | 3 | 2.68 | 3.69E-04 | UCP3, SOD2, SOD1 | 112 | 5 | 18082 | 96.86785714 | 0.409626 | 0.009085 | 0.0085247 |
| GOTERM_BP_DIRECT | GO:0032355~response to estradiol | 6 | 5.36 | 3.95E-04 | CASP9, CASP8, TH, CAT, PTEN, BID | 112 | 101 | 18082 | 9.590876945 | 0.431066 | 0.009557 | 0.0089683 |
| GOTERM_MF_DIRECT | GO:0008198~ferrous iron binding | 4 | 3.57 | 4.13E-04 | TH, ALKBH6, ALKBH8, SNCA | 108 | 24 | 17446 | 26.92283951 | 0.134359 | 0.006557 | 0.0058431 |
| GOTERM_BP_DIRECT | GO:0070301~cellular response to hydrogen peroxide | 5 | 4.46 | 4.14E-04 | PRKAA1, MDM2, PARK7, AQP1, NFE2L2 | 112 | 57 | 18082 | 14.16196742 | 0.446046 | 0.009843 | 0.009236 |
| GOTERM_CC_DIRECT | GO:0097342~ripoptosome | 3 | 2.68 | 4.58E-04 | CASP8, RIPK3, RIPK1 | 111 | 6 | 19662 | 88.56756757 | 0.093888 | 0.007582 | 0.006877 |
| GOTERM_MF_DIRECT | GO:0043027~cysteine-type endopeptidase inhibitor activity involved in apoptotic process | 4 | 3.57 | 4.68E-04 | XIAP, BIRC5, BCL2A1D, SNCA | 108 | 25 | 17446 | 25.84592593 | 0.150609 | 0.007096 | 0.006323 |
| GOTERM_BP_DIRECT | GO:2000377~regulation of reactive oxygen species metabolic process | 4 | 3.57 | 4.69E-04 | PINK1, RIPK3, RIPK1, SNCA | 112 | 25 | 18082 | 25.83142857 | 0.488427 | 0.010985 | 0.0103084 |
| GOTERM_BP_DIRECT | GO:0051091~positive regulation of sequence-specific DNA binding transcription factor activity | 6 | 5.36 | 5.15E-04 | PINK1, PTEN, AKT1, TRAF2, PARK7, PHB2 | 112 | 107 | 18082 | 9.053070761 | 0.520632 | 0.011856 | 0.0111258 |
| GOTERM_BP_DIRECT | GO:0008637~apoptotic mitochondrial changes | 4 | 3.57 | 5.91E-04 | BCL2, AKT1, SOD2, BID | 112 | 27 | 18082 | 23.91798942 | 0.570389 | 0.013407 | 0.0125806 |
| GOTERM_CC_DIRECT | GO:0031931~TORC1 complex | 3 | 2.68 | 6.40E-04 | AKT1S1, MLST8, MTOR | 111 | 7 | 19662 | 75.91505792 | 0.128499 | 0.009821 | 0.0089074 |
| GOTERM_BP_DIRECT | GO:0010033~response to organic substance | 5 | 4.46 | 6.83E-04 | SOX2, CASP3, AQP9, AKT1, SOD1 | 112 | 65 | 18082 | 12.41895604 | 0.622863 | 0.014997 | 0.014073 |
| GOTERM_BP_DIRECT | GO:0032436~positive regulation of proteasomal ubiquitin-dependent protein catabolic process | 5 | 4.46 | 6.83E-04 | SUMO1, LRRK2, SUMO2, MDM2, AKT1 | 112 | 65 | 18082 | 12.41895604 | 0.622863 | 0.014997 | 0.014073 |
| GOTERM_MF_DIRECT | GO:0016174~NAD(P)H oxidase activity | 3 | 2.68 | 7.67E-04 | DUOX1, NOX4, DUOX2 | 108 | 7 | 17446 | 69.23015873 | 0.234939 | 0.010308 | 0.0091854 |
| GOTERM_MF_DIRECT | GO:0047485~protein N-terminus binding | 6 | 5.36 | 7.68E-04 | NFE2, PARP1, ACOX1, ATM, PHB2, SNCA | 108 | 117 | 17446 | 8.283950617 | 0.235173 | 0.010308 | 0.0091854 |
| GOTERM_BP_DIRECT | GO:0036289~peptidyl-serine autophosphorylation | 3 | 2.68 | 7.69E-04 | PINK1, ATM, RIPK1 | 112 | 7 | 18082 | 69.19132653 | 0.666481 | 0.016631 | 0.0156059 |
| GOTERM_BP_DIRECT | GO:0007569~cell aging | 4 | 3.57 | 8.10E-04 | BCL2, NOX4, MTOR, SOD1 | 112 | 30 | 18082 | 21.52619048 | 0.685652 | 0.017012 | 0.0159632 |
| GOTERM_BP_DIRECT | GO:0012501~programmed cell death | 4 | 3.57 | 8.93E-04 | RIPK3, CASP4, RIPK1, ALKBH7 | 112 | 31 | 18082 | 20.83179724 | 0.720736 | 0.018215 | 0.0170922 |
| GOTERM_BP_DIRECT | GO:0007249~I-kappaB kinase/NF-kappaB signaling | 4 | 3.57 | 9.81E-04 | RIPK3, RIPK2, NFKB1, NFKB2 | 112 | 32 | 18082 | 20.18080357 | 0.753737 | 0.019454 | 0.0182549 |
| GOTERM_BP_DIRECT | GO:0032287~peripheral nervous system myelin maintenance | 3 | 2.68 | 0.001021 | AKT2, AKT1, SOD1 | 112 | 8 | 18082 | 60.54241071 | 0.767389 | 0.019968 | 0.0187372 |
| GOTERM_BP_DIRECT | GO:0043523~regulation of neuron apoptotic process | 4 | 3.57 | 0.001074 | PINK1, AKT1S1, PARK7, SNCA | 112 | 33 | 18082 | 19.56926407 | 0.784482 | 0.020728 | 0.0194508 |
| GOTERM_BP_DIRECT | GO:0045944~positive regulation of transcription from RNA polymerase II promoter | 16 | 14.29 | 0.001107 | PARP1, CTBP2, PARK7, FOXO4, BRCA1, HIF1A, NFKB1, NFKB2, SOX2, SOX1, AKT2, SUMO2, AKT1, RIPK1, NFE2L1, NFE2L2 | 112 | 995 | 18082 | 2.596123475 | 0.794396 | 0.021079 | 0.01978 |
| GOTERM_BP_DIRECT | GO:0007507~heart development | 8 | 7.14 | 0.001143 | CASP7, CASP8, TH, CASP3, PTEN, MDM2, ATM, SOD2 | 112 | 261 | 18082 | 4.948549535 | 0.804665 | 0.021201 | 0.0198941 |
| GOTERM_BP_DIRECT | GO:0050821~protein stabilization | 6 | 5.36 | 0.001157 | PINK1, SUMO1, PTEN, PARK7, GAPDH, PHB2 | 112 | 128 | 18082 | 7.567801339 | 0.80862 | 0.021201 | 0.0198941 |
| GOTERM_BP_DIRECT | GO:0010332~response to gamma radiation | 4 | 3.57 | 0.001173 | PRKAA1, PARP1, BCL2, SOD2 | 112 | 34 | 18082 | 18.99369748 | 0.812847 | 0.021201 | 0.0198941 |
| GOTERM_BP_DIRECT | GO:0035067~negative regulation of histone acetylation | 3 | 2.68 | 0.001307 | CTBP1, BRCA1, SNCA | 112 | 9 | 18082 | 53.81547619 | 0.84554 | 0.022489 | 0.0211033 |
| GOTERM_BP_DIRECT | GO:0042743~hydrogen peroxide metabolic process | 3 | 2.68 | 0.001307 | PARK7, SOD2, NOX1 | 112 | 9 | 18082 | 53.81547619 | 0.84554 | 0.022489 | 0.0211033 |
| GOTERM_BP_DIRECT | GO:0006977~DNA damage response, signal transduction by p53 class mediator resulting in cell cycle arrest | 3 | 2.68 | 0.001307 | PIDD1, MDM2, CASP2 | 112 | 9 | 18082 | 53.81547619 | 0.84554 | 0.022489 | 0.0211033 |
| GOTERM_MF_DIRECT | GO:0019901~protein kinase binding | 10 | 8.93 | 0.001402 | CASP9, PARP1, PTEN, AKT1, VDAC1, TRAF2, CDC25C, HIF1A, CDC25A, MTOR | 108 | 434 | 17446 | 3.722051545 | 0.387131 | 0.017474 | 0.015571 |
| GOTERM_CC_DIRECT | GO:0035631~CD40 receptor complex | 3 | 2.68 | 0.001651 | DIABLO, HTRA2, TRAF2 | 111 | 11 | 19662 | 48.30958231 | 0.298942 | 0.022179 | 0.0201162 |
| GOTERM_MF_DIRECT | GO:0050661~NADP binding | 4 | 3.57 | 0.001746 | NNT, CAT, GAPDHS, GAPDH | 108 | 39 | 17446 | 16.56790123 | 0.456562 | 0.021011 | 0.0187229 |
| GOTERM_BP_DIRECT | GO:2001235~positive regulation of apoptotic signaling pathway | 4 | 3.57 | 0.001752 | PIDD1, PTEN, CASP2, BID | 112 | 39 | 18082 | 16.55860806 | 0.918225 | 0.02943 | 0.0276168 |
| GOTERM_BP_DIRECT | GO:0006914~autophagy | 6 | 5.36 | 0.001832 | PRKAA1, PINK1, LRRK2, ULK2, PARK7, MTOR | 112 | 142 | 18082 | 6.82168008 | 0.927057 | 0.030415 | 0.0285406 |
| GOTERM_CC_DIRECT | GO:0031932~TORC2 complex | 3 | 2.68 | 0.001973 | PINK1, MLST8, MTOR | 111 | 12 | 19662 | 44.28378378 | 0.346041 | 0.024958 | 0.0226366 |
| GOTERM_BP_DIRECT | GO:1903206~negative regulation of hydrogen peroxide-induced cell death | 3 | 2.68 | 0.002368 | LRRK2, PARK7, NFE2L2 | 112 | 12 | 18082 | 40.36160714 | 0.966128 | 0.038864 | 0.0364689 |
| GOTERM_CC_DIRECT | GO:0043005~neuron projection | 9 | 8.04 | 0.002495 | CASP8, TH, LRRK2, CASP4, PTEN, PARK7, NFKB1, AQP1, SOD1 | 111 | 420 | 19662 | 3.795752896 | 0.415545 | 0.02684 | 0.0243431 |
| GOTERM_CC_DIRECT | GO:0031966~mitochondrial membrane | 5 | 4.46 | 0.002497 | LRRK2, UCP3, UCP1, BCL2, HTRA2 | 111 | 101 | 19662 | 8.769066096 | 0.415774 | 0.02684 | 0.0243431 |
| GOTERM_BP_DIRECT | GO:0034644~cellular response to UV | 4 | 3.57 | 0.002821 | CASP9, CDC25A, AQP1, ATR | 112 | 46 | 18082 | 14.03881988 | 0.982289 | 0.044754 | 0.0419964 |
| GOTERM_BP_DIRECT | GO:0010506~regulation of autophagy | 4 | 3.57 | 0.002821 | LRRK2, BCL2, ULK2, ATM | 112 | 46 | 18082 | 14.03881988 | 0.982289 | 0.044754 | 0.0419964 |
| GOTERM_BP_DIRECT | GO:0010628~positive regulation of gene expression | 9 | 8.04 | 0.003209 | PRKAA1, AKT2, MDM2, PARK7, BRCA1, HIF1A, NFKB1, MTOR, NFE2L2 | 112 | 399 | 18082 | 3.641648765 | 0.989839 | 0.049803 | 0.0467335 |
| GOTERM_BP_DIRECT | GO:0001963~synaptic transmission, dopaminergic | 3 | 2.68 | 0.004237 | TH, PARK7, SNCA | 112 | 16 | 18082 | 30.27120536 | 0.997672 | 0.063019 | 0.0591357 |
| GOTERM_BP_DIRECT | GO:0008631~intrinsic apoptotic signaling pathway in response to oxidative stress | 3 | 2.68 | 0.004237 | DIABLO, BCL2, SOD2 | 112 | 16 | 18082 | 30.27120536 | 0.997672 | 0.063019 | 0.0591357 |
| GOTERM_BP_DIRECT | GO:0043281~regulation of cysteine-type endopeptidase activity involved in apoptotic process | 3 | 2.68 | 0.004237 | SOX2, RIPK2, MGMT | 112 | 16 | 18082 | 30.27120536 | 0.997672 | 0.063019 | 0.0591357 |
| GOTERM_MF_DIRECT | GO:0046982~protein heterodimerization activity | 10 | 8.93 | 0.00435 | SOX2, AOC3, CASP8, BCL2, BCL2A1D, BID, HIF1A, BCL2A1B, NFKB1, NFE2L2 | 108 | 514 | 17446 | 3.142743911 | 0.781624 | 0.040125 | 0.0357559 |
| GOTERM_CC_DIRECT | GO:0030659~cytoplasmic vesicle membrane | 5 | 4.46 | 0.004633 | TH, AQP6, ULK2, AQP2, SNCA | 111 | 120 | 19662 | 7.380630631 | 0.631547 | 0.045279 | 0.0410666 |
| GOTERM_MF_DIRECT | GO:0003684~damaged DNA binding | 4 | 3.57 | 0.004669 | DDB1, MGMT, BRCA1, DDB2 | 108 | 55 | 17446 | 11.74814815 | 0.804703 | 0.04178 | 0.0372308 |
| GOTERM_CC_DIRECT | GO:0016234~inclusion body | 3 | 2.68 | 0.004984 | RAD50, LRRK2, SNCA | 111 | 19 | 19662 | 27.96870555 | 0.658449 | 0.04659 | 0.0422564 |
| GOTERM_MF_DIRECT | GO:0008134~transcription factor binding | 8 | 7.14 | 0.00512 | SOX2, PARP1, SUMO1, CTBP1, BCL2, PARK7, FOXO4, HIF1A | 108 | 342 | 17446 | 3.778644141 | 0.833286 | 0.044672 | 0.0398081 |
| GOTERM_BP_DIRECT | GO:0009791~post-embryonic development | 5 | 4.46 | 0.005349 | SIAH1A, BCL2, ATM, SOD2, MTOR | 112 | 114 | 18082 | 7.080983709 | 0.999528 | 0.07875 | 0.0738968 |
| GOTERM_BP_DIRECT | GO:0048666~neuron development | 4 | 3.57 | 0.005436 | SOX3, TDP2, HTRA2, SOD2 | 112 | 58 | 18082 | 11.13423645 | 0.999584 | 0.079217 | 0.0743354 |
| GOTERM_MF_DIRECT | GO:0002020~protease binding | 5 | 4.46 | 0.005834 | CASP8AP2, PINK1, CASP3, BCL2, XIAP | 108 | 117 | 17446 | 6.903292181 | 0.870255 | 0.048481 | 0.0432027 |
| GOTERM_BP_DIRECT | GO:0010039~response to iron ion | 3 | 2.68 | 0.005965 | CASP6, BCL2, MDM2 | 112 | 19 | 18082 | 25.49154135 | 0.999805 | 0.084493 | 0.0792863 |
| GOTERM_BP_DIRECT | GO:0031398~positive regulation of protein ubiquitination | 4 | 3.57 | 0.005976 | RIPK2, LRRK2, XIAP, BRCA1 | 112 | 60 | 18082 | 10.76309524 | 0.999808 | 0.084493 | 0.0792863 |
| GOTERM_CC_DIRECT | GO:0005743~mitochondrial inner membrane | 8 | 7.14 | 0.006096 | PINK1, NNT, LRRK2, UCP3, UCP1, VDAC1, SOD2, PHB2 | 111 | 387 | 19662 | 3.66170822 | 0.731447 | 0.054612 | 0.0495321 |
| GOTERM_BP_DIRECT | GO:0006302~double-strand break repair | 4 | 3.57 | 0.006844 | RAD50, PARP1, TDP2, BRCA1 | 112 | 63 | 18082 | 10.25056689 | 0.999945 | 0.095811 | 0.0899063 |
| GOTERM_BP_DIRECT | GO:0032496~response to lipopolysaccharide | 6 | 5.36 | 0.007371 | CASP9, CASP8, TH, SOD2, NFKB2, SNCA | 112 | 197 | 18082 | 4.917150109 | 0.999974 | 0.102187 | 0.09589 |
| GOTERM_BP_DIRECT | GO:0045454~cell redox homeostasis | 4 | 3.57 | 0.007461 | NNT, QSOX1, QSOX2, NFE2L2 | 112 | 65 | 18082 | 9.935164835 | 0.999977 | 0.102451 | 0.0961372 |
| GOTERM_BP_DIRECT | GO:1900182~positive regulation of protein localization to nucleus | 3 | 2.68 | 0.008686 | PARP1, AKT1, PARK7 | 112 | 23 | 18082 | 21.05822981 | 0.999996 | 0.117012 | 0.1098015 |
| GOTERM_BP_DIRECT | GO:0045892~negative regulation of transcription, DNA-templated | 10 | 8.93 | 0.009331 | CTBP2, SUMO1, CTBP1, SUMO2, MDM2, BIRC5, BRCA1, NFKBIE, PHB2, NFKB1 | 112 | 579 | 18082 | 2.788366642 | 0.999998 | 0.12232 | 0.1147823 |
| GOTERM_CC_DIRECT | GO:0043231~intracellular membrane-bounded organelle | 11 | 9.82 | 0.009435 | AOC3, CASP7, ACOX1, AKT2, LRRK2, AQP8, CAT, AQP9, QSOX1, GAPDH, SNCA | 111 | 751 | 19662 | 2.594522618 | 0.869743 | 0.081145 | 0.0735962 |
| GOTERM_BP_DIRECT | GO:0097193~intrinsic apoptotic signaling pathway | 3 | 2.68 | 0.009438 | DIABLO, CASP4, HTRA2 | 112 | 24 | 18082 | 20.18080357 | 0.999999 | 0.12232 | 0.1147823 |
| GOTERM_BP_DIRECT | GO:0071480~cellular response to gamma radiation | 3 | 2.68 | 0.009438 | NOX4, ATM, ATR | 112 | 24 | 18082 | 20.18080357 | 0.999999 | 0.12232 | 0.1147823 |
| GOTERM_BP_DIRECT | GO:0010575~positive regulation of vascular endothelial growth factor production | 3 | 2.68 | 0.009438 | BRCA1, HIF1A, NOX1 | 112 | 24 | 18082 | 20.18080357 | 0.999999 | 0.12232 | 0.1147823 |
| GOTERM_BP_DIRECT | GO:0032868~response to insulin | 4 | 3.57 | 0.009508 | AKT2, UCP3, CAT, MTOR | 112 | 71 | 18082 | 9.095573441 | 0.999999 | 0.12232 | 0.1147823 |
| GOTERM_BP_DIRECT | GO:0048593~camera-type eye morphogenesis | 3 | 2.68 | 0.010218 | AQP5, HIF1A, AQP1 | 112 | 25 | 18082 | 19.37357143 | 1 | 0.130274 | 0.1222458 |
| GOTERM_BP_DIRECT | GO:0050790~regulation of catalytic activity | 3 | 2.68 | 0.011025 | BCL2, SOD2, HIF1A | 112 | 26 | 18082 | 18.62843407 | 1 | 0.136553 | 0.1281381 |
| GOTERM_BP_DIRECT | GO:0032007~negative regulation of TOR signaling | 3 | 2.68 | 0.01186 | PRKAA1, AKT1S1, HIF1A | 112 | 27 | 18082 | 17.93849206 | 1 | 0.136553 | 0.1281381 |
| GOTERM_BP_DIRECT | GO:1901216~positive regulation of neuron death | 3 | 2.68 | 0.01186 | PARP1, ATM, MTOR | 112 | 27 | 18082 | 17.93849206 | 1 | 0.136553 | 0.1281381 |
| GOTERM_BP_DIRECT | GO:0035902~response to immobilization stress | 3 | 2.68 | 0.01186 | AOC3, TH, SOD2 | 112 | 27 | 18082 | 17.93849206 | 1 | 0.136553 | 0.1281381 |
| GOTERM_MF_DIRECT | GO:0051213~dioxygenase activity | 4 | 3.57 | 0.013096 | ALKBH5, ALKBH4, ALKBH7, ALKBH6 | 108 | 80 | 17446 | 8.076851852 | 0.989957 | 0.084642 | 0.0754259 |
| GOTERM_BP_DIRECT | GO:0060548~negative regulation of cell death | 4 | 3.57 | 0.013137 | MGMT, HTRA2, PARK7, NFE2L2 | 112 | 80 | 18082 | 8.072321429 | 1 | 0.144302 | 0.1354097 |
| GOTERM_BP_DIRECT | GO:0034097~response to cytokine | 4 | 3.57 | 0.013582 | CASP6, BCL2, NFKB1, NFKB2 | 112 | 81 | 18082 | 7.972663139 | 1 | 0.146121 | 0.1371165 |
| GOTERM_BP_DIRECT | GO:0048738~cardiac muscle tissue development | 3 | 2.68 | 0.013609 | CASP8, PTEN, MTOR | 112 | 29 | 18082 | 16.70135468 | 1 | 0.146121 | 0.1371165 |
| GOTERM_BP_DIRECT | GO:0042149~cellular response to glucose starvation | 3 | 2.68 | 0.014524 | PRKAA1, BCL2, NFE2L2 | 112 | 30 | 18082 | 16.14464286 | 1 | 0.153627 | 0.1441597 |
| GOTERM_BP_DIRECT | GO:0048535~lymph node development | 3 | 2.68 | 0.014524 | RIPK3, NFKB1, NFKB2 | 112 | 30 | 18082 | 16.14464286 | 1 | 0.153627 | 0.1441597 |
| GOTERM_MF_DIRECT | GO:0004601~peroxidase activity | 3 | 2.68 | 0.015434 | DUOX1, CAT, DUOX2 | 108 | 31 | 17446 | 15.63261649 | 0.995609 | 0.094496 | 0.0842074 |
| GOTERM_BP_DIRECT | GO:0043029~T cell homeostasis | 3 | 2.68 | 0.015464 | RIPK3, CASP3, BCL2 | 112 | 31 | 18082 | 15.62384793 | 1 | 0.162368 | 0.152362 |
| GOTERM_BP_DIRECT | GO:0046326~positive regulation of glucose import | 3 | 2.68 | 0.01742 | AKT2, AKT1, NFE2L2 | 112 | 33 | 18082 | 14.67694805 | 1 | 0.180257 | 0.1691484 |
| GOTERM_MF_DIRECT | GO:0001205~transcriptional activator activity, RNA polymerase II distal enhancer sequence-specific binding | 3 | 2.68 | 0.0184 | NFKB1, NFE2L1, NFE2L2 | 108 | 34 | 17446 | 14.25326797 | 0.998468 | 0.101928 | 0.09083 |
| GOTERM_BP_DIRECT | GO:0008625~extrinsic apoptotic signaling pathway via death domain receptors | 3 | 2.68 | 0.019475 | CASP8AP2, CASP8, BCL2 | 112 | 35 | 18082 | 13.83826531 | 1 | 0.193128 | 0.1812264 |
| GOTERM_BP_DIRECT | GO:0010043~response to zinc ion | 3 | 2.68 | 0.019475 | TH, PTEN, SOD2 | 112 | 35 | 18082 | 13.83826531 | 1 | 0.193128 | 0.1812264 |
| GOTERM_BP_DIRECT | GO:0031648~protein destabilization | 3 | 2.68 | 0.021627 | SIAH1A, MDM2, SNCA | 112 | 37 | 18082 | 13.09025097 | 1 | 0.211529 | 0.1984932 |
| GOTERM_BP_DIRECT | GO:0040014~regulation of multicellular organism growth | 3 | 2.68 | 0.022738 | SIAH1A, HTRA2, SOD1 | 112 | 38 | 18082 | 12.74577068 | 1 | 0.215811 | 0.2025117 |
| GOTERM_BP_DIRECT | GO:0000723~telomere maintenance | 3 | 2.68 | 0.022738 | RAD50, PARP1, ATM | 112 | 38 | 18082 | 12.74577068 | 1 | 0.215811 | 0.2025117 |
| GOTERM_CC_DIRECT | GO:0043209~myelin sheath | 5 | 4.46 | 0.022836 | BCL2, VDAC1, SOD2, GAPDH, SOD1 | 111 | 192 | 19662 | 4.612894144 | 0.993034 | 0.188836 | 0.1712696 |
| GOTERM_BP_DIRECT | GO:0010629~negative regulation of gene expression | 6 | 5.36 | 0.023779 | PINK1, MDM2, AKT1, PARK7, XDH, NFKB1 | 112 | 265 | 18082 | 3.655390836 | 1 | 0.215811 | 0.2025117 |
| GOTERM_BP_DIRECT | GO:0043124~negative regulation of I-kappaB kinase/NF-kappaB signaling | 3 | 2.68 | 0.023873 | CASP8, RIPK1, NFKBID | 112 | 39 | 18082 | 12.41895604 | 1 | 0.215811 | 0.2025117 |
| GOTERM_BP_DIRECT | GO:0014911~positive regulation of smooth muscle cell migration | 3 | 2.68 | 0.023873 | BCL2, NOX4, FOXO4 | 112 | 39 | 18082 | 12.41895604 | 1 | 0.215811 | 0.2025117 |
| GOTERM_MF_DIRECT | GO:0003682~chromatin binding | 8 | 7.14 | 0.024625 | SOX2, PRKAA1, PHC1, PARP1, CTBP2, BRCA1, NFKB1, NFKB2 | 108 | 466 | 17446 | 2.773168018 | 0.999834 | 0.126385 | 0.1126243 |
| GOTERM_BP_DIRECT | GO:0009409~response to cold | 3 | 2.68 | 0.02503 | CASP8, UCP3, SOD2 | 112 | 40 | 18082 | 12.10848214 | 1 | 0.220633 | 0.2070361 |
| GOTERM_BP_DIRECT | GO:0097191~extrinsic apoptotic signaling pathway | 3 | 2.68 | 0.026209 | CASP8, RIPK1, BID | 112 | 41 | 18082 | 11.81315331 | 1 | 0.228214 | 0.2141504 |
| GOTERM_BP_DIRECT | GO:0010508~positive regulation of autophagy | 3 | 2.68 | 0.026209 | PRKAA1, LRRK2, HIF1A | 112 | 41 | 18082 | 11.81315331 | 1 | 0.228214 | 0.2141504 |
| GOTERM_MF_DIRECT | GO:0046872~metal ion binding | 30 | 26.79 | 0.029262 | PRKAA1, XIAP, BRCA1, ALKBH7, ALKBH6, ALKBH8, ALKBH5, ALKBH4, AOX1, XDH, SNCA, AOC3, AOC1, PHC1, AOC2, PARP1, MGMT, SIAH1A, TRAF2, SOD2, SUOX, SOD1, PINK1, RAD50, TH, TDP2, CAT, MDM2, BIRC5, NOX1 | 108 | 3355 | 17446 | 1.444444444 | 0.999968 | 0.142881 | 0.1273235 |
| GOTERM_BP_DIRECT | GO:0071356~cellular response to tumor necrosis factor | 4 | 3.57 | 0.030261 | RIPK1, BRCA1, NFKB1, NFE2L2 | 112 | 110 | 18082 | 5.870779221 | 1 | 0.241901 | 0.2269937 |
| GOTERM_BP_DIRECT | GO:0051726~regulation of cell cycle | 4 | 3.57 | 0.03168 | CTBP1, PTEN, BCL2, ATM | 112 | 112 | 18082 | 5.765943878 | 1 | 0.251327 | 0.2358389 |
| GOTERM_CC_DIRECT | GO:0016604~nuclear body | 3 | 2.68 | 0.031916 | PHC1, SUMO1, MDM2 | 111 | 50 | 19662 | 10.62810811 | 0.999064 | 0.234325 | 0.2125276 |
| GOTERM_BP_DIRECT | GO:0048536~spleen development | 3 | 2.68 | 0.032435 | RIPK3, BCL2, NFKB2 | 112 | 46 | 18082 | 10.52911491 | 1 | 0.255893 | 0.240124 |
| GOTERM_MF_DIRECT | GO:0019904~protein domain specific binding | 6 | 5.36 | 0.032686 | TH, CTBP1, CASP2, MTOR, NFE2L2, SNCA | 108 | 289 | 17446 | 3.353710111 | 0.999991 | 0.150098 | 0.1337552 |
| GOTERM_BP_DIRECT | GO:0030163~protein catabolic process | 3 | 2.68 | 0.033743 | SIAH1A, AKT1, TRAF2 | 112 | 47 | 18082 | 10.30509119 | 1 | 0.264752 | 0.2484366 |
| GOTERM_CC_DIRECT | GO:0000785~chromatin | 4 | 3.57 | 0.033786 | PINK1, HTRA2, PARK7, NFE2L2 | 111 | 126 | 19662 | 5.623337623 | 0.999382 | 0.234325 | 0.2125276 |
| GOTERM_BP_DIRECT | GO:0010942~positive regulation of cell death | 3 | 2.68 | 0.035071 | HTRA2, RIPK1, MTOR | 112 | 48 | 18082 | 10.09040179 | 1 | 0.271219 | 0.2545053 |
| GOTERM_BP_DIRECT | GO:0007049~cell cycle | 9 | 8.04 | 0.035355 | CASP8AP2, RAD50, SIAH1A, BIRC5, ATM, FOXO4, BRCA1, CDC25C, CDC25A | 112 | 614 | 18082 | 2.366478595 | 1 | 0.271219 | 0.2545053 |
| GOTERM_CC_DIRECT | GO:0005769~early endosome | 5 | 4.46 | 0.038209 | AOC3, AKT2, SIAH1A, AQP2, NOX1 | 111 | 226 | 19662 | 3.918918919 | 0.99977 | 0.250278 | 0.2269966 |
| GOTERM_CC_DIRECT | GO:0005764~lysosome | 6 | 5.36 | 0.038415 | LRRK2, CAT, AQP2, MTOR, PCYOX1, SOD1 | 111 | 331 | 19662 | 3.210908794 | 0.99978 | 0.250278 | 0.2269966 |
| GOTERM_BP_DIRECT | GO:0045766~positive regulation of angiogenesis | 4 | 3.57 | 0.038491 | BRCA1, HIF1A, AQP1, NFE2L2 | 112 | 121 | 18082 | 5.337072019 | 1 | 0.286278 | 0.2686362 |
| GOTERM_BP_DIRECT | GO:0008286~insulin receptor signaling pathway | 3 | 2.68 | 0.039176 | AKT2, AKT1, FOXO4 | 112 | 51 | 18082 | 9.496848739 | 1 | 0.289865 | 0.2720023 |
| GOTERM_CC_DIRECT | GO:0048471~perinuclear region of cytoplasm | 9 | 8.04 | 0.040393 | RAD50, PINK1, BCL2, XIAP, NOX4, PARK7, CDC25C, NFKBIE, SNCA | 111 | 692 | 19662 | 2.303780659 | 0.999859 | 0.255428 | 0.2316669 |
| GOTERM_BP_DIRECT | GO:0051865~protein autoubiquitination | 3 | 2.68 | 0.040584 | TRAF2, BRCA1, DDB2 | 112 | 52 | 18082 | 9.314217033 | 1 | 0.298729 | 0.2803195 |
| GOTERM_BP_DIRECT | GO:0035176~social behavior | 3 | 2.68 | 0.043454 | TH, PTEN, MTOR | 112 | 54 | 18082 | 8.969246032 | 1 | 0.314988 | 0.2955772 |
| GOTERM_BP_DIRECT | GO:0048538~thymus development | 3 | 2.68 | 0.044917 | RIPK3, BCL2, ATM | 112 | 55 | 18082 | 8.806168831 | 1 | 0.323949 | 0.3039857 |
| GOTERM_BP_DIRECT | GO:1901215~negative regulation of neuron death | 3 | 2.68 | 0.046398 | LRRK2, PARK7, SNCA | 112 | 56 | 18082 | 8.648915816 | 1 | 0.332949 | 0.3124314 |
| GOTERM_BP_DIRECT | GO:0001541~ovarian follicle development | 3 | 2.68 | 0.047897 | BCL2, ATM, SOD1 | 112 | 57 | 18082 | 8.497180451 | 1 | 0.338193 | 0.3173518 |
| GOTERM_BP_DIRECT | GO:0015992~proton transport | 3 | 2.68 | 0.049414 | NNT, UCP1, NOX1 | 112 | 58 | 18082 | 8.35067734 | 1 | 0.345896 | 0.3245807 |
| GOTERM_BP_DIRECT | GO:0008217~regulation of blood pressure | 3 | 2.68 | 0.05565 | AOC3, SOD2, SOD1 | 112 | 62 | 18082 | 7.811923963 | 1 | 0.373088 | 0.3500967 |
| GOTERM_BP_DIRECT | GO:0061024~membrane organization | 3 | 2.68 | 0.05725 | AKT2, AKT1, SNCA | 112 | 63 | 18082 | 7.68792517 | 1 | 0.382023 | 0.3584807 |
| GOTERM_BP_DIRECT | GO:0071300~cellular response to retinoic acid | 3 | 2.68 | 0.060498 | PHC1, HTRA2, AQP1 | 112 | 65 | 18082 | 7.451373626 | 1 | 0.392687 | 0.3684876 |
| GOTERM_BP_DIRECT | GO:0006954~inflammatory response | 6 | 5.36 | 0.060894 | CASP4, AKT1, PARK7, NFKBID, NFKB2, NFE2L2 | 112 | 344 | 18082 | 2.81592608 | 1 | 0.393468 | 0.3692205 |
| GOTERM_BP_DIRECT | GO:0090263~positive regulation of canonical Wnt signaling pathway | 3 | 2.68 | 0.063808 | LRRK2, XIAP, NFKB1 | 112 | 67 | 18082 | 7.228944563 | 1 | 0.41044 | 0.3851469 |
| GOTERM_CC_DIRECT | GO:0031982~vesicle | 4 | 3.57 | 0.064493 | RIPK2, AKT2, AKT1, GAPDH | 111 | 164 | 19662 | 4.32036915 | 0.999999 | 0.374757 | 0.3398962 |
| GOTERM_BP_DIRECT | GO:0021987~cerebral cortex development | 3 | 2.68 | 0.065485 | SOX2, TH, HIF1A | 112 | 68 | 18082 | 7.122636555 | 1 | 0.412097 | 0.3867015 |
| GOTERM_BP_DIRECT | GO:0008344~adult locomotory behavior | 3 | 2.68 | 0.067178 | HTRA2, PARK7, SNCA | 112 | 69 | 18082 | 7.019409938 | 1 | 0.420745 | 0.3948169 |
| GOTERM_CC_DIRECT | GO:0005902~microvillus | 3 | 2.68 | 0.067667 | AOC3, LRRK2, AQP5 | 111 | 76 | 19662 | 6.992176387 | 1 | 0.382853 | 0.3472387 |
| GOTERM_BP_DIRECT | GO:0031647~regulation of protein stability | 3 | 2.68 | 0.068885 | SUMO1, PTEN, BCL2 | 112 | 70 | 18082 | 6.919132653 | 1 | 0.425702 | 0.3994682 |
| GOTERM_BP_DIRECT | GO:0046330~positive regulation of JNK cascade | 3 | 2.68 | 0.068885 | RIPK2, RIPK1, NOX1 | 112 | 70 | 18082 | 6.919132653 | 1 | 0.425702 | 0.3994682 |
| GOTERM_BP_DIRECT | GO:0043087~regulation of GTPase activity | 3 | 2.68 | 0.070606 | MLST8, MTOR, SOD1 | 112 | 71 | 18082 | 6.82168008 | 1 | 0.425702 | 0.3994682 |
| GOTERM_BP_DIRECT | GO:0016567~protein ubiquitination | 6 | 5.36 | 0.072334 | PINK1, SIAH1A, MDM2, AKT1, BRCA1, NFE2L2 | 112 | 362 | 18082 | 2.675907656 | 1 | 0.430385 | 0.4038623 |
| GOTERM_MF_DIRECT | GO:0000980~RNA polymerase II distal enhancer sequence-specific DNA binding | 3 | 2.68 | 0.073963 | NFKB1, NFE2L1, NFE2L2 | 108 | 73 | 17446 | 6.638508371 | 1 | 0.3073 | 0.2738403 |
| GOTERM_BP_DIRECT | GO:0007584~response to nutrient | 3 | 2.68 | 0.07409 | UCP3, PTEN, SUOX | 112 | 73 | 18082 | 6.634784736 | 1 | 0.439004 | 0.4119505 |
| GOTERM_BP_DIRECT | GO:0006633~fatty acid biosynthetic process | 3 | 2.68 | 0.075852 | PRKAA1, FADS6, BRCA1 | 112 | 74 | 18082 | 6.545125483 | 1 | 0.446706 | 0.4191779 |
| GOTERM_BP_DIRECT | GO:0009749~response to glucose | 3 | 2.68 | 0.083033 | CASP6, CASP3, PTEN | 112 | 78 | 18082 | 6.209478022 | 1 | 0.470519 | 0.4415235 |
| GOTERM_BP_DIRECT | GO:0008285~negative regulation of cell proliferation | 6 | 5.36 | 0.08781 | NFE2, PTEN, BCL2, NOX4, FOXO4, SOD2 | 112 | 384 | 18082 | 2.522600446 | 1 | 0.484479 | 0.454623 |
| GOTERM_BP_DIRECT | GO:0007050~cell cycle arrest | 3 | 2.68 | 0.08855 | SOX2, ATM, FOXO4 | 112 | 81 | 18082 | 5.979497354 | 1 | 0.484479 | 0.454623 |
| GOTERM_CC_DIRECT | GO:0005759~mitochondrial matrix | 4 | 3.57 | 0.088665 | LRRK2, PARK7, BRCA1, ALKBH7 | 111 | 188 | 19662 | 3.768832662 | 1 | 0.476573 | 0.432241 |
| GOTERM_BP_DIRECT | GO:0007613~memory | 3 | 2.68 | 0.092287 | TH, PTEN, FOXO6 | 112 | 83 | 18082 | 5.835413081 | 1 | 0.498268 | 0.4675621 |
| GOTERM_MF_DIRECT | GO:0020037~heme binding | 4 | 3.57 | 0.092777 | DUOX1, CAT, SUOX, DUOX2 | 108 | 175 | 17446 | 3.692275132 | 1 | 0.371933 | 0.3314361 |
| GOTERM_BP_DIRECT | GO:0006310~DNA recombination | 3 | 2.68 | 0.09607 | RAD52, RAD50, BRCA1 | 112 | 85 | 18082 | 5.698109244 | 1 | 0.508102 | 0.4767903 |
| GOTERM_BP_DIRECT | GO:0030900~forebrain development | 3 | 2.68 | 0.097978 | SOX3, SOX1, HTRA2 | 112 | 86 | 18082 | 5.631852159 | 1 | 0.512498 | 0.4809157 |
| GOTERM_BP_DIRECT | GO:0009636~response to toxic substance | 3 | 2.68 | 0.097978 | MGMT, BCL2, MDM2 | 112 | 86 | 18082 | 5.631852159 | 1 | 0.512498 | 0.4809157 |
| GOTERM_BP_DIRECT | GO:0070374~positive regulation of ERK1 and ERK2 cascade | 4 | 3.57 | 0.109222 | RIPK2, PTEN, NOX4, PHB2 | 112 | 188 | 18082 | 3.435030395 | 1 | 0.549617 | 0.5157467 |
| GOTERM_CC_DIRECT | GO:0044297~cell body | 3 | 2.68 | 0.111576 | PINK1, CASP8, PARK7 | 111 | 102 | 19662 | 5.209856916 | 1 | 0.533085 | 0.4834954 |
| GOTERM_BP_DIRECT | GO:0006469~negative regulation of protein kinase activity | 3 | 2.68 | 0.115609 | AKT1S1, AKT1, PARK7 | 112 | 95 | 18082 | 5.098308271 | 1 | 0.572823 | 0.5375233 |
| GOTERM_BP_DIRECT | GO:0051260~protein homooligomerization | 4 | 3.57 | 0.121051 | RIPK3, RIPK1, SOD2, BID | 112 | 197 | 18082 | 3.278100073 | 1 | 0.588117 | 0.5518746 |
| GOTERM_BP_DIRECT | GO:0010468~regulation of gene expression | 5 | 4.46 | 0.121495 | SOX2, NFE2, BCL2, MDM2, HIF1A | 112 | 308 | 18082 | 2.620883581 | 1 | 0.588117 | 0.5518746 |
| GOTERM_MF_DIRECT | GO:0000287~magnesium ion binding | 4 | 3.57 | 0.127537 | PINK1, TDP2, PTEN, SNCA | 108 | 202 | 17446 | 3.198753209 | 1 | 0.483808 | 0.4311295 |
| GOTERM_BP_DIRECT | GO:0050731~positive regulation of peptidyl-tyrosine phosphorylation | 3 | 2.68 | 0.127778 | RIPK2, MLST8, MTOR | 112 | 101 | 18082 | 4.795438472 | 1 | 0.614366 | 0.5765057 |
| GOTERM_CC_DIRECT | GO:0005794~Golgi apparatus | 11 | 9.82 | 0.129211 | AOC3, PIDD1, LRRK2, AKT3, CAT, QSOX1, AQP2, NFKBIE, MTOR, SNCA, ATR | 111 | 1190 | 19662 | 1.637383602 | 1 | 0.583972 | 0.5296492 |
| GOTERM_CC_DIRECT | GO:0031965~nuclear membrane | 4 | 3.57 | 0.133322 | SUMO1, BCL2, GAPDH, AQP1 | 111 | 226 | 19662 | 3.135135135 | 1 | 0.583972 | 0.5296492 |
| GOTERM_BP_DIRECT | GO:0043410~positive regulation of MAPK cascade | 3 | 2.68 | 0.133972 | SOX2, RIPK1, NOX1 | 112 | 104 | 18082 | 4.657108516 | 1 | 0.639839 | 0.6004087 |
| GOTERM_MF_DIRECT | GO:0005506~iron ion binding | 4 | 3.57 | 0.141445 | TH, AOX1, XDH, ALKBH8 | 108 | 212 | 17446 | 3.047868623 | 1 | 0.51421 | 0.4582215 |
| GOTERM_BP_DIRECT | GO:0009887~organ morphogenesis | 3 | 2.68 | 0.144442 | SOX3, TH, BCL2 | 112 | 109 | 18082 | 4.443479685 | 1 | 0.671865 | 0.6304619 |
| GOTERM_BP_DIRECT | GO:0007420~brain development | 4 | 3.57 | 0.14746 | CASP2, ATM, BID, MTOR | 112 | 216 | 18082 | 2.989748677 | 1 | 0.673227 | 0.6317396 |
| GOTERM_BP_DIRECT | GO:0006629~lipid metabolic process | 6 | 5.36 | 0.152093 | PRKAA1, FADS6, ACOX1, PTEN, BRCA1, ACOX3 | 112 | 459 | 18082 | 2.110410831 | 1 | 0.689488 | 0.6469986 |
| GOTERM_BP_DIRECT | GO:0001649~osteoblast differentiation | 3 | 2.68 | 0.152938 | SOX2, CAT, AKT1 | 112 | 113 | 18082 | 4.286188369 | 1 | 0.690449 | 0.6479006 |
| GOTERM_CC_DIRECT | GO:0031410~cytoplasmic vesicle | 7 | 6.25 | 0.154034 | TH, LRRK2, AQP6, ULK2, ATM, AQP2, SOD1 | 111 | 646 | 19662 | 1.919420969 | 1 | 0.64936 | 0.5889546 |
| GOTERM_CC_DIRECT | GO:0000784~nuclear chromosome, telomeric region | 3 | 2.68 | 0.158844 | DDB1, RAD50, PARP1 | 111 | 127 | 19662 | 4.184294531 | 1 | 0.656758 | 0.5956646 |
| GOTERM_MF_DIRECT | GO:0030165~PDZ domain binding | 3 | 2.68 | 0.159137 | ACOX1, PTEN, AQP2 | 108 | 116 | 17446 | 4.177681992 | 1 | 0.544499 | 0.4852129 |
| GOTERM_MF_DIRECT | GO:0044325~ion channel binding | 3 | 2.68 | 0.167785 | SUMO1, LRRK2, VDAC1 | 108 | 120 | 17446 | 4.038425926 | 1 | 0.560595 | 0.4995561 |
| GOTERM_BP_DIRECT | GO:0035264~multicellular organism growth | 3 | 2.68 | 0.168031 | ATM, DUOX2, MTOR | 112 | 120 | 18082 | 4.036160714 | 1 | 0.732637 | 0.6874884 |
| GOTERM_BP_DIRECT | GO:0001525~angiogenesis | 4 | 3.57 | 0.181646 | CASP8, PTEN, HIF1A, NOX1 | 112 | 239 | 18082 | 2.702032277 | 1 | 0.767427 | 0.7201343 |
| GOTERM_CC_DIRECT | GO:0005667~transcription factor complex | 4 | 3.57 | 0.188342 | SOX2, PARP1, CTBP1, HIF1A | 111 | 267 | 19662 | 2.65370989 | 1 | 0.74007 | 0.6712258 |
| GOTERM_CC_DIRECT | GO:0016020~membrane | 45 | 40.18 | 0.191925 | LRRK2, AQP8, AQP9, UCP1, AQP6, HTRA2, PARK7, AQP5, AQP2, PHB2, AQP3, AQP1, SUMO1, NNT, FADS6, AKT2, UCP3, CASP4, AKT3, AKT1, CASP2, QSOX1, RIPK1, QSOX2, BID, SNCA, AOC3, RIPK3, PARP1, MGMT, RIPK4, MTOR, PINK1, RAD50, ACOX1, CAT, BCL2, PCYOX1L, ULK2, NOX4, VDAC1, ACOX3, GAPDH, NFE2L1, NOX1 | 111 | 6998 | 19662 | 1.139051312 | 1 | 0.74007 | 0.6712258 |
| GOTERM_CC_DIRECT | GO:0005938~cell cortex | 3 | 2.68 | 0.192762 | AKT2, TRAF2, SNCA | 111 | 144 | 19662 | 3.690315315 | 1 | 0.74007 | 0.6712258 |
| GOTERM_BP_DIRECT | GO:0042593~glucose homeostasis | 3 | 2.68 | 0.19667 | PRKAA1, AKT1, HIF1A | 112 | 133 | 18082 | 3.641648765 | 1 | 0.811692 | 0.7616717 |
| GOTERM_BP_DIRECT | GO:0042787~protein ubiquitination involved in ubiquitin-dependent protein catabolic process | 3 | 2.68 | 0.1989 | DDB1, SIAH1A, MDM2 | 112 | 134 | 18082 | 3.614472281 | 1 | 0.811905 | 0.7618719 |
| GOTERM_CC_DIRECT | GO:0005783~endoplasmic reticulum | 11 | 9.82 | 0.205535 | AOC3, LRRK2, CAT, CASP4, BCL2, NOX4, QSOX1, AQP5, PARK7, NFE2L1, MTOR | 111 | 1323 | 19662 | 1.472778901 | 1 | 0.775263 | 0.7031451 |
| GOTERM_CC_DIRECT | GO:0015630~microtubule cytoskeleton | 3 | 2.68 | 0.221344 | AKT1, GAPDH, ALKBH8 | 111 | 158 | 19662 | 3.363325351 | 1 | 0.787162 | 0.7139374 |
| GOTERM_MF_DIRECT | GO:0001077~transcriptional activator activity, RNA polymerase II core promoter proximal region sequence-specific binding | 4 | 3.57 | 0.230197 | SOX2, SOX1, HIF1A, NFKB2 | 108 | 270 | 17446 | 2.393141289 | 1 | 0.704725 | 0.627993 |
| GOTERM_BP_DIRECT | GO:0043161~proteasome-mediated ubiquitin-dependent protein catabolic process | 3 | 2.68 | 0.232649 | DDB1, SIAH1A, NFE2L2 | 112 | 149 | 18082 | 3.250599233 | 1 | 0.90278 | 0.8471463 |
| GOTERM_CC_DIRECT | GO:0070062~extracellular exosome | 19 | 16.96 | 0.23412 | AOC1, LRRK2, PARK7, AQP5, SOD2, AQP2, PHB2, SOD1, AQP1, DDB1, CAT, PNPO, AOX1, QSOX1, VDAC1, BID, GAPDH, DUOX2, PCYOX1 | 111 | 2674 | 19662 | 1.258626615 | 1 | 0.79898 | 0.724656 |
| GOTERM_BP_DIRECT | GO:0007283~spermatogenesis | 5 | 4.46 | 0.240506 | ALKBH5, ACOX1, SIAH1A, CDC25C, SOD1 | 112 | 407 | 18082 | 1.983371358 | 1 | 0.923234 | 0.8663398 |
| GOTERM_MF_DIRECT | GO:0003714~transcription corepressor activity | 3 | 2.68 | 0.250483 | CASP8AP2, CTBP2, CTBP1 | 108 | 157 | 17446 | 3.086694975 | 1 | 0.743506 | 0.6625509 |
| GOTERM_CC_DIRECT | GO:0005694~chromosome | 4 | 3.57 | 0.302644 | RAD50, BIRC5, BRCA1, ATR | 111 | 344 | 19662 | 2.059710874 | 1 | 0.95269 | 0.864068 |
| GOTERM_CC_DIRECT | GO:0005886~plasma membrane | 31 | 27.68 | 0.305747 | LRRK2, AQP8, PTEN, BRCA1, PARK7, AQP5, AQP2, AQP3, AQP1, DUOX1, CASP8, SUMO1, AKT2, AKT3, AKT1, RIPK1, SNCA, AOC3, AOC1, RIPK3, SIAH1A, SOD1, ACOX1, CAT, MDM2, NOX4, VDAC1, GAPDH, NOX1, PCYOX1, NFE2L2 | 111 | 4874 | 19662 | 1.126628886 | 1 | 0.95269 | 0.864068 |
| GOTERM_CC_DIRECT | GO:0045202~synapse | 5 | 4.46 | 0.313033 | CTBP2, SUMO1, LRRK2, MDM2, SNCA | 111 | 505 | 19662 | 1.753813219 | 1 | 0.959852 | 0.8705632 |
| GOTERM_BP_DIRECT | GO:0008152~metabolic process | 5 | 4.46 | 0.316868 | CTBP2, ACOX1, CTBP1, ACOX3, ALKBH8 | 112 | 463 | 18082 | 1.74348195 | 1 | 1 | 0.9390329 |
| GOTERM_CC_DIRECT | GO:0016607~nuclear speck | 3 | 2.68 | 0.318513 | ALKBH5, SUMO1, HIF1A | 111 | 205 | 19662 | 2.59222149 | 1 | 0.959852 | 0.8705632 |
| GOTERM_BP_DIRECT | GO:0030154~cell differentiation | 7 | 6.25 | 0.345935 | ALKBH5, CTBP2, CTBP1, LRRK2, SIAH1A, FOXO4, HIF1A | 112 | 780 | 18082 | 1.448878205 | 1 | 1 | 0.9390329 |
| GOTERM_MF_DIRECT | GO:0008017~microtubule binding | 3 | 2.68 | 0.347687 | BIRC5, GAPDH, SNCA | 108 | 200 | 17446 | 2.423055556 | 1 | 0.994613 | 0.8863171 |
| GOTERM_CC_DIRECT | GO:0043025~neuronal cell body | 5 | 4.46 | 0.349886 | TH, LRRK2, CASP4, MTOR, SOD1 | 111 | 534 | 19662 | 1.658568681 | 1 | 1 | 0.911215 |
| GOTERM_BP_DIRECT | GO:0005975~carbohydrate metabolic process | 3 | 2.68 | 0.361395 | AKT2, AKT1, GAPDH | 112 | 206 | 18082 | 2.351161581 | 1 | 1 | 0.9390329 |
| GOTERM_MF_DIRECT | GO:0008022~protein C-terminus binding | 3 | 2.68 | 0.367629 | PRKAA1, SIAH1A, PHB2 | 108 | 209 | 17446 | 2.31871345 | 1 | 1 | 0.8936782 |
| GOTERM_CC_DIRECT | GO:0030054~cell junction | 6 | 5.36 | 0.37449 | CTBP2, LRRK2, NOX4, NOX1, DDB2, SNCA | 111 | 718 | 19662 | 1.480237898 | 1 | 1 | 0.911215 |
| GOTERM_BP_DIRECT | GO:0016055~Wnt signaling pathway | 3 | 2.68 | 0.376814 | DDB1, PRKAA1, XIAP | 112 | 213 | 18082 | 2.27389336 | 1 | 1 | 0.9390329 |
| GOTERM_BP_DIRECT | GO:0042127~regulation of cell proliferation | 3 | 2.68 | 0.407183 | BRCA1, BID, HIF1A | 112 | 227 | 18082 | 2.133653241 | 1 | 1 | 0.9390329 |
| GOTERM_BP_DIRECT | GO:0007399~nervous system development | 4 | 3.57 | 0.408861 | SOX1, PTEN, AKT1, ULK2 | 112 | 377 | 18082 | 1.712959454 | 1 | 1 | 0.9390329 |
| GOTERM_MF_DIRECT | GO:0044212~transcription regulatory region DNA binding | 3 | 2.68 | 0.419577 | SOX2, BRCA1, NFKB1 | 108 | 233 | 17446 | 2.079876013 | 1 | 1 | 0.8936782 |
| GOTERM_BP_DIRECT | GO:0008284~positive regulation of cell proliferation | 5 | 4.46 | 0.426952 | PRKAA1, PTEN, BCL2, HIF1A, NOX1 | 112 | 542 | 18082 | 1.489358197 | 1 | 1 | 0.9390329 |
| GOTERM_MF_DIRECT | GO:0005509~calcium ion binding | 6 | 5.36 | 0.428342 | AOC3, DUOX1, AOC1, MGMT, DUOX2, SNCA | 108 | 699 | 17446 | 1.386584009 | 1 | 1 | 0.8936782 |
| GOTERM_BP_DIRECT | GO:0007275~multicellular organism development | 8 | 7.14 | 0.44602 | SOX2, SOX3, PHC1, AKT2, SIAH1A, AKT1, FOXO4, GAPDH | 112 | 1029 | 18082 | 1.255171456 | 1 | 1 | 0.9390329 |
| GOTERM_BP_DIRECT | GO:0045087~innate immune response | 4 | 3.57 | 0.446382 | RIPK2, CASP4, NFKB1, NFKB2 | 112 | 400 | 18082 | 1.614464286 | 1 | 1 | 0.9390329 |
| GOTERM_CC_DIRECT | GO:0005615~extracellular space | 10 | 8.93 | 0.467456 | DDB1, AOC3, AOC1, LRRK2, CAT, QSOX1, QSOX2, XDH, SNCA, SOD1 | 111 | 1504 | 19662 | 1.177760207 | 1 | 1 | 0.911215 |
| GOTERM_BP_DIRECT | GO:0007067~mitotic nuclear division | 3 | 2.68 | 0.509225 | BIRC5, CDC25C, CDC25A | 112 | 277 | 18082 | 1.748517277 | 1 | 1 | 0.9390329 |
| GOTERM_CC_DIRECT | GO:0030425~dendrite | 4 | 3.57 | 0.519206 | SUMO1, TH, LRRK2, MTOR | 111 | 490 | 19662 | 1.446001103 | 1 | 1 | 0.911215 |
| GOTERM_CC_DIRECT | GO:0030529~intracellular ribonucleoprotein complex | 3 | 2.68 | 0.53685 | LRRK2, BRCA1, GAPDH | 111 | 320 | 19662 | 1.660641892 | 1 | 1 | 0.911215 |
| GOTERM_CC_DIRECT | GO:0005789~endoplasmic reticulum membrane | 5 | 4.46 | 0.565114 | CASP4, BCL2, NOX4, NFE2L1, MTOR | 111 | 710 | 19662 | 1.247430529 | 1 | 1 | 0.911215 |
| GOTERM_MF_DIRECT | GO:0003824~catalytic activity | 4 | 3.57 | 0.566369 | MGMT, AOX1, XDH, ALKBH8 | 108 | 479 | 17446 | 1.348952293 | 1 | 1 | 0.8936782 |
| GOTERM_MF_DIRECT | GO:0003779~actin binding | 3 | 2.68 | 0.61699 | ALKBH4, LRRK2, AQP2 | 108 | 338 | 17446 | 1.433760684 | 1 | 1 | 0.8936782 |
| GOTERM_MF_DIRECT | GO:0000978~RNA polymerase II core promoter proximal region sequence-specific DNA binding | 3 | 2.68 | 0.649808 | SOX1, NFE2L3, NFKB2 | 108 | 359 | 17446 | 1.349891674 | 1 | 1 | 0.8936782 |
| GOTERM_BP_DIRECT | GO:0051301~cell division | 3 | 2.68 | 0.672236 | BIRC5, CDC25C, CDC25A | 112 | 374 | 18082 | 1.295024828 | 1 | 1 | 0.9390329 |
| GOTERM_BP_DIRECT | GO:0002376~immune system process | 3 | 2.68 | 0.684926 | RIPK2, CASP4, ATM | 112 | 383 | 18082 | 1.264593435 | 1 | 1 | 0.9390329 |
| GOTERM_CC_DIRECT | GO:0005730~nucleolus | 5 | 4.46 | 0.698598 | PARP1, SUMO1, TDP2, ACOX1, MDM2 | 111 | 842 | 19662 | 1.051871349 | 1 | 1 | 0.911215 |
| GOTERM_BP_DIRECT | GO:0006412~translation | 3 | 2.68 | 0.709109 | UCP3, UCP1, AKT1 | 112 | 401 | 18082 | 1.207828643 | 1 | 1 | 0.9390329 |
| GOTERM_CC_DIRECT | GO:0005622~intracellular | 8 | 7.14 | 0.800507 | RIPK3, DIABLO, LRRK2, AKT3, BCL2, TRAF2, CDC25C, CDC25A | 111 | 1599 | 19662 | 0.886229569 | 1 | 1 | 0.911215 |
| GOTERM_CC_DIRECT | GO:0005768~endosome | 3 | 2.68 | 0.812207 | AKT2, LRRK2, NOX1 | 111 | 544 | 19662 | 0.976848172 | 1 | 1 | 0.911215 |
| GOTERM_CC_DIRECT | GO:0005856~cytoskeleton | 5 | 4.46 | 0.875984 | PINK1, RIPK2, HTRA2, BIRC5, SNCA | 111 | 1113 | 19662 | 0.795755324 | 1 | 1 | 0.911215 |
| GOTERM_CC_DIRECT | GO:0042995~cell projection | 3 | 2.68 | 0.911822 | LRRK2, PTEN, NOX1 | 111 | 712 | 19662 | 0.746355906 | 1 | 1 | 0.9118218 |
| GOTERM_CC_DIRECT | GO:0005576~extracellular region | 7 | 6.25 | 0.934781 | AOC1, CASP4, PCYOX1L, QSOX1, XDH, SNCA, SOD1 | 111 | 1753 | 19662 | 0.707327978 | 1 | 1 | 0.9347806 |
| GOTERM_MF_DIRECT | GO:0003723~RNA binding | 3 | 2.68 | 0.955446 | PARK7, BRCA1, ALKBH8 | 108 | 780 | 17446 | 0.621296296 | 1 | 1 | 0.9554455 |
| GOTERM_MF_DIRECT | GO:0044822~poly(A) RNA binding | 4 | 3.57 | 0.970426 | ALKBH5, PARP1, SUMO1, SUMO2 | 108 | 1113 | 17446 | 0.580546404 | 1 | 1 | 0.9704255 |
| GOTERM_CC_DIRECT | GO:0016021~integral component of membrane | 26 | 23.21 | 0.998101 | LRRK2, AQP8, AQP9, UCP1, AQP6, HTRA2, AQP5, AQP2, AQP3, AQP1, DUOX1, NNT, FADS6, UCP3, QSOX1, QSOX2, DUOX2, AOC3, SMOX, PINK1, BCL2, NOX4, VDAC1, NFE2L1, NOX1, PCYOX1 | 111 | 6878 | 19662 | 0.669600685 | 1 | 1 | 0.9981009 |
