# Supplementary material for: Preclinical studies and transcriptome analysis in a model of Parkinson’s disease with dopaminergic ZNF746 expression
Source: Mol Neurodegener. 2025 Feb 28;20:24. doi: 10.1186/s13024-025-00814-3 (PMC11871723; doi:10.1186/s13024-025-00814-3)
Supplement: Supplementary file 3 — Additional file 3. [file 13024_2025_814_MOESM3_ESM.docx]

**Supplementary figures and legends**


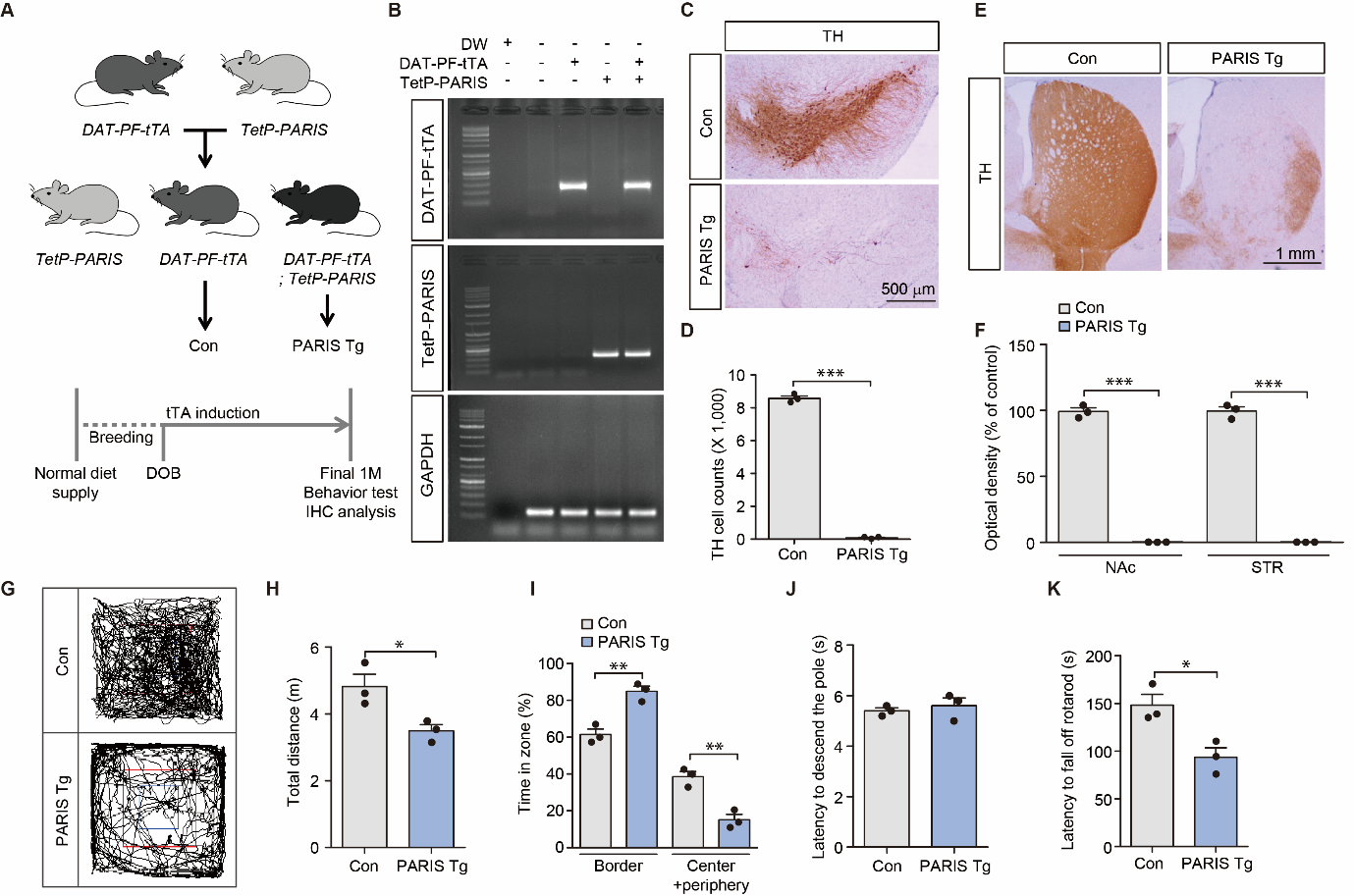


**Fig. S1. DAT-tTA-driven PARIS expression during brain development and dopaminergic deficits.**

(A) Schematic diagram showing the breeding strategy for generation of mice expressing PARIS specifically in the dopaminergic neurons. DAT-PF-tTA mice (Con) were used as a control group, while DAT-PF-tTA:TetP-PARIS mice (PARIS Tg) were used as experimental group. Lower panel indicates experimental schedule to study pathological features of PARIS Tg mice without doxycycline-induced suppression of gene expression.

(B) Representative PCR amplicons of the indicated genotypes: DAT-PF-tTA and TetP-PARIS. GAPDH was used as internal loading control of genomic DNA extracted from mouse tail. DW only without genomic DNA template was used as negative control.

(C) Representative tyrosine hydroxylase (TH) immunohistochemical staining in the ventral midbrains of 1-month-old control or PARIS Tg mice. Scale bar: 500 μm.

(D) Stereological assessment of TH-positive dopaminergic neurons in the substantia nigra of 1-month-old control or PARIS Tg mice (*n* = 3 mice per group).

(E) Representative TH immunohistochemical staining in the striatum (STR) and nucleus accumbens (NAc) of 1-month-old control or PARIS Tg mice. Scale bar: 1 mm.

(F) The graph showing the optical density of TH-positive fibers in the NAc and STR of 1-month-old control or PARIS Tg mice (*n* = 3 mice per group).

(G) Representative tracks of 1-month-old control or PARIS Tg mice in the open field chamber over 15 min.

(H) The graph showing the total distance moved for 15 min of the open-field test of 1-month-old control or PARIS Tg mice (*n* = 3 mice per group).

(I) Relative percentage of time spent in each zone, border, or center with periphery, obtained from open-field test in panel A (*n* = 3 mice per group).

(J) The graph shows the latency of 1-month-old control or PARIS Tg mice to descend the pole (*n* = 3 mice per group).

(K) The graph shows the latency of 1-month-old control or PARIS Tg mice to fall off the rotating-rod (*n* = 3 mice per group).

Data in all panels are mean ± standard error of the mean. **P* <0.05, ***P* <0.01, and ****P* <0.001, unpaired two-tailed student’s *t*-test.

**
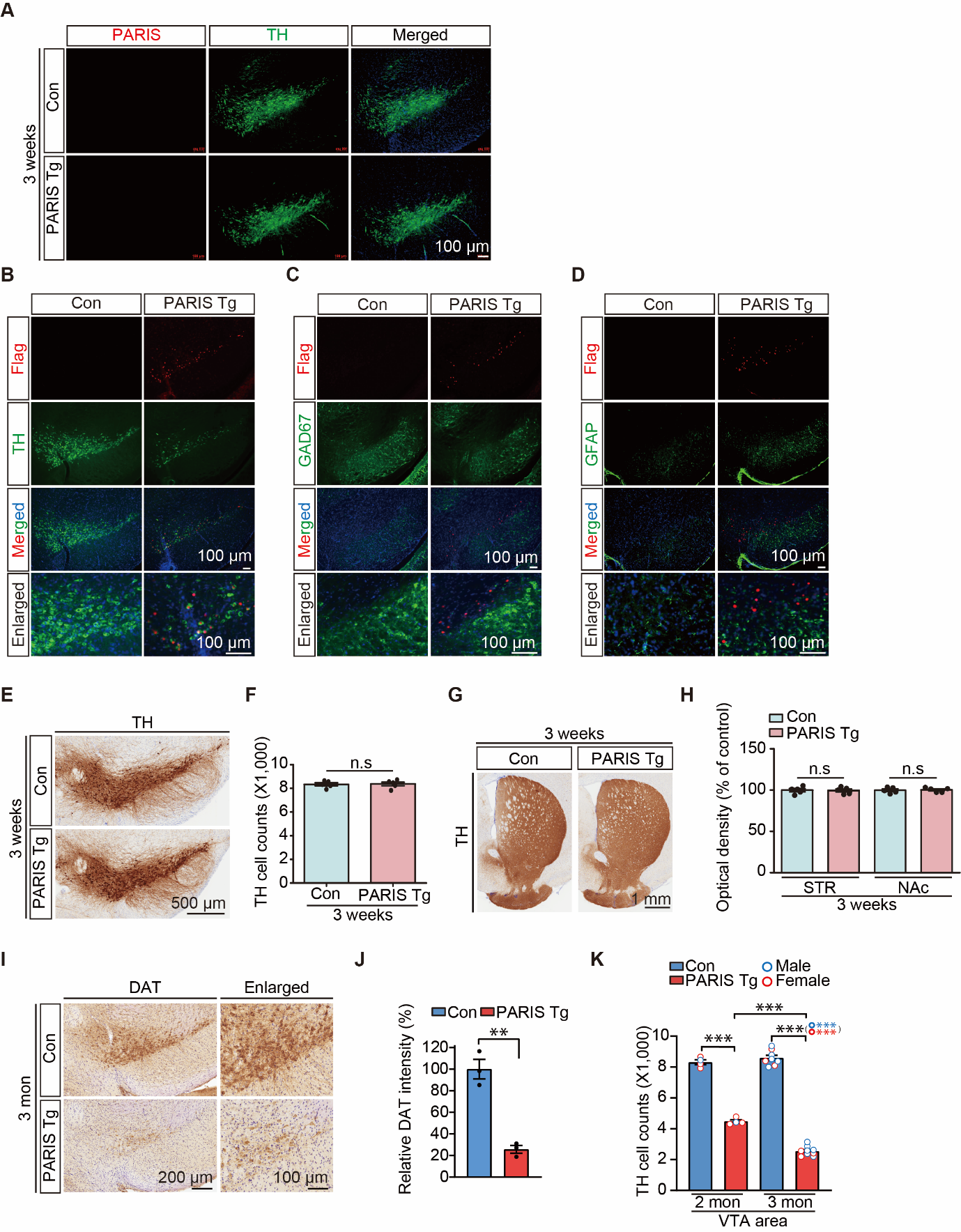
**

**Fig. S2. Tet-off repression of PARIS expression, dopamine neuron selective PARIS induction, and dopamine cell death in the VTA in conditional transgenic mice**

(A) Representative immunofluorescence images showing PARIS gene repression in the TH-positive dopaminergic neurons of PARIS Tg mice before inducing PARIS expression. Scale bar: 100 μm.

(B, C, D) Co-immunofluorescent images of FLAG (PARIS-FLAG, red) with tyrosine hydroxylase (TH, green), or GAD67 (green), or GFAP (green) from ventral midbrain sections of PARIS Tg and control mice. Bottom row, magnified images. Scale bar: 100 μm.

(E) Representative TH immunohistochemical staining for ventral midbrains of control or PARIS Tg mice before inducing PARIS expression. Scale bar: 500 μm.

(F) Stereological assessment of TH-positive dopaminergic neurons in the substantia nigra of control or PARIS Tg mice before inducing PARIS expression (*n* = 5 mice per group).

(G) Representative TH immunohistochemical staining for striatum (STR) and nucleus accumbens (NAc) of control or PARIS Tg mice before inducing PARIS expression. Scale bar: 1 mm.

(H) The graph showing that the optical density of TH-positive fibers in the NAc and STR from panel D (*n* = 5 mice per group).

(I) Representative DAT immunohistochemical staining for the ventral midbrains of 3-month-old control or PARIS transgenic mice. Scale bar: 200 μm for left panel, and 100 μm for enlarged right panel.

(J) The graph showing the optical density of DAT signal intensity in the midbrins from the indicated experimental groups in panel I (*n* = 3 mice per group).

(K) Stereological assessment of TH-positive dopaminergic neurons in the ventral tegmental area (VTA) of 2–3-month-old control or PARIS Tg mice (*n* = 4 per 2-months-old control, and PARIS Tg mice, 8 per 3-month-old control, 9 per 3-month-old PARIS Tg mice). Male and female mice are indicated by blue and red empty circles, respectively.

Data in all panels are mean ± standard error of the mean. ****P* <0.001, Unpaired two-tailed Student’s *t*-test or two-way analysis of variance (ANOVA) test followed by Tukey’s post hoc analysis. n.s, nonsignificant.

**
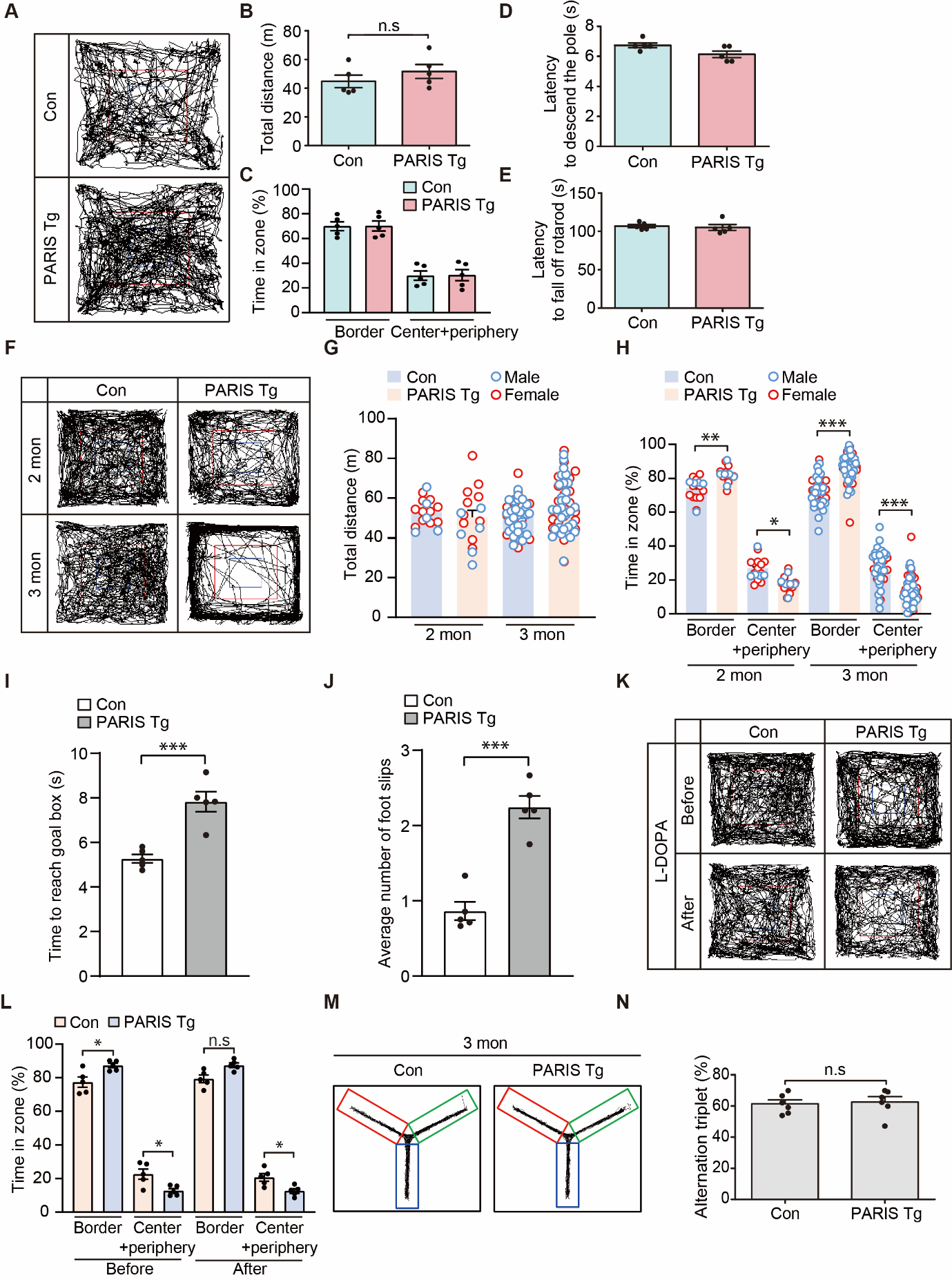
**

**Fig. S3. PARIS Tg mice develops anxiety-like behavior and balance deficits with no impairment of working memory.**

(A) Representative tracks of control or PARIS Tg mice, which repressed PARIS gene expression in dopamine neurons, in the open field chamber over 15 min.

(B) The graph showing that total distance moved for 15 min in the open field chamber obtained from panel A (*n* = 5 mice per group).

(C) Relative percentage of time spent in each zone, border, or center with periphery, obtained from the open field test in panel A (*n* = 5 mice per group).

(D, E) The graphs show the latency to descend the pole (D) and to fall off the rotating-rod (E) of control or PARIS Tg mice, which were repressed PARIS gene expression in dopamine neurons (*n* = 5 mice per group).

(F) Representative tracks of control or PARIS Tg mice at 2 and 3 months of age in the open filed chamber over 15 min.

(G) The graph showing that total distance moved for 15 min in the open field chamber obtained from panel F (*n* = 16 mice for 2-month-old control or PARIS Tg group, 44 mice for 3-month-old control group, 57 mice for 3-month-old PARIS Tg group). Male and female mice are indicated by blue and red empty circles, respectively.

(H) Relative percentage of time spent in each zone, border, or center with periphery, obtained from the open field test in panel F (*n* = 16 mice for 2-month-old control or PARIS Tg group, 44 mice for 3-month-old control group, 57 mice for 3-month-old PARIS Tg group). Male and female mice are indicated by blue and red empty circles, respectively.

(I, J) The beam walking test. The graph shows the time to traverse the narrow beam and number of foot slips during the test by 2-month-old control or PARIS Tg mice (*n* = 5 mice per group). Related movies are in the Supplementary movie 2.

(K) Representative tracks of 2.5-month-old control or PARIS Tg mice before or after administration of carbidopa/levodopa (L-DOPA) in the open field chamber over 15 min.

(L) Relative percentage of time spent in each zone, border, or center with periphery, obtained from the open field test in panel I (*n* = 5 mice per groups).

(M) Representative locomotion tracking of the 3-month-old control or PARIS Tg in the Y-shaped maze with red, green, and blue plastic arms at 120° from each other for 5 min.

(N) The graph shows the comparison of alternation percentage between 3-month-old control and PARIS Tg (*n* = 6 mice per group).

Data in all panels are mean ± standard error of the mean. **P* <0.05, ***P* <0.01, and ****P* <0.001, unpaired two-tailed student’s *t*-test or two-way analysis of variance (ANOVA) test followed by Tukey’s post hoc analysis. n.s, nonsignificant.

**
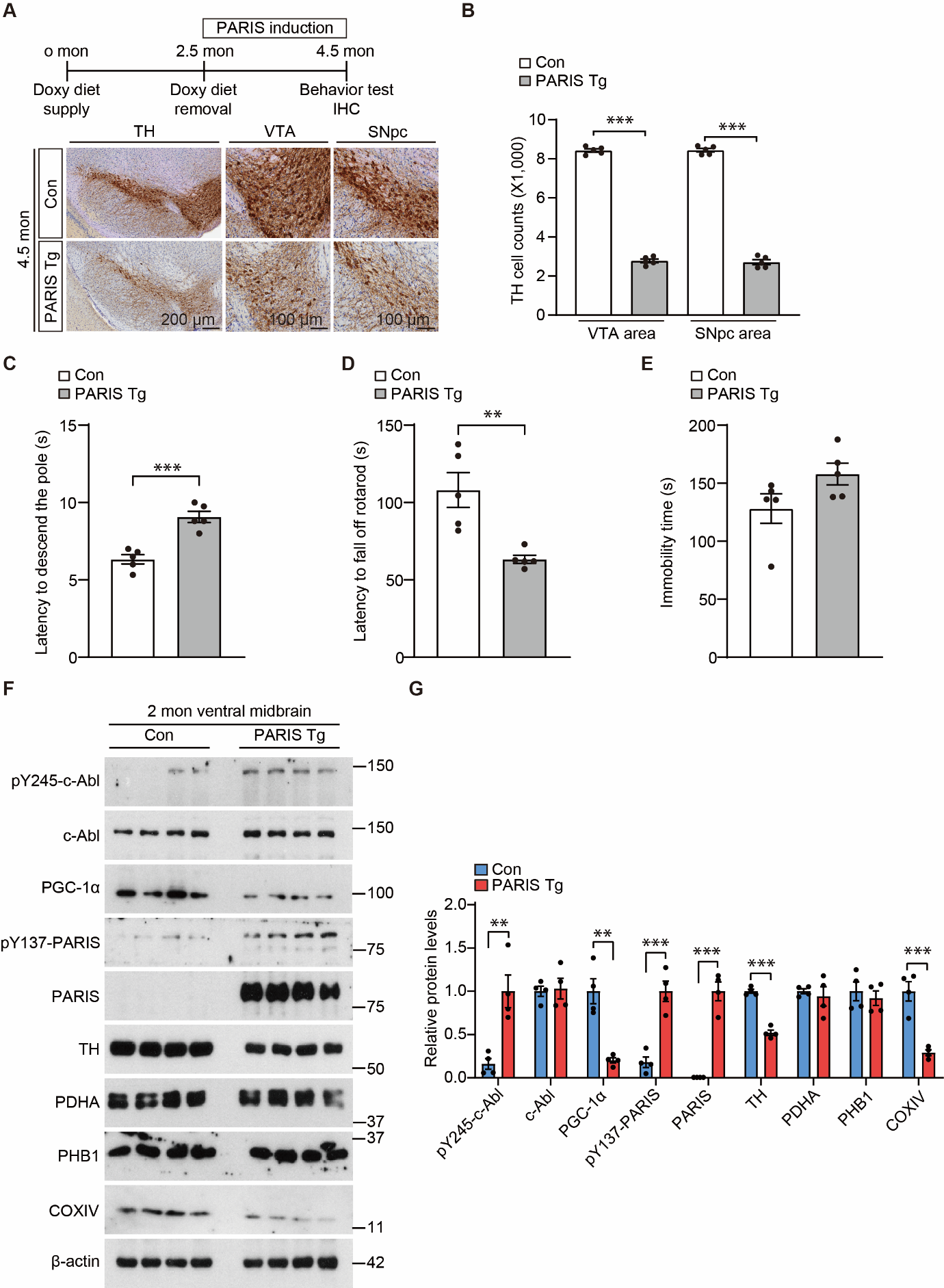
**

**Fig. S4. PD phenotypes in young adult PARIS Tg mice and assessment of early molecular alterations in the ventral midbrains of adolescent PARIS Tg mice.**

(A) Experimental PARIS induction protocol and representative tyrosine hydroxylase (TH) immunohistochemical staining for substantia nigra pars compacta (SNpc) and ventral tegmental area (VTA) of 4.5 month-old control or PARIS Tg mice (with PARIS induction initiated at 2.5 months of age). Scale bar: 200, 100 μm as indicated in the figure panels. Sacri, sacrifice.

(B) Stereological assessment of TH-positive dopaminergic neurons in SNpc, and VTA of 4.5-month-old control or PARIS Tg mice (*n* = 5 mice per mice).

(C) The pole test. The graph shows the differences of latency to descend the pole of 4.5-month-old control or PARIS Tg mice (*n* = 5 mice per group).

(D) The rotarod test. The graph shows the differences of latency to fall off the rotating-rod of 4.5-month-old control or PARIS Tg mice (*n* = 5 mice per group).

(E) The tail suspension test. Total immobility time during the test for 4.5-month-old control or PARIS Tg mice (*n* = 5 mice per group).

(F) Representative western blots showing pY245-c-Abl, c-Abl, PGC-1α, pY137-PARIS, PARIS, TH, and several mitochondrial proteins (PDHA, PHB1, and COXIV) levels in the ventral midbrains of 2-month-old control or PARIS Tg mice. b-actin was used as an internal loading control.

(G) Quantification analysis of relative pY245-c-Abl, c-Abl, PGC-1α, pY137-PARIS, PARIS, TH, PDHA, PHB1, and COXIV expression levels (normalized to those b-actin) in the experimental groups in panel F (*n* = 4 mice per group).

Data in all panels are mean ± standard error of the mean. ***P* <0.01, and ****P* <0.001, unpaired two-tailed student’s *t*-test.

**
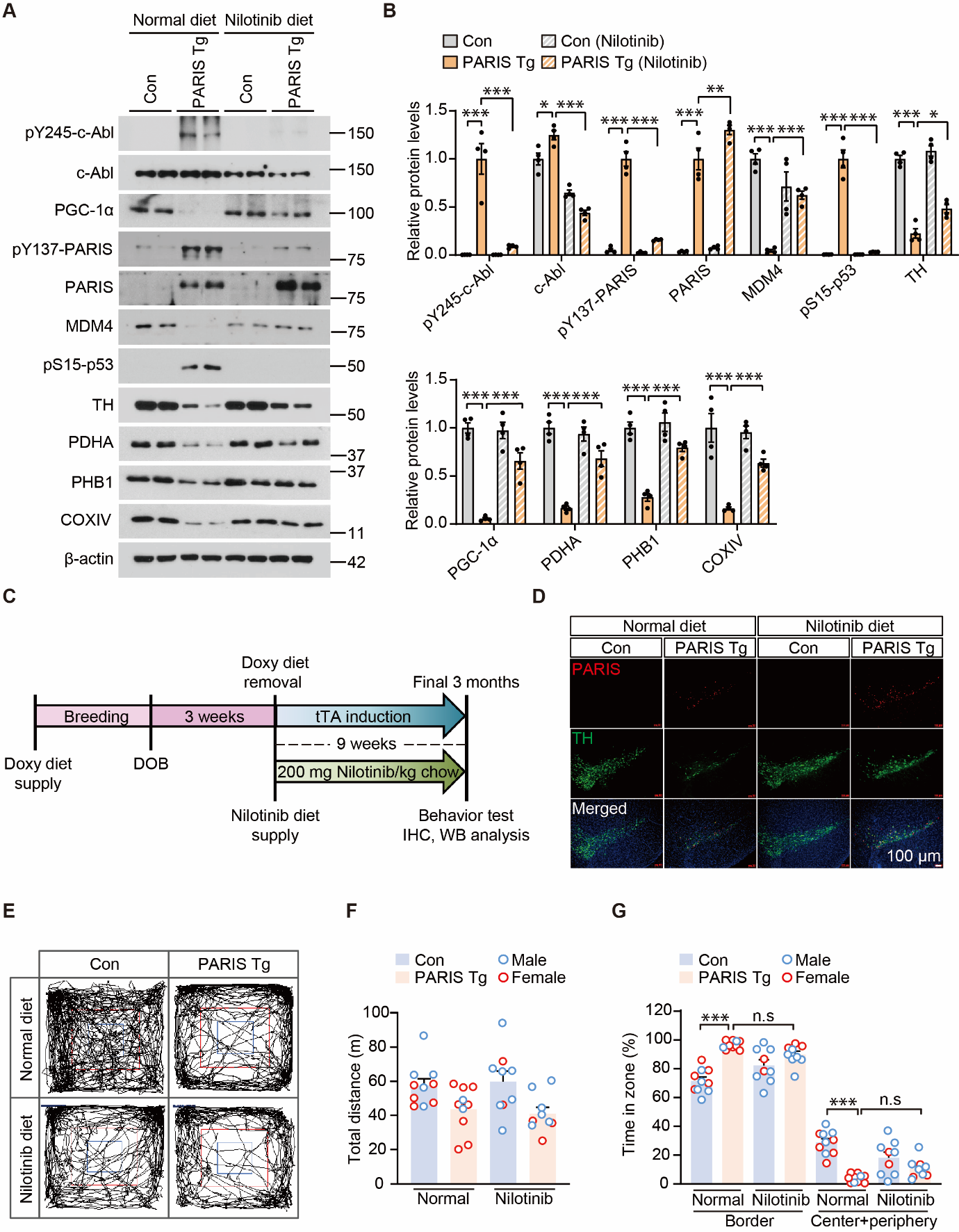
**

**Fig. S5. Preclinical evaluation of nilotinib in PARIS Tg mice**

(A) Representative western blots showing pY245-c-Abl, c-Abl, PGC-1α, pY137-PARIS, PARIS, MDM4, pS15-p53, TH, and several mitochondrial protein (pyruvate dehydrogenase [PDHA], prohibitin 1 [PHB1, and COXIV]) levels in the ventral midbrains of 3-month-old control or PARIS Tg mice fed with normal or nilotinib diets. β-actin was used as an internal loading control.

(B) Quantification of relative expression levels of the indicated proteins (normalized to those β-actin) in the experimental groups in panel A (*n* = 4 mice per group).

(C) Schematic diagram of experimental design to test an effect of nilotinib on 3-month-old PARIS Tg mice. Nilotinib diet (1 mg/day) was provided for 9 weeks starting from when the PARIS gene expression was induced by removing the doxycycline diet.

(D) Representative immunofluorescence images obtained by using anti-PARIS and -TH antibodies show an expression of PARIS in TH-dopaminergic neurons of 3-month-old PARIS Tg mice with or without nilotinib. Scale bar: 100 μm.

(E) Representative tracks of control or PARIS Tg mice with or without nilotinib treatment in the open field chamber over 15 min.

(F) The graph showing that total distance moved for 15 min in the open field chamber obtained from panel B (*n* = 10 mice per normal diet group and *n* = 9 mice per nilotinib diet group). Male and female mice are indicated by blue and red empty circles, respectively.

(G) Relative percentage of time spent in each zone, border, or center with periphery, obtained from the open field test in panel B (*n* = 10 mice per normal diet group and *n* = 9 mice per nilotinib diet group). Male and female mice are indicated by blue and red empty circles, respectively.

Data in all panels are mean ± standard error of the mean. **P* <0.05, ***P* <0.01, and ****P* <0.001, two-way analysis of variance (ANOVA) test followed by Tukey’s post hoc analysis. n.s, nonsignificant.

**
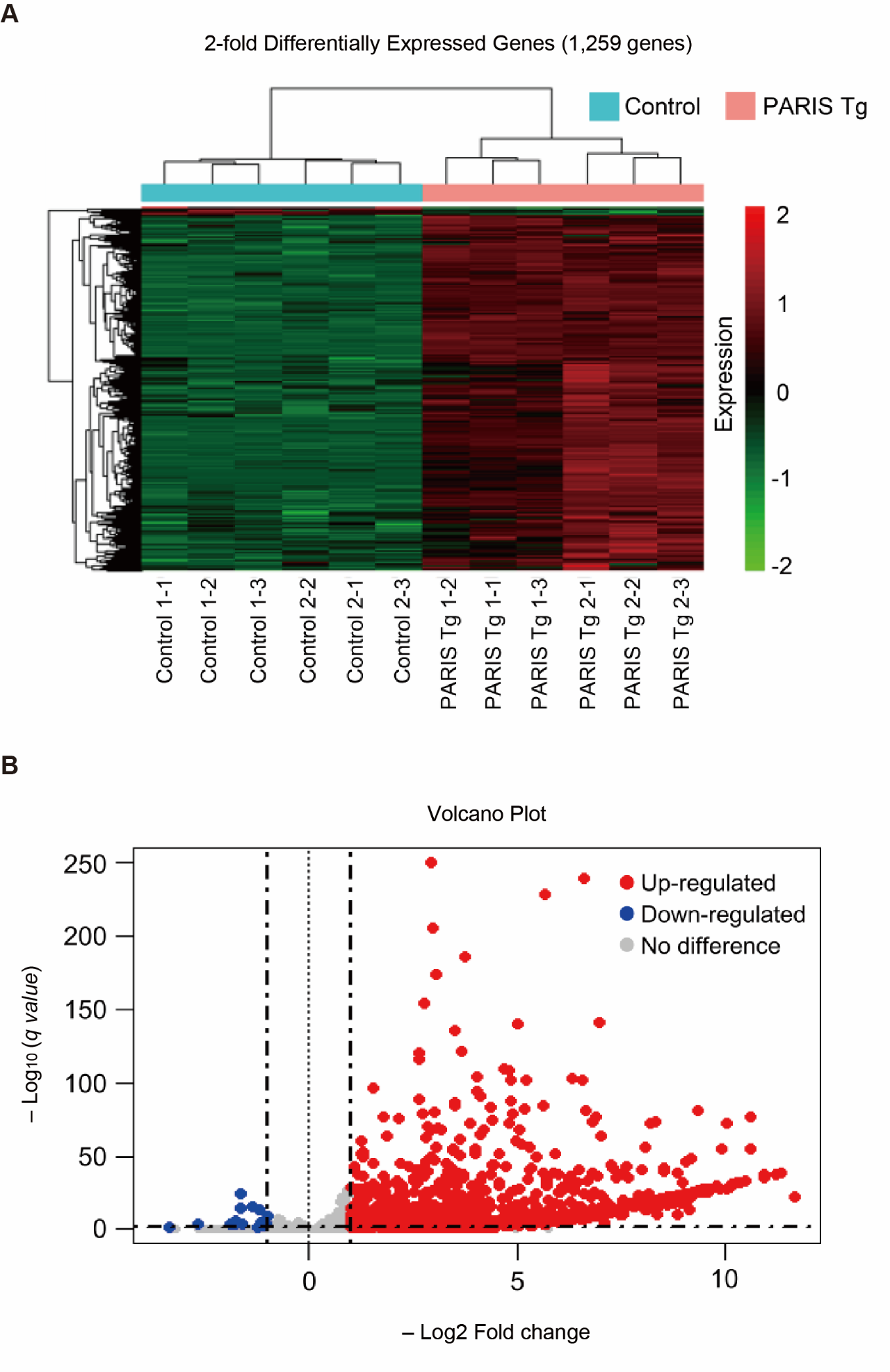
**

**Fig. S6. 2xfold DEGs display of PARIS-driven transcriptomic alterations in the midbrains of PARIS Tg mice**

(A) A heat map of 2× fold DEGs. 1,259 genes are DEGs between control and PARIS Tg mice.

(B) Volcano plot of 2× fold upregulated or downregulated DEGs in PARIS Tg mice midbrains (*q* value <0.05).

**
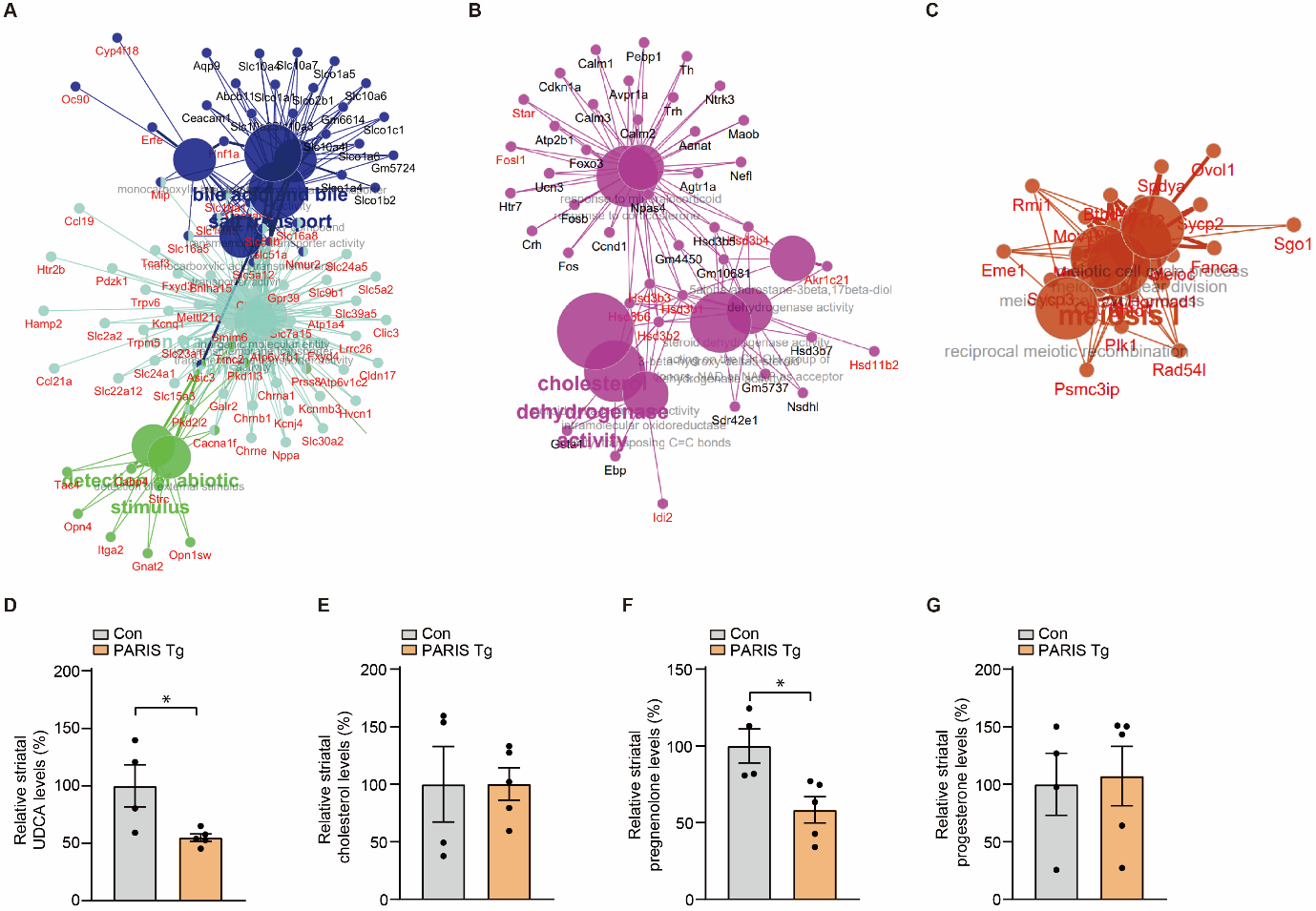
**

**Fig. S7. Network analysis of differentially expressed genes (DEGs) in the ventral midbrains of PARIS Tg mice and striatal lipid metabolite alteration**

(A) Enriched pathways and gene ontology have been visualized in ClueGO software. The blue color nodes represent bile acid and bile salt transport, mint color nodes represent ion channel membrane transport, and green nodes represent detection of abiotic stimulus pathway and enriched gene ontology in PARIS Tg mice, respectively. Demonstrated pathway associated genes were calculated based on the 4× fold DEGs with *P* <0.05.

(B) The three group of plum color nodes represent cholesterol dehydrogenase activity, steroid dehydrogenase activity, and response to corticosterone pathway in PARIS Tg mice. Demonstrated pathway associated genes were calculated based on the 4× fold DEGs with *P* <0.05.

(C) The red color nodes represent meiosis pathway, and enriched gene ontology in PARIS Tg mice. Demonstrated pathway associated genes were calculated based on the 2× fold DEGs with *P* <0.05.

(D, E, F, G) Quantification of ursodeoxycholic acid (UDCA), cholesterol, pregnenolone, and progesterone in the striatal tissues from 3 month-old PARIS Tg and littermate controls determined by LC-MS/MS (*n* = 4 mice for control, and 5 mice for PARIS Tg).

Data in all panels are mean ± standard error of the mean. **P* <0.05, unpaired two-tailed student’s *t*-test.

**
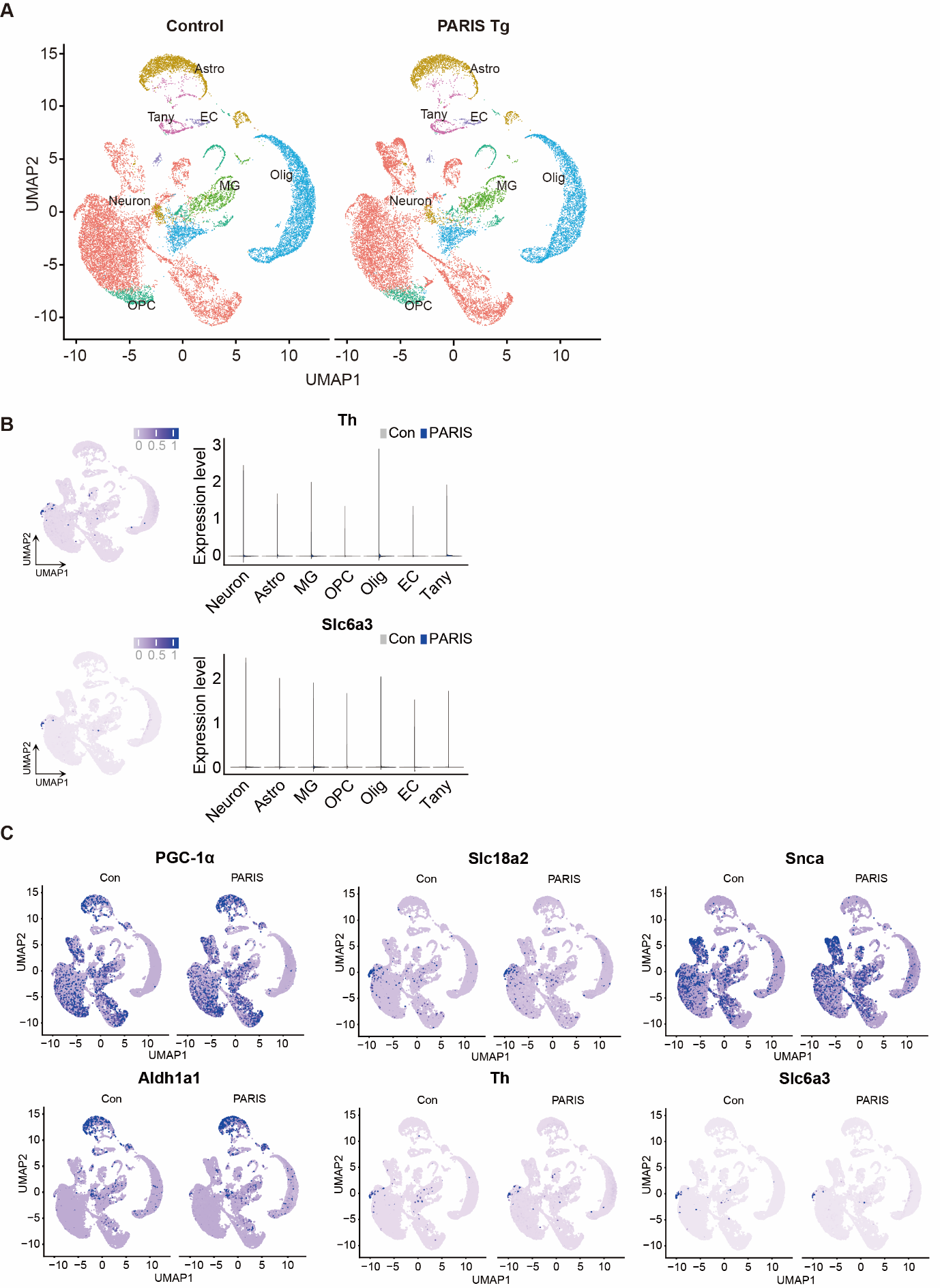
**

**Fig. S8. Cell clustering and transcriptional profiling for the ventral midbrains of PARIS Tg mouse model. (A)** UMAP plot clustering of 7 major cell-types from control and PARIS Tg. **(B)** PD-associated *Th*, and *Slc6a3* gene expression patterns and levels in different cell types are indicated on UMAP projections and violin plots, respectively. UMAP projects the gene expressions of PARIS Tg. **(C)** UMAP projection of the selected gene expression patterns in the ventral midbrains of the control and PARIS Tg, supplementing Figure 7D and Supplementary fig. 8B.

**
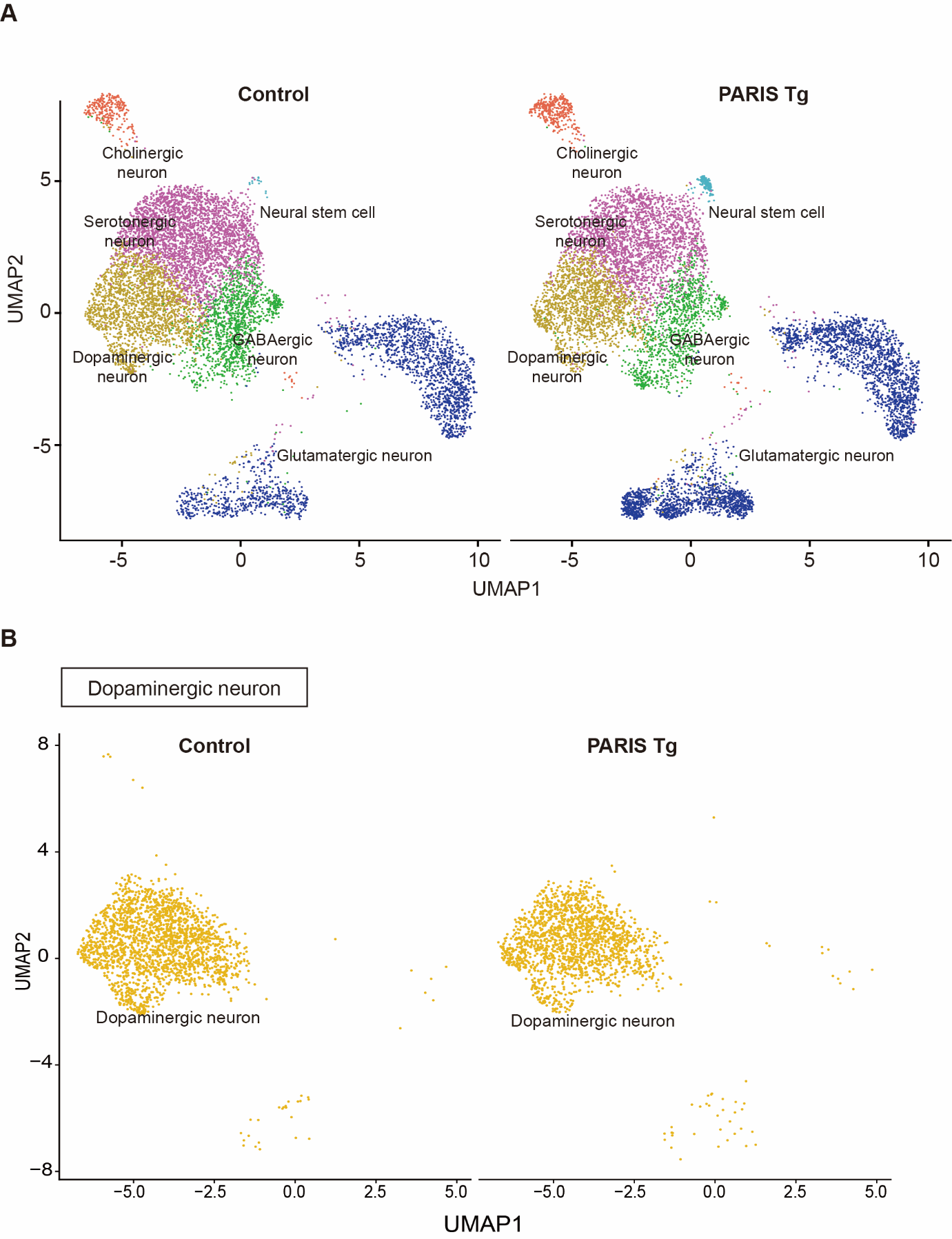
**

**Fig. S9. Neuron subpopulation clustering in PARIS Tg mouse model. (A)** UMAP visualization showing re-clustered 6 distinguished neuron subpopulations identified in the ventral midbrains from control and PARIS Tg, supplementing Fig. 8A. **(B)** UMAP projection of the dopaminergic neuron sub-cluster.

**
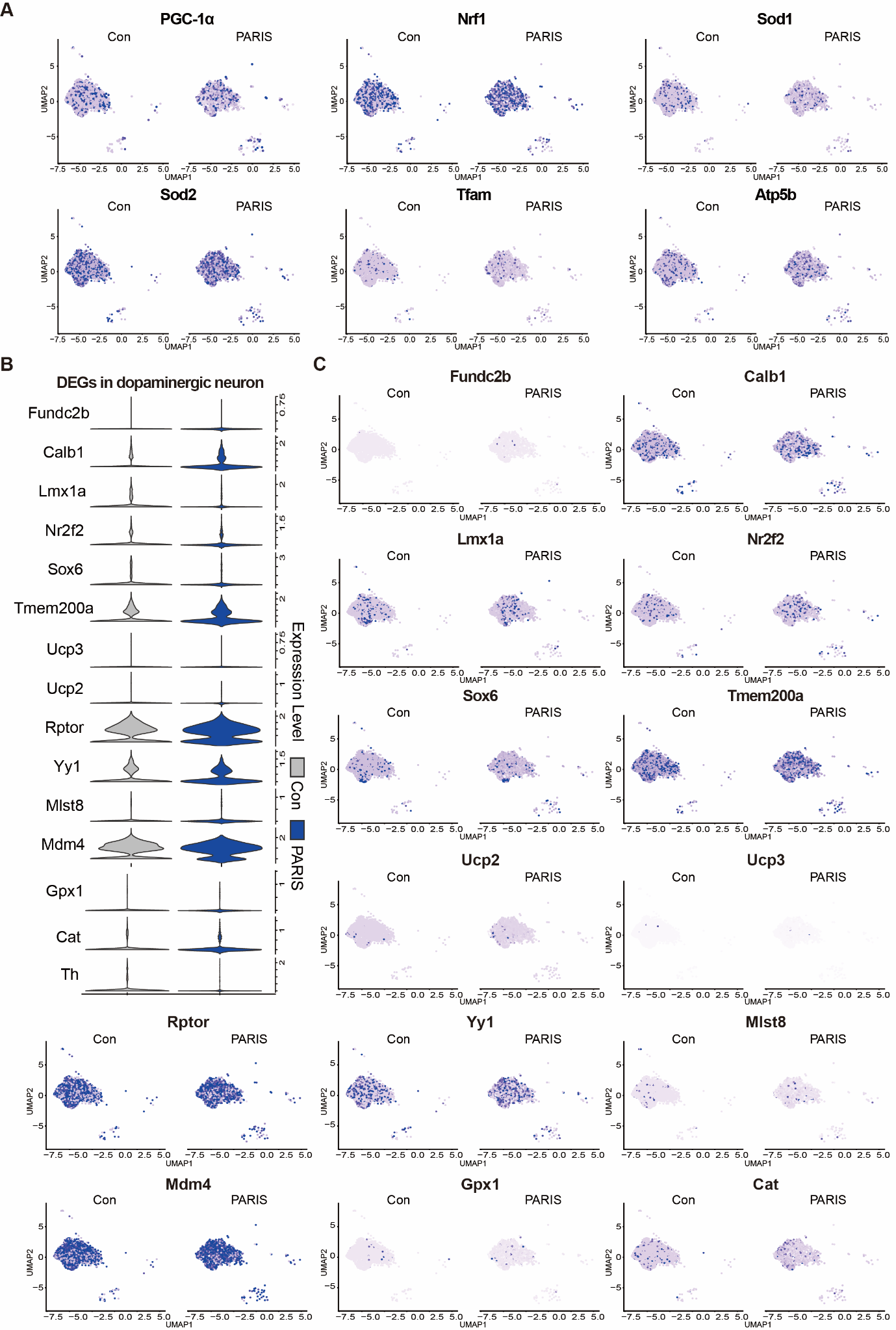
**

**Fig. S10. Transcriptional regulation in dopaminergic neurons of the ventral midbrains from control and PARIS Tg mouse model. (A)** UMAP projection of the gene expression patterns in dopaminergic neuron subcluster from both control and PARIS Tg, supplementing Fig. 8A. **(B)** Violin plots showing expression levels of genes which are potentially associated with mitochondria-related multiple cascades (PGC-1α regulated pathways, mTOR pathways, p53-dependent cell death) in dopaminergic neurons. **(C)** UMAP projection of the gene expression patterns in dopaminergic neuron cluster from both control and PARIS Tg, supplementing Fig. S10B.

**
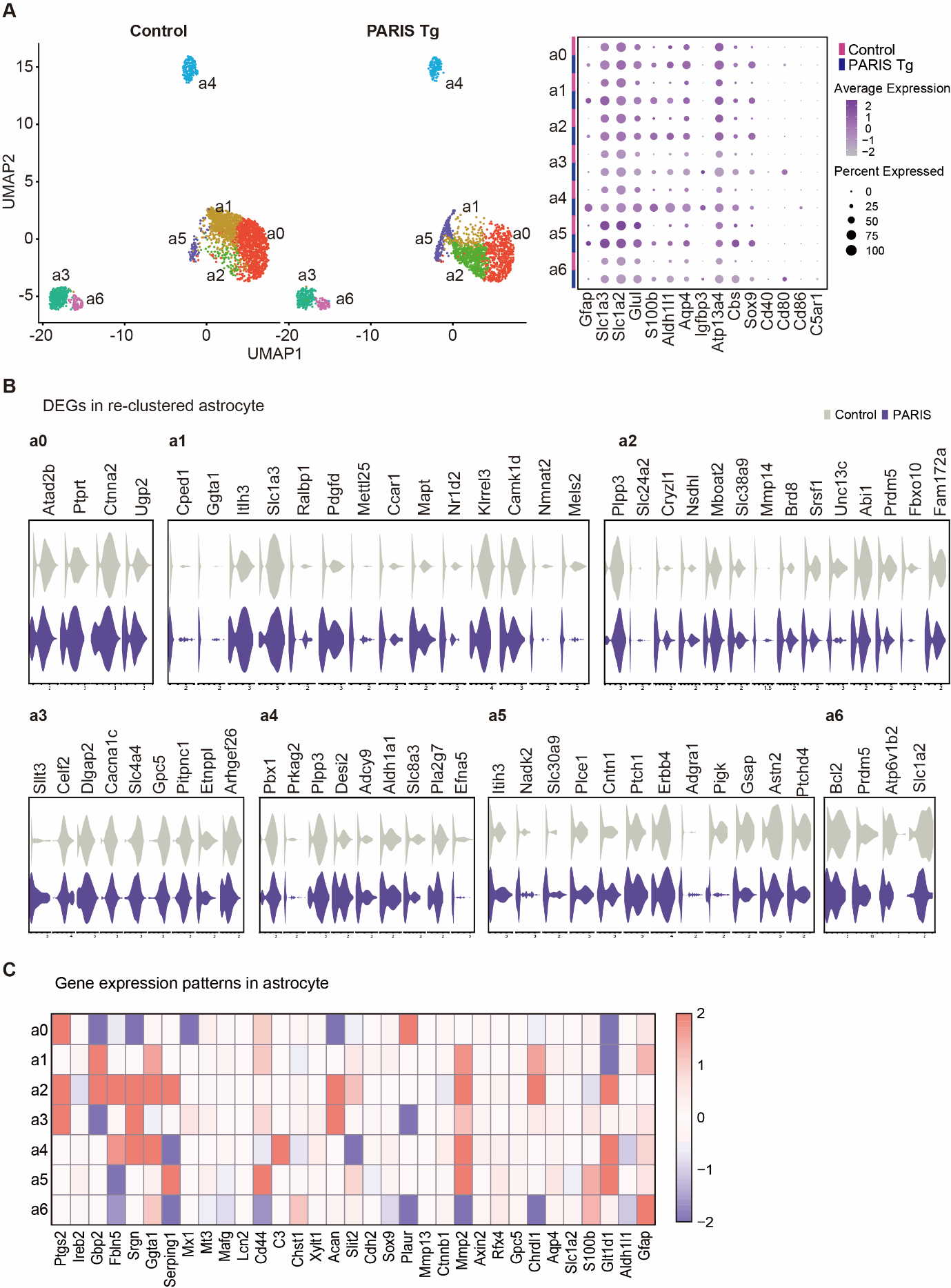
**

**Fig. S11. Astrocyte subpopulation clustering and transcriptional profiling for the ventral midbrains from control and PARIS Tg mouse model. (A)** UMAP visualization showing re-clustered 7 distinguished astrocyte subpopulations in both control and PARIS Tg, supplementing Fig. 9A. Dot plot shows the expressions of selected astrocyte markers in control and PARIS Tg. **(B)** Violin plot showing scaled expression level of the selected DEGs in each astrocyte clusters of the ventral midbrains from PARIS Tg and control. **(C)** Heatmap showing expression patterns of expanded astrocyte activation markers in re-clustered astrocyte subpopulations in PARIS Tg as compared to control, supplementing Fig. 9D.

**
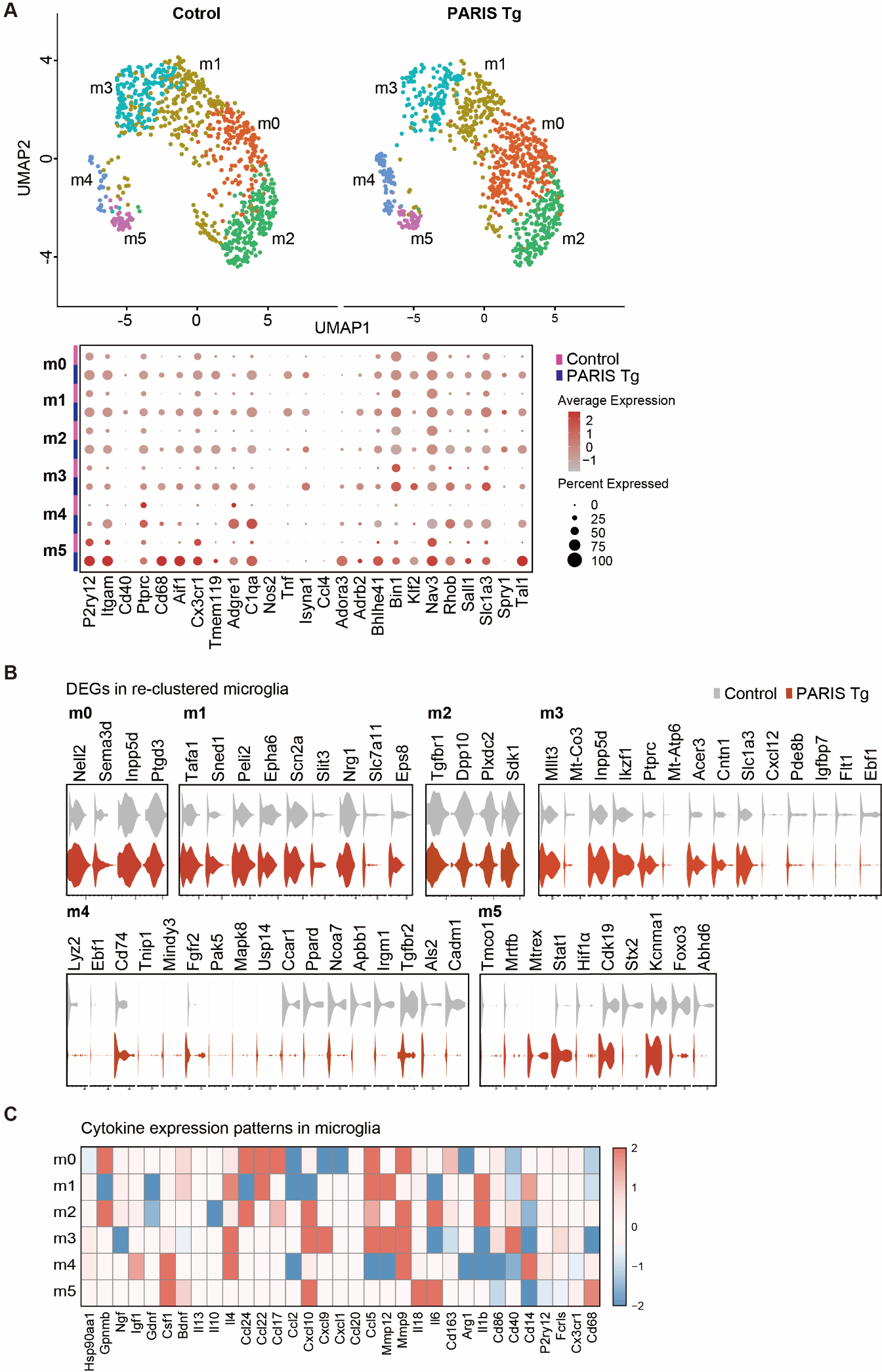
**

**Fig. S12. Microglia subpopulation clustering and transcriptional profiling for the ventral midbrains from control and PARIS Tg mouse model. (A)** UMAP visualization showing re-clustered 6 distinguished microglia subpopulations in both control and PARIS Tg, supplementing Fig. 9E. Dot plot shows the expressions of selected microglia markers in control and PARIS Tg. **(B)** Violin plot showing scaled expression level of the selected DEGs in each microglia cluster of PARIS Tg and control. **(C)** Heatmap showing expanded panels of cytokine expression in re-clustered microglia subpopulations in PARIS Tg as compared to those in control, supplementing Fig. 9H.

**
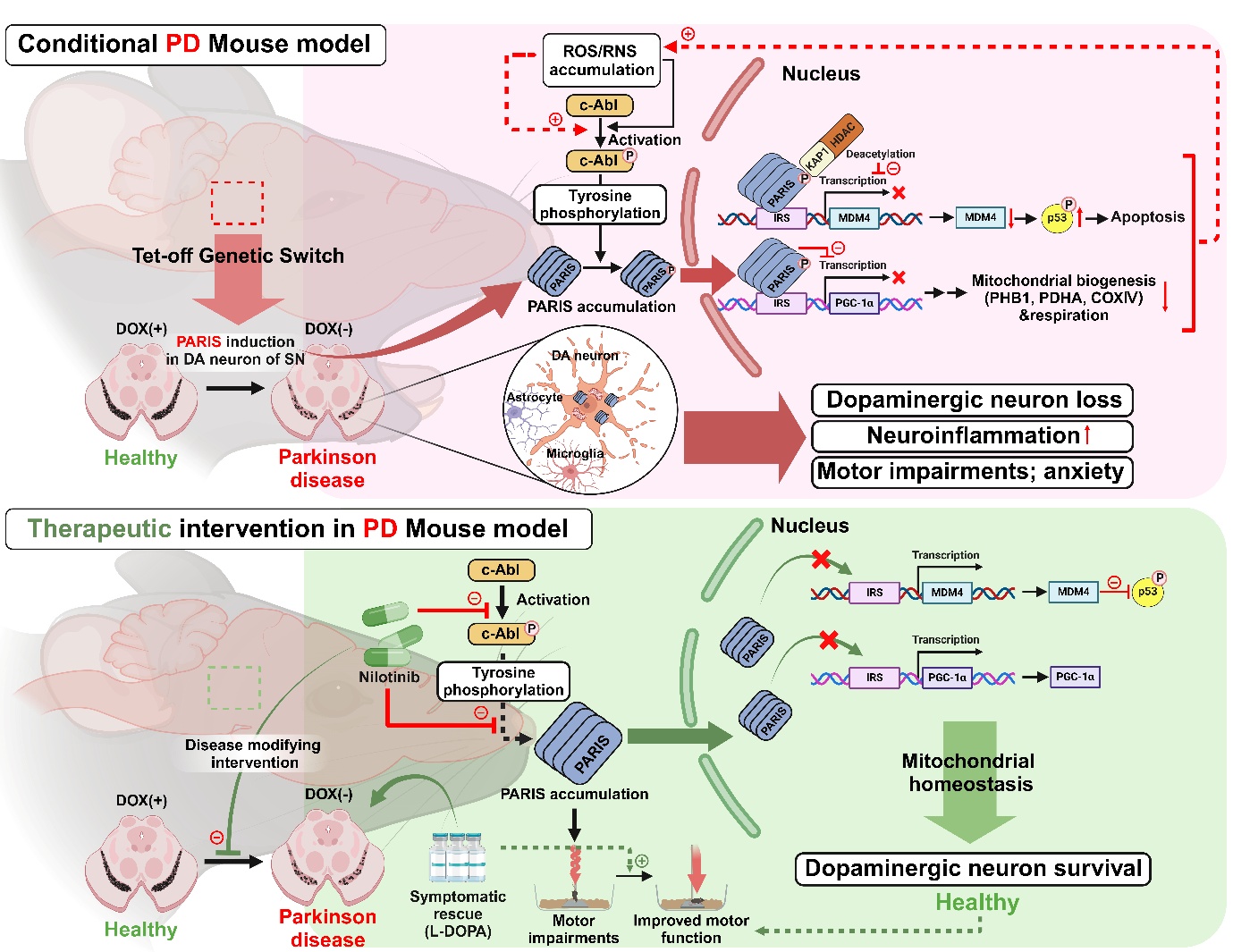
**

**Fig. S13. Schematic summary of pathogenic signaling and therapeutic interventions in a conditional PD mouse model**

Dopaminergic neuron-specific PARIS expression via a Tet-off genetic switch in transgenic mice models key pathological features of PD: progressive dopaminergic neuron loss, dopamine depletion, neuroinflammation, and motor impairment. PARIS accumulation activates c-Abl, leading to PARIS phosphorylation and repression of PGC-1α and MDM4. PGC-1α repression disrupts mitochondrial proteins (PHB1, PDHA, COXIV), while MDM4 repression activates p53, driving dopaminergic neuron death. Transcriptomic analysis confirms mitochondrial dysfunction and oxidative stress, linked to c-Abl activation. Therapeutic interventions showed that L-DOPA restores motor function, while c-Abl inhibition prevents neurodegeneration and neuroinflammation, preserving motor ability. Notably, c-Abl inhibition rescues PARIS-induced repression of PGC-1α and MDM4 and normalizes mitochondrial protein expression in PARIS Tg mice. Created in BioRender. Kim, J. (2025) https://BioRender.com/l14u754
